# Mechanistic studies on the CAN-mediated intramolecular cyclization of δ-aryl-β-dicarbonyl compounds

**DOI:** 10.3762/bjoc.9.167

**Published:** 2013-07-23

**Authors:** Brian M Casey, Dhandapani V Sadasivam, Robert A Flowers II

**Affiliations:** 1 Department of Chemistry, Lehigh University, Bethlehem, PA 18015, USA

**Keywords:** CAN oxidation, β-dicarbonyls, free radical, radical arylations, tetralones

## Abstract

The synthesis of 2-tetralones through the cyclization of δ-aryl-β-dicarbonyl substrates by using CAN is described. Appropriately functionalized aromatic substrates undergo intramolecular cyclizations generating 2-tetralone derivatives in moderate to good yields. DFT computational studies indicate that successful formation of 2-tetralones from δ-aryl-β-dicarbonyl radicals is dependent on the stability of the subsequent cyclohexadienyl radical intermediates. Furthermore, DFT computational studies were used to rationalize the observed site selectivity in the 2-tetralone products.

## Introduction

2-Tetralones are important intermediates or components of several natural products and biologically relevant molecules [1–7]. They are typically synthesized through transition-metal-mediated processes or preformed tetralin or naphthyl precursors [8–9]. Radical approaches can also be used to synthesize substituted 2-tetralones, and single-electron oxidations employing δ-aryl-β-dicarbonyl compounds have been carried out on arenes containing pendant β-ketoesters [10–11]. When Mn(III)-based oxidants are employed, secondary oxidations of the 2-tetralone products can occur [10–11].

**Scheme 1:**
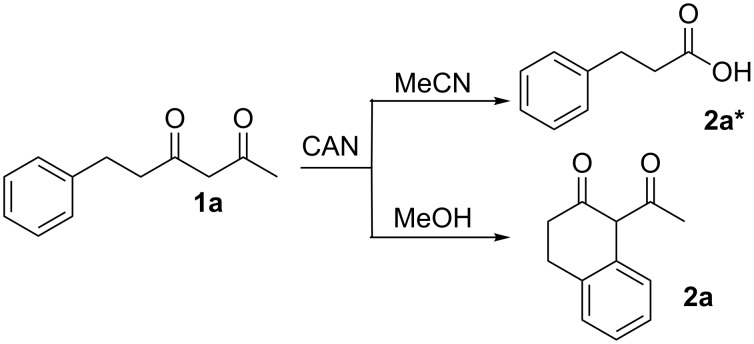
Oxidative conversion of 1,3-dicarbonyl compounds to carboxylic acids with CAN.

Cerium(IV) ammonium nitrate (CAN) is a versatile, inexpensive and nontoxic single-electron oxidizing reagent commonly used in organic synthesis [12–14]. In a previous study, we showed that when 6-phenyl-2,4-hexanedione (**1a**) is oxidized by CAN in MeCN in the absence of a radicophile, 3-phenylpropionic acid (2a*) is obtained exclusively over the cyclized 2-tetralone product **2a** (Scheme 1) [15]. Using this method, a variety of 1,3-dicarbonyl compounds can be mildly converted to carboxylic acids in moderate to excellent yields. In follow up studies, we found that 2-tetralone **2a** could be obtained as the major product when **1a** was oxidized by CAN in MeOH [16]. In addition, when 2.2 equivalents of CAN were employed, no products of secondary oxidations were obtained. Based on this observation, the single-electron oxidations of a variety of δ-aryl-β-dicarbonyl substrates with CAN in MeOH were performed to determine the scope of the reaction. DFT calculations were used to rationalize the observed impact of substitution on the δ-aryl ring on cyclization. The results of the synthetic and computational studies are presented herein.

## Results and Discussion

In an initial experiment, compound **1a** was treated with 2.2 equivalents of CAN in MeOH producing 2-tetralone **2a** in a 73% yield. To examine the breadth of this method, a series of δ-aryl-β-dicarbonyl substrates was prepared by a previously reported procedure [17]. As shown in Table 1, the intramolecular cyclization of δ-aryl-β-diketones with unsubstituted aryl rings (Table 1, entries 1 and 3) afforded 2-tetralone products in moderate to good yields. Additionally, cyclization of the β-ketoester **1b** proceeded efficiently, generating **2b** in an 85% yield.

**Table 1:** CAN-mediated oxidation of δ-aryl-β-dicarbonyl compounds in MeOH^a^.

Entry	Substrate	Product^b^	Yield (%)^c^

1	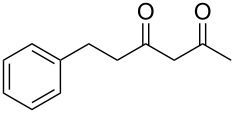 **1a**	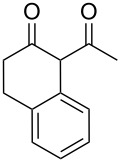 **2a**	73
2	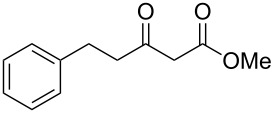 **1b**	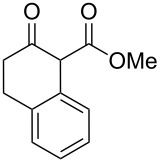 **2b**	85
3	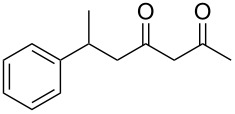 **1c**	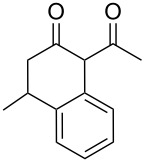 **2c**	59

^a^Reaction conditions: 1 equiv δ-aryl-β-dicarbonyl, 2.2 equiv CAN, MeOH, rt, N_2_, 4 h. ^b^Products exist predominantly in the enol form by ^1^H NMR. ^c^Isolated yield.

Previous work by Rickards and co-workers on a related system reported strong electronic effects when electron-donating substituents were incorporated onto the δ-aryl ring of the starting material [10–11]. To probe the impact of electron density of the δ-aryl ring on intramolecular cyclization, several substrates with either electron-donating or electron-withdrawing groups were synthesized and subjected to our reaction conditions. The results of these experiments are summarized in Table 2. As shown in Table 2, entry 1, dimethoxylated substrate **1d** oxidatively cyclized to the 2-tetralone derivative in a 76% yield. However, when only one methoxy group was incorporated onto the δ-aryl ring, the expected 2-tetralone derivative was obtained only when the methoxy group was at the *meta* position (Table 2, entry 4). For substrates **1e** and **1f** with the methoxy group at either the *ortho* or *para* position, respectively, methyl esters **2e** and **2f** (Table 2, entries 2 and 3) were the major products. Additionally, intramolecular cyclizations with electron-deficient δ-aryl rings (Table 2, entries 5 and 6) did not occur, and oxidation of **1h** and **1i** instead favored the formation of methyl esters **2h** and **2i**. Finally, the tricyclic product **2j** was generated in an isolated yield of 61% when substrate **1j** was oxidized. It is important to note that products **2g** and **2j** were isolated as single isomers. Moreover, the isomers formed from these reactions result from cyclization occurring at the more hindered carbon atom of the δ-aryl ring. This observed site selectivity is consistent with previous research by both MacMillan and Nicolaou on the α-arylation of aldehydes through organo-SOMO activation [18–21]. The preferential formation of these isomers will be discussed below.

**Table 2:** Impact of ring substituents on the CAN-mediated oxidation of δ-aryl-β-dicarbonyl compounds in MeOH^a^.

Entry	Substrate	Product^b^	Yield (%)^c^

1	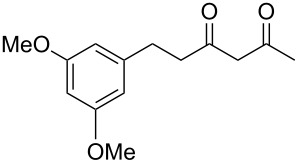 **1d**	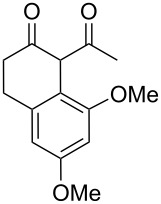 **2d**	76
2	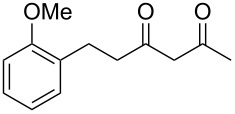 **1e**	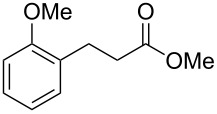 **2e**	–^d^
3	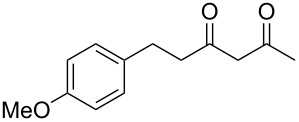 **1f**	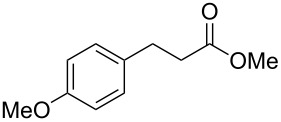 **2f**	–^d^
4	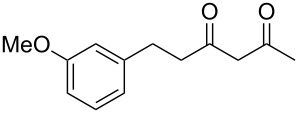 **1g**	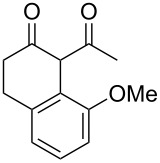 **2g**	83
5	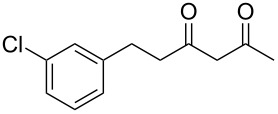 **1h**	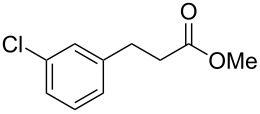 **2h**	–^d^
6	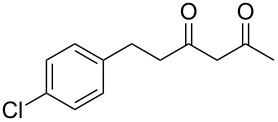 **1i**	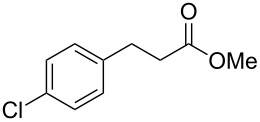 **2i**	–^d^
7	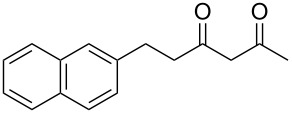 **1j**	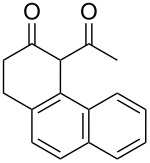 **2j**	61

^a^Reaction conditions: 1 equiv δ-aryl-β-dicarbonyl, 2.2 equiv CAN, MeOH, rt, N_2_, 4 h. ^b^Products exist predominantly in the enol form by ^1^H NMR. ^c^Isolated yield. ^d^GC data indicated that the major products (50–80% conversion) were the methyl esters. Attempts were not made to isolate the methyl esters.

While the nucleophilicity of alkyl radicals is well-documented [22–24], the radicals generated from β-dicarbonyl compounds have been shown to display more electrophilic character [25–27]. As a consequence, these radicals should favor coupling with more nucleophilic, electron-rich carbon centers. The observation that intramolecular cyclization did not occur in either of the electron-deficient substrates (compounds **1h** and **1i**) is consistent with electrophilic radical intermediates.

To obtain a better understanding of the impact of arene substitution on the intramolecular cyclization, DFT calculations were performed at UB3LYP/6-31G(d) level by using Gaussian 03/09 [28]. Previous research from our group has shown that the oxidation of the enol tautomer of a diketone initially forms a radical cation, which is deprotonated readily by MeOH to form the radical under the reaction conditions [15–16].

To probe the origin of the effect of substitution on cyclization, the key step of the reaction, namely the cyclization of the β-dicarbonyl radical onto the aromatic ring, was investigated. Energy barriers for the transition structures of the *ortho* cyclization leading to their corresponding product cyclohexadienyl radicals were determined.

All structures were fully optimized and identified as minima on potential energy surfaces with frequency calculations. Transition structures were identified with one imaginary frequency. Intrinsic reaction coordinate (IRC) calculations were performed to connect the transition structures to their respective minima on either side of the first-order saddle point. In some cases, the lowest energy structures obtained from IRCs were further optimized to obtain the minima.

For the aryl diketone radical, two conformers for the ketones were considered, one with the carbonyl groups *syn* to each other and the other with the carbonyl groups in the *anti* orientation. For every substrate, two transition structures were identified on the energy surface along the reaction coordinate. The first one is a rotational transition structure for the rotation around the C–C bond beta to the dicarbonyl to go from the minimum to a geometry from which an addition to the aromatic ring is viable. The second transition structure corresponds to the addition of the radical intermediate to the aromatic ring.

Recently, Houk, MacMillan and co-workers showed that for the organo-SOMO-catalyzed oxidative α-arylation of aldehydes, the preference for the attack of the intermediate enamine radical cation on the substituted aromatic ring leading to *ortho*/*para* cyclization depends on the greater stabilization of the intermediate cyclohexadienyl radical [19]. The oxidative cyclization of δ-aryl-β-dicarbonyl substrates should proceed through a similar intermediate. As a result, the same rationale can be applied here. While MacMillan and Houk performed detailed computational studies to explain the site-selective radical cyclizations with *m*-methoxylated rings, the selectivity of naphthyl substrates was not investigated.

**Figure 1:**
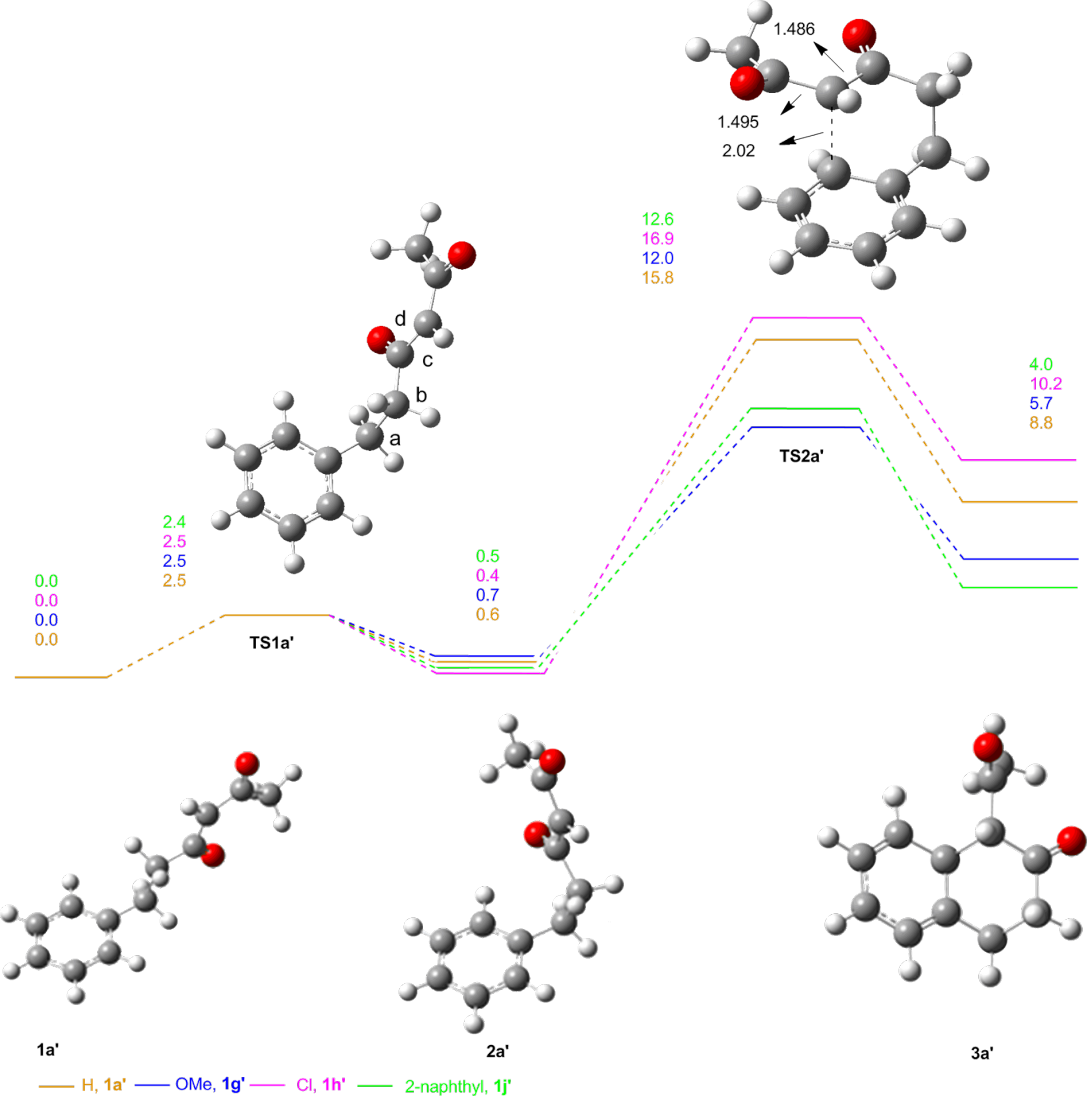
Energy diagram for the unsubstituted arene with the carbonyl groups *anti* to each other. For **TS1a’** the dihedral angle between atoms *abcd* = 6.4°. ^*^The energy values for **1a’**, **1g’**, **1h’** and **1j’** are shown. **1g”**, **1f’** and **1j”** were not included for clarity.

The energy values for the cyclization of all substituents are given in Table 3, and their corresponding energy diagrams are given in Figure 1. For unsubstituted **1a’**, the initial rotational transition structure **TS1a’** has a barrier of 2.5 kcal/mol, and the corresponding radical intermediate **2a’** is 0.6 kcal/mol above **1a’**. For the radical addition to the aromatic ring, the energy barrier is 15.8 kcal/mol, and the product cyclohexadienyl radical **3a’** is 8.8 kcal/mol higher in energy. The *syn* isomer of **1a’** is 4.5 kcal/mol higher in energy compared to the *anti* isomer (see Table S2 in Supporting Information File 1). Only the energies of the *anti* isomers are discussed here.

**Table 3:** Energies (R. E. kcal/mol) of calculated structures. Energies are relative to the open form of the radical.

	R. E.^a^	R. E. + ZPVE^b^	low frequency^c^

**1a'**	0.0	0.0	21.4
**TS1a'**	2.3	2.5	46.7i
**2a'**	0.2	0.6	19.7
**TS2a'**	15.0	15.8	477.2i
**3a'**	7.7	8.8	35.4
**1g'**	0.0	0.0	13.30
**TS1g'**	2.4	2.5	41.8i
**2g'**	0.4	0.7	16.6
**TS2g'**	11.0	12.0	446.3i
**3g'**	4.2	5.7	37.5
**1g''**	0.0	0.0	13.3
**TS1g"**	2.3	2.4	45.0i
**2g"**	0.3	0.6	13.5
**TS2g"**	13.1	13.9	437.5i
**3g"**	7.3	8.5	34.2
**1f'**	0.0	0.0	21.3
**TS1f'**	2.4	2.4	42.3i
**2f'**	0.2	0.5	15.9
**TS2f'**	15.8	16.4	473.0i
**3f'**	9.1	10.0	36.1
**1h'**	0.0	0.0	15.1
**TS1h'**	2.3	2.4	40.3i
**2h'**	0.2	0.4	19.1
**TS2h'**	16.2	16.9	489.8i
**3h'**	8.8	10.2	36.2
**1j'**	0.0	0.0	17.45
**TS1j'**	2.4	2.5	42.1i
**2j'**	0.2	0.5	16.5
**TS2j'**	11.8	12.6	445.9i
**3j'**	2.4	4.0	34.9
**1j"**	0.0	0.0	17.5
**TS1j"**	2.2	2.3	41.3i
**2j"**	0.3	0.6	11.5
**TS2j"**	13.4	14.1	463.8i
**3j"**	4.9	6.2	35.7

^a^UB3LYP/6-31G(d) geometry optimized. ^b^From (a) with unscaled zero-point vibrational energy (ZPVE) corrections. ^c^Low or imaginary frequencies (cm^−1^).

In the case of the electron-rich *m*-methoxy substituent **1g’**, the energy barrier for the cyclization is 12.0 kcal/mol, which is approximately 4 kcal/mol lower than **TS2a’**. Due to the electrophilic character of the diketo radical intermediate, electron-rich systems are expected to favor cyclization. Similarly, the cyclized cyclohexadienyl radical **3g’** is approximately 3.0 kcal/mol more stable compared to **3a’**. The stability of the cyclized radical is reflected in the activation barrier, and rotational transition structure **TS1g’** has a barrier of 2.5 kcal/mol. For the electron-withdrawing chloro-substituted arene **1h’**, the energy barrier for the cyclization on the aromatic ring is 16.9 kcal/mol, nearly 5 kcal/mol higher compared to the *m*-methoxy substituent. The corresponding cyclohexadienyl radical **3h’** is 10.2 kcal/mol higher in energy compared to the parent β-dicarbonyl radical **1h’**. The rotational transition structure has an energy barrier of 2.4 kcal/mol, which is similar to other radical intermediates. The higher barrier for cyclization is consistent with synthetic data showing that no cyclized product is obtained in this case. In the case of the naphthyl system (**1j’**), the energy barrier for the cyclization is 12.6 kcal/mol. The cyclized product radical **3j’** is only 4.0 kcal/mol higher in energy when compared to the parent aryl diketone radical, and the rotational transition structure has a comparable barrier of 2.5 kcal/mol.

**Figure 2:**
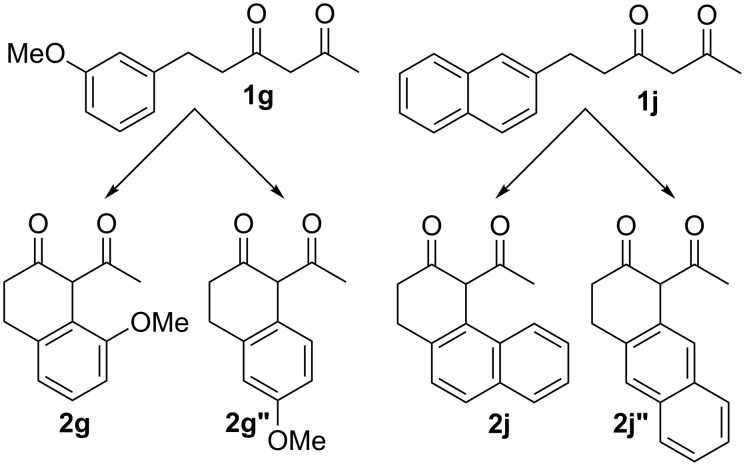
Possible products from the *ortho* cyclization of **1g** and **1j**.

While it is evident why electron-rich systems should more readily cyclize, the observed site selectivity in products **2g** and **2j** warranted further investigation. For these substrates, there are two possible sites of *ortho* cyclization (Figure 2). For the *m*-methoxy substrate **1g**, the energy barrier for the cyclization (**TS2g’’**), which would lead to product **2g”**, is 1.9 kcal/mol higher relative to the other *ortho* cyclization (**TS2g’**). This finding is consistent with the synthetic observation that **3g’** is the only product observed. A similar trend was observed for the 2-naphthyl substrate **1j**. The barrier for **TS2j”**, which would form anthracene-derived product **2j”**, is 1.5 kcal/mol higher than **TS2j’** and provides a rationale for the selective formation of the phenanthrene-derived product (**2j**).

Finally, in order to better understand how the position of the methoxy group on the δ-aryl ring affects the formation of the β-tetralone product, the energies of the intermediates for the *p*-methoxy substrate **1f** were calculated. The energy barrier for the cyclization (**TS2f’**) is 16.4 kcal/mol, 4.4 kcal/mol higher than the corresponding **TS2g’**. Furthermore, the product cyclohexadienyl radical **3f’** is 10.0 kcal/mol less stable than the parent **1f’**, a value very close to the 10.2 kcal/mol for the electron-poor *m*-chloro substrate **3h’**. Taken together, these data support the experimental observations that oxidative cyclization occurs only for the methoxy substrate substituted at the *meta* position.

Overall the DFT calculations show that the origin of the reactivity as well as the selectivity in these reactions depends on the stability of the product cyclohexadienyl radical, which is reflected in the activation barriers (**TS2’s**) for the cyclization, consistent with previous studies of MacMillan and Houk [19]. The *m*-methoxy substituent on the aromatic ring provides the lowest barrier for cyclization, and the corresponding cyclized cyclohexadienyl radical is more stable. Conversely, the electron-withdrawing *m*-chloro substituent has the highest barrier among the systems studied and leads to the least stable cyclohexadienyl radical. For the unsubstituted arene substrate, the energy barrier to cyclization as well as the energy of the cyclohexadienyl radical intermediate falls between the calculated values for the *m*-chloro and *m*-methoxy systems. For the 2-naphthyl system, delocalization of the cyclized radical intermediate results in a more stable product and hence a lower barrier providing a pathway to formation of phenanthrene-derived **2j**.

In a previous study by our research group, the rates of oxidation of several β-diketones and their related silyl enol ethers by CAN and the more lipophilic ceric tetra-*n*-butylammonium nitrate (CTAN) were measured in MeOH, MeCN and CH_2_Cl_2_ by using stopped-flow spectrophotometry [16]. In these experiments, initial oxidation of substrates generated radical cation intermediates. The rates of formation and subsequent decay of these radical cations were measured in all three solvents. The results from these studies [15–16] provide two important insights into the mechanism of the oxidation of δ-aryl-β-dicarbonyl compounds in MeOH. First, MeOH is intimately involved in the decay of the initial radical cation through solvent-assisted deprotonation. Second, intramolecular cyclization of **1a’** occurs after the rate-limiting step of the reaction.

**Scheme 2:**
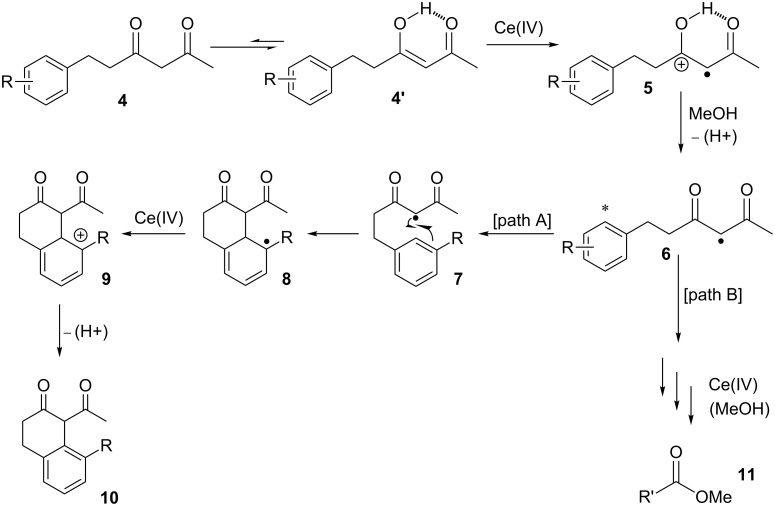
Proposed mechanism for the conversion of δ-aryl-β-dicarbonyl compounds to β-tetralones (path A) and methyl esters (path B).

Based on the synthetic and computational data presented herein and findings from previous studies, the mechanism in Scheme 2 is proposed for the oxidation of δ-aryl-β-dicarbonyl compounds in MeOH with CAN. Initial oxidation of the enol tautomer (**4’**) by CAN produces protonated radical **5**. Intermediate **5** is deprotonated by MeOH to radical species **6**. When the radical contains a δ-aryl group with an appropriate substitution at the *meta* position, path A is followed. Intramolecular cyclization of **7** occurs through radical addition to the aromatic ring forming intermediate **8**. As demonstrated by the oxidation of substrate **1j**, intramolecular cyclization of this radical occurs at the more electron-rich carbon atom of the δ-aryl rings. A second equivalent of CAN oxidizes **8** to cation **9**. Rearomatization through deprotonation of intermediate **9** yields the 2-tetralone derivative **10**. Conversely, when the δ-aryl ring has electron-withdrawing substituents (Table 2, entries 5 and 6), the reaction follows path B [16,29–31].

## Conclusion

A protocol for the conversion of δ-aryl-β-tetralones using CAN has been developed. The Ce(IV)-mediated synthesis of 2-tetralones has short reaction times and affords the desired products in moderate to very good yields under mild conditions. While 2-tetralones were not generated for all substrates, cyclization does occur for the unsubstituted arene **1a** suggesting that the electrophilicity of the radical provides some driving force for the cyclization. The DFT computational studies indicated that the formation of 2-tetralones from the cyclization of δ-aryl-β-dicarbonyl radicals is dependent on the stability of the product cyclohexadienyl radicals.

## Experimental

**General methods and materials**. Methanol (MeOH) was degassed with argon and dried with activated 3 Å molecular sieves prior to use. THF was purified with a Pure Solv solvent purification system from Innovative Technology Inc. CAN was purchased commercially and used without further purification. The glassware was flame dried prior to use. Unless otherwise stated, reactions were performed under an inert atmosphere of nitrogen. Products were separated by using prepacked silica gel columns with a gradient elution of ether/hexanes in an automated CombiFlash^®^ Rf system from Teledyne Isco, Inc. All new compounds were characterized by ^1^H NMR, ^13^C NMR, GC–MS, IR, and LC–HRMS. Known compounds were characterized by ^1^H NMR, ^13^C NMR and GC–MS. ^1^H NMR and ^13^C NMR spectra were recorded on a Bruker 500 MHz spectrometer. Mass spectra were obtained by using a HP 5890 series GC–MS instrument. A Satellite FTIR from Thermo-Mattson was used to obtain IR spectra. LC–HRMS data were recorded at the Mass Spectrometry Facility at Notre Dame University.

**General procedure for the synthesis of δ-aryl-β-dicarbonyl compounds 1a–j**. Sodium hydride (11 mmol) was suspended in 25 mL of THF and cooled to 0 °C. Next, 10 mmol of 2,4-pentanedione (or methyl acetoacetate for **1b**) was added dropwise to the flask, evolving H_2_ gas and forming an opaque, white solution. After stirring for 10 min, 10.5 mmol of butyllithium was added dropwise forming a clear yellow solution, which was stirred for an additional 10 min. The appropriate organohalide (11 mmol) was dissolved in 2 mL of THF and rapidly injected into the reaction at 0 °C. The reaction mixture was warmed gradually to room temperature over 30 min. The reaction was slowly quenched with an HCl solution (2 mL of concentrated HCl in 5 mL H_2_O). The organic layer was separated, and the aqueous layer was washed three times with ether. The organic layers were combined, washed with brine, dried with MgSO_4_, filtered, and concentrated. The crude product was purified by automated flash chromatography.

**General procedure for the oxidation of δ-aryl-β-dicarbonyl compounds with CAN in MeOH**. CAN (1.1 mmol) was dissolved in 4 mL MeOH. This CAN solution was then added dropwise in 1 min to the δ-aryl-β-dicarbonyl compound (0.5 mmol), which was dissolved in 15 mL of MeOH. The reaction was stirred for 30 min. The solvent was then removed by rotary evaporation. Ice-cold H_2_O (15 mL) was poured into the reaction and extracted three times with ether. The organic layers were combined, dried with MgSO_4_, filtered, and concentrated. The crude product was purified by automated flash chromatography.

## Supporting Information

File 1 Characterization data for all compounds, copies of ^1^H and ^13^C NMR spectra of final products, computational details, absolute energies, and Cartesian coordinates of all optimized structures.
