# Supplementary material for: Mechanistic studies on the CAN-mediated intramolecular cyclization of δ-aryl-β-dicarbonyl compounds
Source: Beilstein J Org Chem. 2013 Jul 23;9:1472–9. doi: 10.3762/bjoc.9.167 (PMC3740710; doi:10.3762/bjoc.9.167)
Supplement: File 1 — Characterization data for all compounds, copies of 1H and 13C NMR spectra of final products, computational details, absolute energies, and Cartesian coordinates of all optimized structures. [file Beilstein_J_Org_Chem-09-1472-s001.pdf]

Supporting Information

for

Mechanistic studies on the CAN-mediated intramolecular cyclization of δ-aryl-β-dicarbonyl compounds

Brian M. Casey, Dhandapani V. Sadasivam and Robert A. Flowers II*

Address: Department of Chemistry, Lehigh University, Bethlehem, PA 18015, USA

Email: Robert A. Flowers II - rof2@lehigh.edu

* Corresponding author

Characterization data for all compounds, copies of ^{1}H and ^{13}C NMR spectra of final products, computational details, absolute energies, and Cartesian coordinates of all optimized structures

Table of Contents:

S2. Product characterizations

S5. Absolute energies of structures related to **1x’** (Table S1)

S6. Absolute energies and relative energies of structures related to **1a** (Table S2)

S7–S10. Transition state geometries of **TS1x’** and **TS2x’**

S11–S34. Cartesian coordinates of all stationary points

S35–S67. NMR spectra of starting materials and products

Characterization of starting materials and products

6-Phenyl-2,4-hexanedione (1a). Clear, colorless oil. ^{1}H NMR ($CDCl_3$, 500 MHz) – (enol tautomer) δ 15.46 (br. s, 1H), 7.32-7.27 (m, 2H), 7.24-7.17 (m, 3H), 5.49 (s, 1H), 2.94 (t, 2H, J = 8.1 Hz), 2.60 (t, 2H, J = 8.2 Hz), 2.05 (s, 3H). ^{13}C NMR ($CDCl_3$, 125 MHz) – δ 193.2, 191.0, 140.6, 128.8, 128.5, 128.5, 128.4, 128.3, 128.2, 126.4, 126.2, 100.0, 58.0, 45.2, 40.0, 38.1, 31.4, 29.4, 24.8. MS [*m/z* (rel int)] 190 (M^+, 72), 172 (11), 129 (10), 104 (67), 91 (86), 85 (100), 77 (31).

Methyl 3-oxo-5-phenyl-pentanoate (1b). Clear, colorless oil. ^{1}H NMR ($CDCl_3$, 500 MHz) – (keto tautomer) δ 7.31-7.27 (m, 2H), 7.22-7.17 (m, 3H), 3.72 (s, 3H), 3.44 (s, 2H), 2.95-2.86 (m, 4H). ^{13}C NMR ($CDCl_3$, 125 MHz) – δ 201.7, 167.5, 140.4, 128.5, 128.4, 128.3, 126.2, 52.4, 49.2, 44.5, 29.4. MS [*m/z* (rel int)] 206 (M^+, 28), 188 (41), 174 (25), 133 (50), 128 (34), 104 (100), 91 (100), 77 (47).

6-Methyl-6-phenyl-2,4-hexanedione (1c). Clear, light yellow oil. ^{1}H NMR ($CDCl_3$, 500 MHz) – (enol tautomer) δ 15.52 (br. s, 1H), 7.34-7.29 (m, 2H), 7.26-7.20 (m, 3H), 5.43 (s, 1H), 3.29 (apparent sext, 1H, J = 7.1 Hz), 2.61 (dd, 1H, J = 14.7, 6.8 Hz), 2.49 (dd, 1H, J = 14.3, 8.2 Hz), 2.02 (s, 3H), 1.32 (d, 3H, J = 7.0 Hz). ^{13}C NMR ($CDCl_3$, 125 MHz) – δ 202.9, 201.8, 191.9, 191.6, 145.8, 145.6, 128.4, 128.3, 126.6, 126.2, 100.6, 58.3, 51.6, 46.6, 46.5, 36.7, 36.6, 35.1, 35.1, 24.9, 24.8, 21.6, 21.5. MS [*m/z* (rel int)] 204 (M^+, 36), 186 (9), 143 (18), 118 (41), 105 (100), 91 (23), 85 (54), 77 (32).

6-(3,5-Dimethoxyphenyl)-2,4-hexanedione (1d) Clear oil. ^{1}H NMR ($CDCl_3$, 500 MHz) – (enol tautomer) δ 15.44 (br. s, 1H), 6.37-6.26 (m, 3H), 5.46 (s, 1H), 3.75 (s, 6H), 2.85 (t, 2H, J = 7.9 Hz), 2.56 (t, 2H, J = 7.9 Hz), 2.02 (s, 3H). ^{13}C NMR ($CDCl_3$, 125 MHz) – δ 193.3, 190.9, 160.8, 143.1, 106.3, 100.0, 98.1, 55.2, 39.8, 31.7, 24.7. MS [*m/z* (rel int)] 250 (M^+, 21), 232 (9), 165 (100), 151 (14), 91 (12), 85 (16), 77 (13).

6-(2-Methoxyphenyl)-2,4-hexanedione (1e). Clear, light yellow oil. ^{1}H NMR ($CDCl_3$, 500 MHz) – (enol tautomer) δ 15.47 (br. s, 1H), 7.23-7.18 (m, 1H), 7.15-7.12 (m, 1H), 6.91-6.83 (m, 2H), 5.49 (s, 1H), 3.83 (s, 3H), 2.92 (t, 2H, J = 7.7Hz), 2.58 (t, 2H, J = 7.7Hz), 2.05 (s, 3H). ^{13}C NMR ($CDCl_3$, 125 MHz) – δ 193.9, 191.0, 157.4, 129.8, 129.0, 127.5, 120.4, 110.2, 99.8, 55.18, 38.3, 26.6, 24.9. MS [*m/z* (rel int)] 220 (M^+, 27), 146 (13), 134 (33), 121 (77), 91 (100), 85 (46), 77 (33).

6-(4-Methoxyphenyl)-2,4-hexanedione (1f). Clear, light yellow oil. ^{1}H NMR ($CDCl_3$, 500 MHz) – (enol tautomer) δ 15.46 (br. s, 1H), 7.13-7.09 (m, 2H), 6.85-6.81 (m, 2H), 5.47 (s, 1H), 3.79 (s, 3H), 2.88 (t, 2H, J = 7.6 Hz), 2.56 (t, 2H, J = 7.6 Hz), 2.04 (s, 3H). ^{13}C NMR ($CDCl_3$, 125 MHz) – δ 193.2, 191.1, 158.0, 132.7, 129.2, 129.2, 113.9, 113.8, 100.0, 55.2, 40.2, 30.6, 24.9. MS [*m/z* (rel int)] 220 (M^+, 39), 163 (7), 134 (36), 121 (100), 91 (21), 85 (16), 77 (20).

6-(3-Methoxyphenyl)-2,4-hexanedione (1g). Clear, colorless oil. ^{1}H NMR ($CDCl_3$, 500 MHz) – (enol tautomer) δ 15.46 (br. s, 1H), 7.23-7.19 (m, 1H), 6.81-6.72 (m, 3H), 5.49 (s, 1H), 3.80 (s, 3H), 2.92 (t, 2H, J = 7.9 Hz), 2.60 (t, 2H, J = 7.9 Hz), 2.05 (s, 3H). ^{13}C NMR ($CDCl_3$, 125 MHz) – δ 193.3, 191.0, 159.7, 142.3, 129.5, 120.6, 114.0, 111.5, 100.0, 55.1, 39.9, 31.5, 24.8. MS [*m/z*

(rel int)] 220 (M^+, 38), 202 (11), 162 (11), 135 (100), 121 (71), 105 (27), 91 (73), 85 (91), 77 (40).

6-(3-Chlorophenyl)-2,4-hexanedione (1H). ^{1}H NMR ($CDCl_3$, 500 MHz) – (enol tautomer) δ 15.41 (br. s, 1H), 7.22-7.17 (m, 3H), 7.09-7.06 (m, 1H), 5.47 (s, 1H), 2.92 (t, 2H, J = 8.1 Hz), 2.59 (t, 2H, J = 8.1 Hz), 2.05 (s, 3H). ^{13}C NMR ($CDCl_3$, 125 MHz) – δ 192.9, 190.8, 142.7, 134.2, 129.8, 129.7, 128.5, 128.4, 126.5, 126.4, 100.0, 58.0, 44.8, 39.7, 31.0, 28.9, 24.7. MS [m/z (rel int)] 224 (M^+, 38), 138 (33), 125 (42), 103 (35), 85 (100), 77 (28). IR (KBr) ν (cm_{-1}) 3644, 3167, 3064, 2939, 2670, 2365, 1843, 1592, 1438, 1330, 1262, 1142, 1038, 893, 785, 688. LC-HRMS calcd. for $C_{12}H_{14}ClO_2$ [M+H] 225.0677, found 225.0665.

6-(4-Chlorophenyl)-2,4-hexanedione (1i). White solid (mp 34–36 °C). ^{1}H NMR ($CDCl_3$, 500 MHz) – (enol tautomer) δ 15.42 (br. s, 1H), 7.26-7.23 (m, 2H), 7.14-7.10 (m, 2H), 5.46 (s, 1H), 2.91 (t, 2H, J = 7.9 Hz), 2.57 (t, 2H, J = 7.9 Hz), 2.04 (s, 3H). ^{13}C NMR ($CDCl_3$, 125 MHz) – δ 192.9, 190.9, 139.1, 132.0, 129.6, 128.6, 100.1, 39.8, 30.7, 24.8. MS [m/z (rel int)] 224 (M^+, 65), 138 (59), 125 (76), 103 (29), 85 (100), 77 (24). IR (KBr) ν (cm_{-1}) 3491, 2884, 2396, 2283, 1894, 1808, 1608, 1498, 1427, 1251, 1097, 1006, 940, 807. LC-HRMS calcd. for $C_{12}H_{13}ClNaO_2$ [M+Na] 247.0496, found 247.0487.

6-(2-Naphthyl)-2,4-hexanedione (1j). White solid (mp 57–58 °C). ^{1}H NMR ($CDCl_3$, 500 MHz) – (enol tautomer) δ 15.51 (br. s, 1H), 7.84-7.75 (m, 3H), 7.67-7.62 (s, 1H), 7.51-7.42 (m, 2H), 7.37-7.32 (m, 1H), 5.50 (s, 1H), 3.12 (t, 2H, J = 8.0 Hz), 2.70 (t, 2H, J = 8.0 Hz), 2.05 (s, 3H). ^{13}C NMR ($CDCl_3$, 125 MHz) – δ 193.1, 191.0, 138.2, 133.5, 132.1, 128.1, 127.6, 127.4, 126.9, 126.4, 126.0, 125.3, 100.0, 39.9, 31.6, 24.8. MS [m/z (rel int)] 240 (M^+, 55), 182 (12), 154 (65), 141 (100), 128 (35), 115 (62), 85 (40).

1-Acetyl-3,4-dihydro-2(2H)-naphthalenone (2a). Clear, light yellow oil. ^{1}H NMR ($CDCl_3$, 500 MHz) – (enol tautomer – enol:keto >95:5) δ 16.53 (br. s, 1H), 7.25-7.18 (m, 3H), 7.15-7.10 (m, 1H), 2.86 (t, 2H, J = 6.9 Hz), 2.56 (t, 2H, J = 6.9 Hz), 2.39 (s, 3H). ^{13}C NMR ($CDCl_3$, 125 MHz) – δ 200.0, 183.8, 135.4, 132.8, 127.4, 126.5, 126.4, 125.4, 110.9, 35.3, 27.8, 23.3. MS [m/z (rel int)] 188 (M^+, 26), 173 (23), 146 (21), 141 (21), 128 (24), 115 (100), 102 (10), 91 (24), 77 (16).

Methyl 3,4-dihydro-2(2H)-naphthalenone-1-carboxylate (2b). Clear, colorless oil. ^{1}H NMR ($CDCl_3$, 500 MHz) – (enol tautomer – enol:keto >90:10) δ 13.32 (br. s, 1H), 7.72-7.67 (m, 1H), 7.22-7.18 (m, 1H), 7.15-7.12 (m, 1H), 7.10-7.06 (m, 1H), 3.93 (s, 3H), 2.83 (t, 2H, J = 7.4 Hz), 2.55 (t, 2H, J = 7.4 Hz). ^{13}C NMR ($CDCl_3$, 125 MHz) – δ 178.4, 172.4, 133.2, 131.3, 127.2, 126.4, 125.8, 125.0, 99.9, 51.7, 29.5, 27.7. MS [m/z (rel int)] 204 (not observed), 189 (2), 146 (56), 117 (44), 104 (100), 91 (23), 78 (33).

1-Acetyl-3,4-dihydro-4-methyl-2(2H)-naphthalenone (2c). Clear, light yellow oil. ^{1}H NMR ($CDCl_3$, 500 MHz) – (enol tautomer – enol:keto >90:10) δ 16.56 (br. s, 1H), 7.26-7.15 (m, 4H), 3.07-3.00 (m, 1H), 2.66 (dd, 1H, J = 16.2, 5.1 Hz), 2.40 (s, 3H), 2.39 (dd, 1H, J = 7.6 Hz), 1.31 (d, 3H, J = 7.0 Hz). ^{13}C NMR ($CDCl_3$, 125 MHz) – δ 199.1, 183.6, 139.8, 132.1, 126.7, 126.2, 125.7, 110.8, 42.7, 32.4, 23.4, 18.9. MS [m/z (rel int)] 202 (M^+, 62), 187 (25), 141 (44), 128 (38),

115 (100), 105 (25), 91 (31). IR (KBr) ν (cm-1) 3089, 2958, 2549, 2411, 1595, 1408, 1271, 984, 759. LC-HRMS calcd. for $C_{13}H_{15}O_2$ [M+H] 203.1067, found 203.1060.

1-Acetyl-6,8-dimethoxy-3,4-dihydro-2(2H)-naphthalenone (2d) Clear, light yellow oil. ^{1}H NMR ($CDCl_3$, 500 MHz) – (enol tautomer – enol:keto 60:40) *δ* 15.56 (br. s, 1H), 6.41-6.32 (m, 2H), 3.80 (s, 3H), 3.77 (s, 3H), 3.19-2.37 (m, 4H), 2.00 (s, 3H). ^{13}C NMR ($CDCl_3$, 125 MHz) – *δ* 206.4, 203.3, 198.2, 183.8, 160.2, 158.9, 157.4, 155.7, 138.8, 138.7, 114.6, 114.0, 107.4, 104.4, 104.2, 96.9, 96.8, 62.6, 55.4, 55.3, 55.0, 37.7, 35.2, 29.6, 29.0, 28.5, 23.1. MS [*m/z* (rel int)] 248 (M^+, 32), 233 (6), 215 (21), 206 (100), 191 (30), 177 (34), 161 (13), 147 (11), 131 (11), 115 (11), 103 (11), 91 (15), 77 (13).

1-Acetyl-8-methoxy-3,4-dihydro-2(2H)-naphthalenone (2g) Single isomer existing in keto, *cis*-enol and *trans*-enol tautomers. Clear, light yellow oil. ^{1}H NMR ($CDCl_3$, 500 MHz) – (enol tautomers – enol:keto 70:30) *δ* 16.32 (br. s, 1H), 15.75 (br. s, 1H), 7.26-7.09 (m, 2H), 6.88-6.76 (m, 4H), 3.82 (s, 6H), 3.24-2.42 (m, 8H), 2.06 (s, 6H). ^{13}C NMR ($CDCl_3$, 125 MHz) – *δ* 206.2, 202.9, 197.7, 185.5, 182.0, 157.4, 156.4, 154.6, 138.1, 137.8, 137.0, 128.6, 127.5, 126.8, 125.3, 121.8, 121.7, 120.5, 119.6, 113.2, 111.4, 110.5, 109.6, 108.5, 107.6, 63.0, 55.4, 55.3, 55.0, 37.7, 35.5, 34.9, 29.9, 28.5, 28.2, 28.1, 23.4, 23.0. MS [*m/z* (rel int)] 218 (M^+, 31), 203 (14), 185 (17), 176 (100), 161 (48), 131 (31), 115 (43), 103 (28), 91 (20), 77 (20). IR (KBr) ν (cm-1) 3635, 2948, 2823, 1712, 1597, 1461, 1425, 1260, 1167, 1086, 969. LC-HRMS calcd. for $C_{13}H_{15}O_3$ [M+H] 219.1016, found 219.1006.

4-Acetyl-1,2-dihydro-3(2H)-phenanthrenone (2j). Clear, light yellow oil. ^{1}H NMR ($CDCl_3$, 500 MHz) – (enol tautomer – enol:keto >90:10) *δ* 15.51 (br. s, 1H), 7.91-7.81 (m, 1H), 7.71 (d, 1H, *J* = 8.3 Hz), 7.64 (d, 1H, *J* = 8.2 Hz), 7.50-7.42 (m, 2H), 7.39 (d, 1H, *J* = 8.2 Hz), 3.18 (td, 1H, *J* = 15.0, 4.3 Hz), 2.93-2.85 (m, 1H), 2.71-2.62 (m, 1H), 2.51 (td, 1H, *J* = 15.6, 5.4 Hz), 1.91 (s, 3H). ^{13}C NMR ($CDCl_3$, 125 MHz) – *δ* 206.4, 202.8, 201.7, 179.7, 135.4, 134.6, 133.1, 133.0, 131.3, 130.0, 129.4, 129.2, 128.6, 127.3, 126.9, 126.5, 125.8, 125.7, 125.7, 125.4, 125.0, 122.2, 110.1, 65.8, 37.3, 36.2, 29.2, 28.8, 28.7, 23.1. MS [*m/z* (rel int)] 238 (M^+, 23), 196 (63), 178 (32), 165 (100), 152 (47), 139 (25), 115 (15). IR (KBr) ν (cm-1) 3420, 3048, 2953, 2254, 1712, 1601, 1402, 1248, 1026, 915, 818, 739. LC-HRMS calcd. for $C_{16}H_{15}O_2$ [M+H] 239.1067, found 239.1062.

Computational results

Table S1: Absolute energies (Hartree), zero-point vibrational energies (ZPVE, kcal/mol) and dipole moments (Debye) for structures related to the anti-isomer of **1x'** as calculated at the UB3LYP/6-31G(d) level of theory.

	E, Hartrees	ZPVE kcal/mol	Dipole, Debye
**1a'**	-615.50932	137.8	2.21
**TS1a'**	-615.50561	137.9	2.03
**2a'**	-615.50894	138.1	2.11
**TS2a'**	-615.48538	138.6	3.97
**3a'**	-615.49706	138.9	3.97
**1g'**	-730.03219	158.4	3.55
**TS1g'**	-730.02834	158.5	3.14
**2g'**	-730.03148	158.6	2.53
**TS2g'**	-730.01461	159.4	4.11
**3g'**	-730.02556	160.0	4.24
**1g''**	-730.03219	158.4	3.55
**TS1g''**	-730.02849	158.5	2.99
**2g''**	-730.03170	158.7	2.38
**TS2g''**	-730.01137	159.3	4.81
**3g''**	-730.02062	159.6	4.29
**1f'**	-730.03168	158.4	3.12
**TS1f'**	-730.02792	158.5	3.38
**2f'**	-730.03133	158.7	3.13
**TS2f'**	-730.00653	159.0	5.04
**3f'**	-730.01725	159.4	5.14
**1h'**	-1075.105489	131.8	1.98
**TS1h'**	-1075.10184	131.9	3.11
**2h'**	-1075.10524	132.1	3.55
**TS2h'**	-1075.07966	132.5	4.79
**3h'**	-1075.09142	133.2	4.97
**1j'**	-769.1534616	167.3	2.22
**TS1j'**	-769.1496332	167.3	2.07
**2j'**	-769.1531454	167.5	2.11
**TS2j'**	-769.1346359	168.0	4.05
**3j'**	-769.1495992	168.8	3.83

**1j"**	-769.1534616	167.3	2.22
**TS1j"**	-769.1499887	167.4	1.96
**2j"**	-769.1529335	167.6	2.02
**TS2j"**	-769.132151	168.0	4.11
**3j"**	-769.1455974	168.5	3.94

Table S2: Absolute energies (Hartree), relative energies (R.E. kcal/mol), zero-point vibrational energies (ZPVE, kcal/mol), low frequencies, and dipole moments (Debye) for structures related to the syn isomer (**1a**) as calculated at the UB3LYP/6-31G(d) level of theory.

**Syn**	E, Hartrees	ZPVE (kcal/mol)	R.E.	R. E. + ZPVE (kcal/mol)	low freq. cm^{-1}	Dipole, D
**1a**	-615.50152	137.43	0.00	0.0	22.4	4.80
**Ts1a**	-615.49791	137.47	2.26	2.3	49.7i	4.77
**2a**	-615.50123	137.66	0.18	0.4	15.2	4.61
**TS2a**	-615.48188	138.18	12.32	13.1	485.3i	5.47
**3a**	-615.50043	138.91	0.68	2.2	47.1	3.62

Calculated transition structures

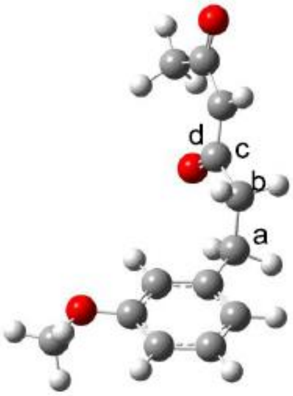

dihedral angle abcd = 4.2 deg

TS1g'

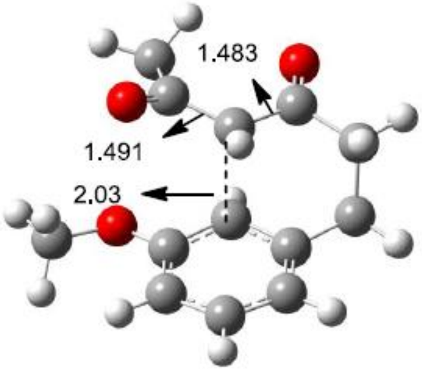

TS2g'

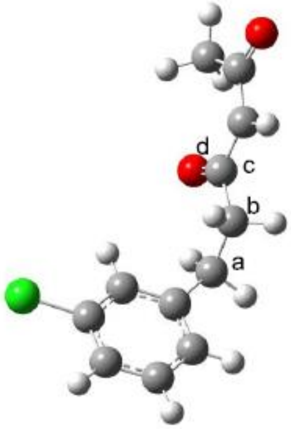

dihedral angle abcd = 6.3 deg

TS1h'

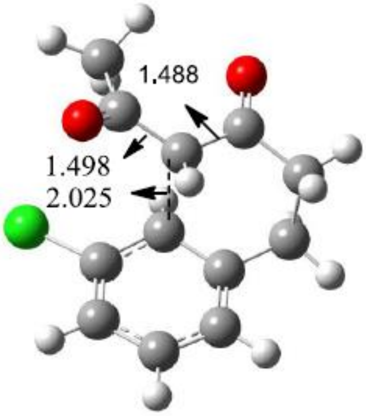

TS2h'

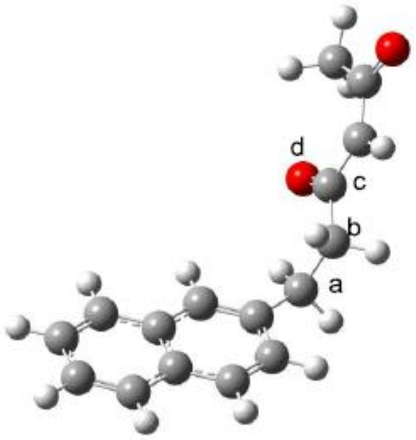

dihedral angle abcd = 1.1 deg

TS1j'

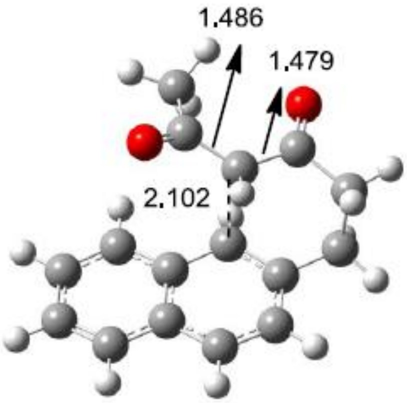

TS2j'

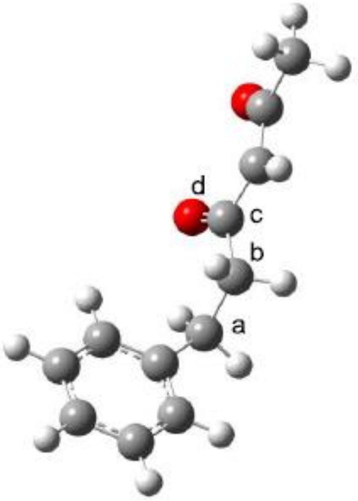

dihedral angle abcd = 6.4 deg

TS1a

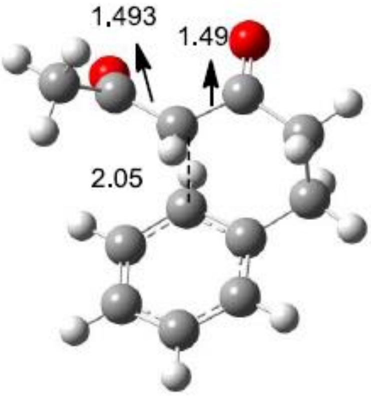

TS2a

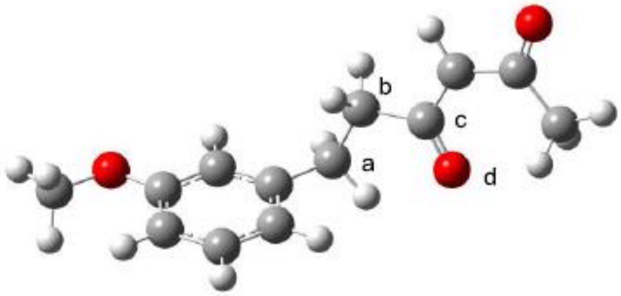

dihedral angle abcd = 1.1 deg

TS1g"

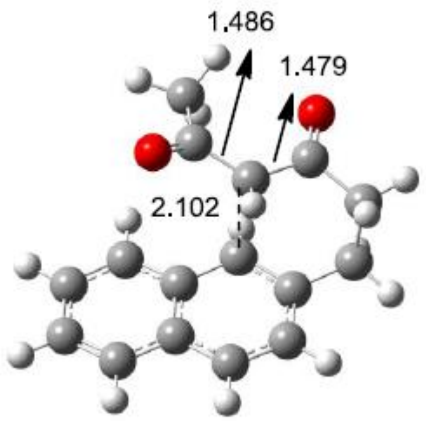

TS2g"

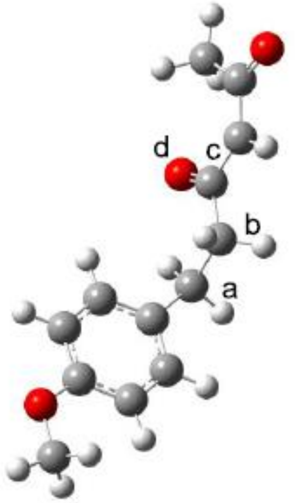

dihedral angle abcd = 4.4 deg

TS1f'

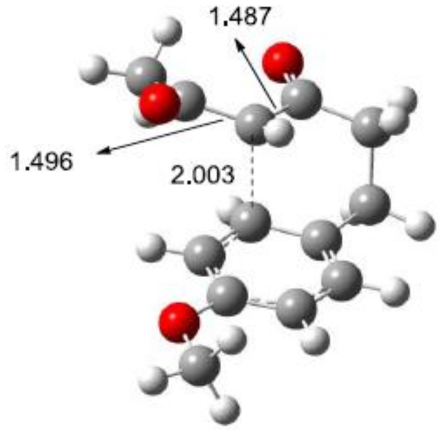

TS2f'

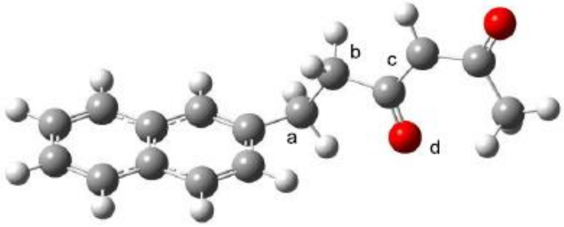

dihedral angle abcd = -9.3 deg

TS1j”

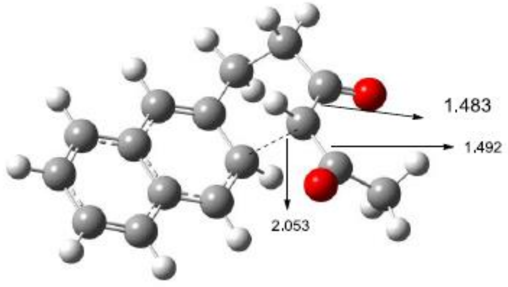

TS2j”

Cartesian coordinates

1a’

C	-2.712128	-0.760050	-0.000371
C	2.890457	0.154494	-1.203099
C	2.220285	0.413574	0.000221
C	0.796160	0.927563	0.000411
C	-0.232856	-0.213092	-0.001006
H	-2.404520	-1.803462	-0.000509
H	0.618851	1.561599	0.876219
H	0.619225	1.563408	-0.874161
H	-0.084516	-0.865142	-0.874447
H	-0.084396	-0.867581	0.870588
H	2.390507	0.360236	-2.147620
C	4.189859	-0.354207	-1.206130
C	4.843952	-0.611754	-0.000046
H	5.857274	-1.004263	-0.000139
C	4.190031	-0.354378	1.206163
H	4.693601	-0.544213	2.150580
C	2.890605	0.154275	1.203398
H	2.390771	0.359824	2.148019
C	-4.784399	0.810419	-0.000086
H	-4.458905	1.383905	-0.873760
H	-5.871003	0.702924	-0.000411
H	-4.459474	1.383686	0.873951
C	-1.677982	0.272816	-0.000310
C	-4.166451	-0.569922	0.000056
O	-4.865805	-1.587741	0.000615
O	-1.957433	1.474560	0.000275
H	4.693288	-0.543898	-2.150651

TS1a’

C	-2.681741	-0.568315	-0.548577
C	2.277297	1.082810	0.077486
C	2.155792	-0.265774	0.443895
C	0.872852	-0.775948	1.067156
C	-0.257152	-1.029415	0.040071
H	-2.547621	-1.310332	-1.332518
H	1.075301	-1.700467	1.618273
H	0.502548	-0.047405	1.796108
H	0.065523	-0.743817	-0.971393
H	-0.503942	-2.097199	-0.032859
H	1.451627	1.763787	0.274118
C	3.442179	1.559686	-0.523320
C	4.510914	0.693944	-0.766971
H	5.420466	1.064867	-1.231829
C	4.404587	-0.648914	-0.403368
H	5.232505	-1.329911	-0.583547
C	3.235735	-1.122141	0.196576
H	3.162634	-2.169884	0.481032
C	-4.405390	1.064238	0.513132

H	-3.759898	1.944129	0.427657
H	-5.448690	1.342922	0.351667
H	-4.266148	0.686192	1.530892
C	-1.549772	-0.263005	0.325033
C	-4.026169	0.016420	-0.509206
O	-4.836889	-0.384957	-1.349898
O	-1.635298	0.575740	1.224459
H	3.518402	2.609340	-0.795528

2a’

C	-2.376189	0.535465	0.605187
C	1.947333	-0.692954	-0.923375
C	1.954285	0.584081	-0.345758
C	1.038909	1.683109	-0.850410
C	-0.274191	1.802577	-0.054944
H	-2.398368	1.177360	1.482819
H	1.559495	2.646096	-0.788459
H	0.793794	1.515554	-1.903734
H	-0.074954	1.832566	1.023956
H	-0.769019	2.757340	-0.293732
H	1.276726	-0.894804	-1.753430
C	2.782607	-1.700429	-0.437253
C	3.638692	-1.448490	0.635869
H	4.290611	-2.232405	1.012173
C	3.655852	-0.180097	1.219329
H	4.323681	0.029158	2.051126
C	2.821173	0.824859	0.729734
H	2.848412	1.814172	1.183196
C	-3.624502	-1.404265	-0.596729
H	-2.743015	-2.051220	-0.649040
H	-4.523652	-2.001372	-0.431695
H	-3.680219	-0.891731	-1.562249
C	-1.287883	0.700859	-0.353942
C	-3.496076	-0.409735	0.535047
O	-4.332054	-0.356580	1.442238
O	-1.211562	0.008209	-1.373087
H	2.765377	-2.683623	-0.900670

TS2a’

C	0.897635	-0.055638	-0.567763
C	-0.578507	-0.232383	0.799414
C	-1.462791	0.834936	0.363976
C	-0.933657	2.242519	0.400446
C	0.253666	2.401704	-0.599774
H	0.272956	-0.196487	-1.447116
H	-1.721289	2.961118	0.150359
H	-0.570092	2.493805	1.405779
H	-0.125969	2.289836	-1.622548
H	0.710054	3.389599	-0.495651
H	0.089955	-0.001200	1.629580
C	-1.077313	-1.583831	0.742588

C	-2.284024	-1.865492	0.134951
H	-2.626674	-2.893772	0.061859
C	-3.086215	-0.823955	-0.363988
H	-4.039228	-1.048256	-0.834111
C	-2.666878	0.511797	-0.238253
H	-3.303208	1.309565	-0.615241
C	2.922340	-1.165463	0.653386
H	2.560962	-0.812027	1.626541
H	3.359151	-2.161603	0.753221
H	3.681219	-0.442850	0.339660
C	1.309889	1.346306	-0.299354
C	1.802223	-1.228019	-0.364745
O	1.582559	-2.251847	-1.001822
O	2.367795	1.652228	0.243570
H	-0.468887	-2.385646	1.148380

3a'

C	0.790780	-0.083526	-0.427461
C	-0.474907	-0.305652	0.515793
C	-1.480089	0.798984	0.281623
C	-0.957146	2.199181	0.435471
C	0.275914	2.437392	-0.475545
H	0.433148	-0.187664	-1.459339
H	-1.736288	2.934591	0.207429
H	-0.649377	2.375143	1.478453
H	-0.047114	2.413428	-1.525213
H	0.744483	3.405874	-0.279608
H	-0.084328	-0.204997	1.546973
C	-1.051392	-1.683931	0.345224
C	-2.350272	-1.892890	-0.022161
H	-2.714579	-2.910272	-0.142630
C	-3.235265	-0.809115	-0.248542
H	-4.263666	-0.992920	-0.542077
C	-2.768452	0.518141	-0.090500
H	-3.456205	1.343614	-0.265993
C	2.577614	-1.269475	1.089625
H	1.866994	-1.321691	1.923426
H	3.209520	-2.159887	1.088858
H	3.190568	-0.374661	1.239639
C	1.314624	1.348721	-0.263295
C	1.872342	-1.157798	-0.246592
O	2.133057	-1.902425	-1.172615
O	2.468589	1.603512	0.029808
H	-0.388810	-2.529432	0.510694

1g'

C	-3.453958	-0.401950	0.700954
C	2.212266	-0.384636	-0.576932
C	1.379138	0.709503	-0.346692
C	-0.052838	0.700018	-0.839026
C	-1.022801	0.141733	0.213005

H	-3.101797	-0.753497	1.668253
H	-0.369005	1.713862	-1.108536
H	-0.138546	0.101695	-1.752572
H	-0.735862	-0.881580	0.496483
H	-0.958482	0.723486	1.144512
H	1.861533	-1.244604	-1.141449
C	3.529293	-0.404351	-0.095607
C	4.024200	0.686511	0.627484
H	5.039881	0.695828	1.005709
C	3.187954	1.783792	0.855647
H	3.573397	2.634840	1.411336
C	1.881142	1.802921	0.378135
H	1.247707	2.668327	0.557665
O	4.243928	-1.531178	-0.388842
C	5.587350	-1.609535	0.058951
H	5.652670	-1.562191	1.154234
H	5.960743	-2.576940	-0.282125
H	6.204896	-0.810503	-0.372518
C	-5.578079	-0.092878	-0.766542
H	-5.170568	-0.634803	-1.625853
H	-6.649768	-0.280477	-0.673822
H	-5.393670	0.967700	-0.964814
C	-2.475912	0.127819	-0.247428
C	-4.903259	-0.527679	0.515250
O	-5.550508	-1.004738	1.452679
O	-2.807182	0.542322	-1.361298

TS1g’

C	-3.318897	-0.239366	-0.706410
C	1.762334	0.206695	0.444906
C	1.406220	-1.141587	0.384956
C	0.022556	-1.578691	0.821118
C	-1.071036	-1.318279	-0.242468
H	-3.251136	-0.711882	-1.683815
H	0.039224	-2.644673	1.071432
H	-0.263426	-1.043431	1.732535
H	-0.643997	-0.824685	-1.126825
H	-1.497535	-2.257586	-0.620376
H	1.069308	0.948748	0.832546
C	3.025562	0.638682	0.018428
C	3.951668	-0.288602	-0.474384
H	4.935481	0.021973	-0.806676
C	3.593882	-1.638352	-0.530126
H	4.313259	-2.360413	-0.908149
C	2.338231	-2.067597	-0.108256
H	2.078994	-3.122363	-0.155052
O	3.255693	1.981123	0.128204
C	4.522025	2.479980	-0.269385
H	4.714182	2.295269	-1.334888
H	4.489134	3.556543	-0.091556
H	5.334964	2.039870	0.323476

C	-4.815859	1.277392	0.784115
H	-4.026821	2.006087	0.995678
H	-5.781656	1.779693	0.699342
H	-4.820723	0.584212	1.631171
C	-2.226341	-0.442309	0.244075
C	-4.541536	0.546318	-0.511025
O	-5.339973	0.584803	-1.452542
O	-2.238483	0.074940	1.362825

2g’

C	2.965310	-0.362875	0.544165
C	-1.530002	-0.081929	-0.748644
C	-1.216682	-1.386576	-0.368904
C	-0.090451	-2.145208	-1.044755
C	1.249680	-2.045136	-0.292548
H	3.159270	-1.068251	1.348778
H	-0.360749	-3.205262	-1.116967
H	0.054957	-1.776917	-2.064708
H	1.116532	-2.270086	0.773058
H	1.948303	-2.805821	-0.675485
H	-0.978683	0.410784	-1.542798
C	-2.557782	0.626004	-0.107683
C	-3.281779	0.026075	0.928224
H	-4.081241	0.554177	1.434850
C	-2.965085	-1.282423	1.306864
H	-3.531119	-1.752730	2.106984
C	-1.948023	-1.986502	0.670609
H	-1.723180	-3.007390	0.970870
O	-2.772688	1.893610	-0.570821
C	-3.804320	2.660488	0.026958
H	-3.618674	2.826153	1.096779
H	-3.801994	3.621387	-0.491155
H	-4.786515	2.184792	-0.097164
C	3.657761	1.952236	-0.418167
H	2.637446	2.348722	-0.421843
H	4.370896	2.741416	-0.171234
H	3.839221	1.578916	-1.430946
C	1.948985	-0.696572	-0.448940
C	3.800874	0.842287	0.598567
O	4.628571	0.909659	1.512421
O	1.692194	0.059344	-1.390787

TS2g’

C	-0.750311	-0.760763	0.654704
C	-0.002041	0.566637	-0.686337
C	-0.815448	1.722230	-0.376942
C	-2.292799	1.600776	-0.631454
C	-2.916685	0.504433	0.291788
H	-0.600450	-0.138937	1.534554
H	-2.799065	2.555801	-0.455674
H	-2.483027	1.316407	-1.674802

H	-2.881455	0.855745	1.330072
H	-3.958354	0.324157	0.013201
H	-0.279066	-0.031877	-1.553154
C	1.417688	0.649501	-0.438327
C	1.946304	1.704818	0.292784
H	3.000879	1.738340	0.540647
C	1.106624	2.760052	0.678791
H	1.525168	3.590772	1.239417
C	-0.257097	2.768779	0.335823
H	-0.880130	3.608095	0.634905
O	2.116474	-0.391801	-0.943436
C	3.464789	-0.579054	-0.516321
H	3.507522	-0.691373	0.571864
H	3.799701	-1.500195	-0.994484
H	4.102607	0.252454	-0.839917
C	-0.001521	-3.059179	-0.320367
H	0.034163	-2.669231	-1.344516
H	0.803001	-3.783203	-0.173071
H	-0.979594	-3.535241	-0.209463
C	-2.142018	-0.801211	0.142900
C	0.164560	-1.937877	0.681405
O	1.073249	-1.949113	1.508562
O	-2.620245	-1.749908	-0.473908

3g'

C	-0.770005	-0.500961	0.454827
C	0.106370	0.442258	-0.491982
C	-0.356157	1.871418	-0.324295
C	-1.809240	2.110819	-0.614857
C	-2.698793	1.210192	0.282312
H	-0.563178	-0.181065	1.483895
H	-2.070729	3.163103	-0.459566
H	-2.035126	1.877177	-1.667103
H	-2.595733	1.539378	1.325223
H	-3.754815	1.269348	0.003530
H	-0.100830	0.104792	-1.522926
C	1.580197	0.274028	-0.232569
C	2.369847	1.276861	0.265950
H	3.419586	1.099125	0.472642
C	1.830835	2.568604	0.487783
H	2.466553	3.354328	0.882163
C	0.480154	2.837344	0.172687
H	0.092615	3.843374	0.322026
O	1.995224	-0.980479	-0.544790
C	3.330020	-1.358237	-0.226192
H	3.519002	-1.255295	0.848757
H	3.421952	-2.405519	-0.516353
H	4.054200	-0.754224	-0.786855
C	-0.588623	-2.754288	-0.892800
H	-0.158705	-2.212609	-1.741110
H	-0.111672	-3.732562	-0.801082

H	-1.659638	-2.882948	-1.080796
C	-2.261441	-0.242276	0.189824
C	-0.415055	-1.996922	0.406565
O	-0.038520	-2.544575	1.425931
O	-3.045721	-1.132376	-0.084385

TS1g''

C	3.444993	-0.755744	-0.467324
C	-2.437266	-0.630472	0.624077
C	-1.281400	0.147618	0.625855
C	-0.017341	-0.340922	1.303023
C	1.035834	-0.895730	0.314052
H	3.209291	-1.620238	-1.083953
H	0.445723	0.483504	1.855517
H	-0.269475	-1.113486	2.036693
H	1.203488	-1.970664	0.466177
H	0.680141	-0.808845	-0.722467
H	-2.464831	-1.591678	1.130143
C	-3.601112	-0.194559	-0.027484
C	-3.610826	1.037867	-0.688387
H	-4.498496	1.396847	-1.196298
C	-2.449650	1.818415	-0.683277
H	-2.458235	2.780177	-1.189962
C	-1.296101	1.387440	-0.036878
H	-0.405176	2.010569	-0.032945
O	-4.666779	-1.047164	0.043243
C	-5.872690	-0.666769	-0.598353
H	-5.732684	-0.540097	-1.680278
H	-6.577223	-1.481279	-0.419618
H	-6.279794	0.262526	-0.177864
C	5.346566	0.895685	0.180286
H	5.241177	0.728948	1.256995
H	6.396232	1.049061	-0.078427
H	4.764871	1.795496	-0.043482
C	2.392901	-0.193981	0.378817
C	4.828836	-0.289925	-0.603007
O	5.555069	-0.909534	-1.386599
O	2.592777	0.794901	1.087276

2g''

C	-2.949122	-0.216579	-0.928813
C	2.140011	0.729496	-0.617416
C	1.089178	1.127625	0.210245
C	0.071550	2.134286	-0.292041
C	-1.075125	1.494946	-1.097428
H	-2.803725	-0.373878	-1.994970
H	-0.359241	2.685231	0.549649
H	0.571408	2.865592	-0.937588
H	-1.630181	2.278174	-1.637745
H	-0.680350	0.821835	-1.868737
H	2.259118	1.151051	-1.612353

C	3.080056	-0.217454	-0.188213
C	2.972336	-0.775637	1.090439
H	3.689586	-1.505962	1.446919
C	1.920268	-0.373300	1.918613
H	1.838305	-0.803418	2.913633
C	0.984689	0.565573	1.493208
H	0.168393	0.863281	2.143400
O	4.061728	-0.522241	-1.089756
C	5.046812	-1.469696	-0.712491
H	4.603212	-2.450906	-0.496528
H	5.719449	-1.559520	-1.567652
H	5.617908	-1.133428	0.163273
C	-4.357162	-0.975754	1.122864
H	-4.639078	0.038706	1.421857
H	-5.176847	-1.670262	1.318087
H	-3.484660	-1.237455	1.729986
C	-2.098486	0.751155	-0.242761
C	-4.024421	-1.030906	-0.351322
O	-4.648484	-1.764857	-1.123698
O	-2.225344	0.983152	0.963388

TS2g’’

C	1.462826	-0.268510	0.576156
C	-1.730129	1.310607	-0.074531
C	-0.431476	1.290081	-0.526927
C	0.472557	2.487659	-0.414728
C	1.532486	2.262607	0.709278
H	0.723428	-0.249256	1.373978
H	1.007216	2.656659	-1.358975
H	-0.104342	3.393157	-0.199322
H	2.253914	3.083959	0.718816
H	1.019491	2.225692	1.677767
H	-2.178576	2.230456	0.291110
C	-2.528247	0.141719	-0.070415
C	-2.000746	-1.072439	-0.551721
H	-2.610669	-1.967815	-0.586065
C	-0.701122	-1.116514	-1.016431
H	-0.308157	-2.048538	-1.408782
C	0.171502	0.026697	-0.930789
H	0.956758	0.085341	-1.685132
O	-3.787991	0.303752	0.405472
C	-4.650152	-0.827262	0.460179
H	-4.232045	-1.617053	1.096206
H	-5.584038	-0.466884	0.893949
H	-4.845494	-1.229878	-0.541482
C	3.250120	-1.870335	-0.450028
H	4.123799	-1.409007	0.019541
H	3.400038	-2.946126	-0.566627
H	3.157756	-1.392230	-1.432552
C	2.279807	0.961825	0.440867
C	2.012006	-1.643447	0.393975

O	1.406204	-2.587791	0.891169
O	3.429830	0.978463	0.008142

3g’’

C	-1.423139	-0.228779	-0.431832
C	1.825640	1.257683	0.116731
C	0.493228	1.213510	0.399026
C	-0.370120	2.442224	0.447828
C	-1.545321	2.335890	-0.560897
H	-0.959685	-0.262850	-1.425239
H	-0.797282	2.564798	1.455689
H	0.218018	3.342527	0.240108
H	-2.253429	3.161556	-0.447211
H	-1.140699	2.361380	-1.581727
H	2.319698	2.210210	-0.060532
C	2.626468	0.083068	0.049163
C	2.033422	-1.179968	0.278215
H	2.634588	-2.082441	0.237751
C	0.696448	-1.285444	0.561461
H	0.265305	-2.267561	0.733483
H	-0.224132	-0.099554	0.619072
H	-0.719709	-0.068615	1.608595
O	3.941708	0.293648	-0.239179
C	4.804382	-0.830588	-0.328306
H	4.487034	-1.519311	-1.121959
H	5.791211	-0.431727	-0.569472
H	4.856064	-1.374727	0.623789
C	-2.998875	-1.782268	0.982734
H	-3.857719	-1.103131	0.991368
H	-3.340591	-2.819232	1.000694
H	-2.410311	-1.569347	1.883395
C	-2.295804	1.028745	-0.365404
C	-2.201983	-1.541043	-0.283056
O	-2.149474	-2.370195	-1.172739
O	-3.493958	0.998750	-0.147725

1f’

C	-3.576997	0.823682	0.110021
C	1.901434	-0.614433	1.258571
C	1.271157	-0.671996	0.004614
C	-0.183175	-1.071865	-0.121688
C	-1.138241	0.117747	0.058205
H	-3.203897	1.825654	0.310435
H	-0.369025	-1.528349	-1.100307
H	-0.434034	-1.839412	0.619462
H	-0.979211	0.597016	1.035637
H	-0.918700	0.902540	-0.680970
H	1.344011	-0.892157	2.151034
C	3.227012	-0.220307	1.387004
C	3.969678	0.131410	0.250337
C	3.363660	0.079338	-1.009303

H	3.914339	0.338114	-1.906584
C	2.027968	-0.320520	-1.115719
H	1.572888	-0.363592	-2.103343
C	-5.742808	-0.575368	-0.223747
H	-5.481830	-1.320765	0.534111
H	-6.819960	-0.396431	-0.224712
H	-5.426957	-0.998260	-1.182668
C	-2.610422	-0.259582	-0.060944
C	-5.039531	0.736352	0.044904
O	-5.673454	1.781857	0.219089
O	-2.965933	-1.418760	-0.290110
O	5.265104	0.501496	0.478334
C	6.065278	0.858317	-0.636499
H	5.656306	1.729173	-1.166275
H	7.047023	1.111901	-0.232141
H	6.169366	0.024266	-1.343397
H	3.713877	-0.183082	2.356682

TS1f’

C	-3.519489	0.148183	0.819972
C	1.417895	-0.618104	-0.772701
C	1.211764	0.739625	-0.472376
C	-0.137449	1.385495	-0.708702
C	-1.190107	1.047634	0.374890
H	-3.340064	0.428055	1.855666
H	-0.016498	2.472495	-0.768733
H	-0.538895	1.060914	-1.674735
H	-0.772487	0.355174	1.119651
H	-1.478429	1.938989	0.948990
H	0.605531	-1.197486	-1.206712
C	2.639350	-1.234420	-0.536760
C	3.703812	-0.501824	0.010163
C	3.522856	0.851346	0.312025
H	4.328782	1.444439	0.729395
C	2.283170	1.452625	0.068407
H	2.158573	2.507256	0.305586
C	-5.265995	-0.914858	-0.786946
H	-4.574088	-1.663073	-1.186412
H	-6.272683	-1.331192	-0.712715
H	-5.251151	-0.081509	-1.496547
C	-2.468411	0.404511	-0.164103
C	-4.833583	-0.460160	0.589064
O	-5.573725	-0.586038	1.569787
O	-2.608127	0.104588	-1.351671
O	4.865175	-1.196258	0.202675
C	5.977396	-0.502228	0.741972
H	5.761703	-0.107782	1.744170
H	6.784166	-1.234514	0.809695
H	6.292807	0.324668	0.091465
H	2.798886	-2.281725	-0.774505

2f'

C	3.079926	0.013990	-0.826905
C	-1.128762	0.283748	1.240622
C	-1.006336	1.299270	0.277300
C	0.142689	2.287631	0.305298
C	1.350391	1.858554	-0.549134
H	3.085720	0.277410	-1.881976
H	-0.204180	3.259283	-0.066151
H	0.483656	2.440706	1.334067
H	1.029373	1.562059	-1.555826
H	2.029811	2.714196	-0.690877
H	-0.387468	0.210485	2.030910
C	-2.174730	-0.629730	1.196485
C	-3.136849	-0.554385	0.179254
C	-3.035030	0.449953	-0.789599
H	-3.767964	0.538899	-1.583461
C	-1.976337	1.360541	-0.727182
H	-1.916956	2.142315	-1.482405
C	4.137316	-1.581683	0.934487
H	3.175693	-1.961728	1.293787
H	4.887386	-2.375112	0.947849
H	4.420388	-0.779134	1.622828
C	2.186107	0.740764	0.069794
C	4.016129	-1.061312	-0.480105
O	4.703633	-1.522985	-1.396067
O	2.134650	0.486862	1.277209
O	-4.126932	-1.495328	0.225010
C	-5.129776	-1.460677	-0.776340
H	-4.705873	-1.604215	-1.779421
H	-5.807647	-2.286123	-0.550894
H	-5.690139	-0.516260	-0.755632
H	-2.271039	-1.411157	1.944133

TS2f'

C	-1.328061	0.429766	-0.561956
C	-0.069726	-0.266839	0.831983
C	0.057035	-1.646440	0.383724
C	-1.179320	-2.503543	0.369995
C	-2.222008	-1.946003	-0.646751
H	-0.714867	0.190849	-1.428340
H	-0.930909	-3.537727	0.108470
H	-1.646853	-2.524073	1.363642
H	-1.825171	-2.062324	-1.662621
H	-3.165448	-2.492935	-0.568923
H	-0.772058	-0.085251	1.646587
C	1.109396	0.553160	0.838349
C	2.287978	0.113181	0.251042
C	2.365775	-1.193899	-0.268995
H	3.279779	-1.560530	-0.720688
C	1.249303	-2.048270	-0.183824
H	1.338037	-3.058786	-0.576823

C	-2.366740	2.497142	0.649350
H	-2.268636	2.004708	1.624463
H	-2.163213	3.565827	0.746455
H	-3.400696	2.328928	0.334574
C	-2.485693	-0.477391	-0.341017
C	-1.407900	1.910651	-0.366332
O	-0.648809	2.626016	-1.008556
O	-3.544263	-0.118989	0.166785
O	3.325508	0.997213	0.257843
C	4.564082	0.596100	-0.306865
H	4.462966	0.355844	-1.373315
H	5.232410	1.451365	-0.194068
H	4.990174	-0.267769	0.220725
H	1.080276	1.552258	1.259066

3f’

C	1.266120	-0.351839	-0.422368
C	0.114779	0.144850	0.561990
C	-0.157892	1.607600	0.308902
C	1.029032	2.525570	0.393635
C	2.168156	2.054839	-0.548290
H	0.867255	-0.263732	-1.440370
H	0.744200	3.554345	0.147686
H	1.422411	2.542665	1.422216
H	1.845097	2.184209	-1.590154
H	3.085707	2.629865	-0.395572
H	0.546148	0.047238	1.577161
C	-1.103120	-0.722342	0.465882
C	-2.343673	-0.225891	0.134437
C	-2.525218	1.155001	-0.118089
H	-3.495652	1.555373	-0.382528
C	-1.413347	2.032324	-0.021628
H	-1.578114	3.090522	-0.216954
C	2.168466	-2.293943	1.099128
H	1.516828	-1.989528	1.927033
H	2.265644	-3.381425	1.090636
H	3.147608	-1.832536	1.265409
C	2.475812	0.584945	-0.315245
C	1.631541	-1.831989	-0.240456
O	1.481064	-2.601245	-1.170273
O	3.595985	0.192136	-0.044308
O	-3.358274	-1.143039	0.075481
C	-4.658815	-0.698489	-0.273344
H	-4.682244	-0.253838	-1.277224
H	-5.289673	-1.589320	-0.262718
H	-5.050226	0.027602	0.451863
H	-1.006234	-1.788072	0.648992

1h’

C	3.392136	-0.325544	-0.719566
C	-2.276893	-0.403591	0.458176

C	-1.450819	0.723178	0.354476
C	-0.022720	0.672664	0.854517
C	0.954628	0.186285	-0.226356
H	3.053396	-0.602562	-1.715432
H	0.293342	1.663507	1.198223
H	0.053695	0.011712	1.724712
H	0.668456	-0.813237	-0.585186
H	0.902514	0.833815	-1.113987
H	-1.910621	-1.311447	0.928340
C	-3.579002	-0.359868	-0.035705
C	-4.092250	0.788129	-0.636516
H	-5.110264	0.801703	-1.010367
C	-3.269567	1.910718	-0.736373
H	-3.655845	2.816364	-1.195919
C	-1.963875	1.880014	-0.246749
H	-1.337340	2.765354	-0.324938
C	5.495636	-0.135001	0.797631
H	5.074308	-0.739982	1.606774
H	6.568118	-0.318445	0.706552
H	5.310842	0.907984	1.073720
C	2.402421	0.134680	0.251290
C	4.839041	-0.467954	-0.523238
O	5.497992	-0.872043	-1.486077
O	2.715940	0.465889	1.397602
Cl	-4.599675	-1.787517	0.109751

TS1h’

C	-3.262147	-0.349118	-0.676616
C	1.842449	0.265189	0.362105
C	1.511818	-1.097308	0.392638
C	0.134834	-1.527235	0.854282
C	-0.967265	-1.327981	-0.213989
H	-3.205491	-0.876533	-1.626188
H	0.164386	-2.579430	1.155681
H	-0.152124	-0.951458	1.740531
H	-0.556669	-0.849131	-1.114300
H	-1.367940	-2.289285	-0.562203
H	1.125938	1.009286	0.697355
C	3.097117	0.665299	-0.090044
C	4.049450	-0.260750	-0.514622
H	5.022033	0.073409	-0.859281
C	3.720545	-1.615471	-0.478347
H	4.452017	-2.351334	-0.801107
C	2.465413	-2.030632	-0.030495
H	2.225098	-3.090686	-0.005389
C	-4.764626	1.212676	0.761793
H	-3.990136	1.972012	0.909711
H	-5.744979	1.684937	0.672397
H	-4.731470	0.571192	1.648122
C	-2.140921	-0.464106	0.253324
C	-4.502520	0.412676	-0.494201

O	-5.324795	0.370751	-1.414395
O	-2.138246	0.115495	1.341233
Cl	3.491693	2.381625	-0.119117

2h′

C	2.928974	-0.532887	0.474254
C	-1.596159	0.073910	-0.636414
C	-1.359891	-1.287457	-0.402000
C	-0.268503	-2.027586	-1.151613
C	1.083694	-2.039102	-0.414792
H	3.134666	-1.328860	1.186167
H	-0.580697	-3.066811	-1.305822
H	-0.122409	-1.582996	-2.140681
H	0.955386	-2.338224	0.633345
H	1.743054	-2.798420	-0.863595
H	-1.000943	0.610720	-1.366554
C	-2.590766	0.736156	0.080682
C	-3.366300	0.079919	1.034580
H	-4.136708	0.616649	1.577591
C	-3.129929	-1.275779	1.265062
H	-3.729563	-1.805350	2.000393
C	-2.139119	-1.952702	0.554535
H	-1.973658	-3.011937	0.738185
C	3.683930	1.839132	-0.283087
H	2.690775	2.285925	-0.171055
H	4.456037	2.559816	-0.006293
H	3.776327	1.569886	-1.339960
C	1.838700	-0.712721	-0.478599
C	3.829263	0.619775	0.598676
O	4.713851	0.546815	1.456779
O	1.560169	0.149496	-1.317291
Cl	-2.875752	2.446774	-0.229725

TS2h′

C	-1.015377	-0.254008	0.661870
C	0.403966	0.409799	-0.621091
C	0.301067	1.835934	-0.353456
C	-0.994122	2.511216	-0.712936
C	-2.161197	1.958715	0.162382
H	-0.605562	0.217606	1.552606
H	-0.915988	3.594767	-0.576616
H	-1.240709	2.331611	-1.767566
H	-2.008460	2.277209	1.200573
H	-3.119326	2.348507	-0.191051
H	-0.105651	0.033865	-1.506659
C	1.667129	-0.211046	-0.316072
C	2.635992	0.431872	0.431561
H	3.551008	-0.087511	0.695191
C	2.441414	1.771384	0.795745
H	3.203247	2.283765	1.375235
C	1.288505	2.463846	0.382213
H	1.175356	3.514236	0.639023

C	-1.599707	-2.638012	-0.239058
H	-1.339003	-2.341290	-1.260921
H	-1.289202	-3.669077	-0.057753
H	-2.687444	-2.546287	-0.163945
C	-2.192128	0.438436	0.070322
C	-0.932788	-1.744674	0.783921
O	-0.302678	-2.207678	1.726671
O	-3.077315	-0.138681	-0.553069
Cl	1.962656	-1.825135	-0.917608

3h′

C	-0.860451	0.069226	0.476576
C	0.430693	0.186588	-0.458390
C	0.958600	1.605166	-0.350707
C	-0.011149	2.678760	-0.751433
C	-1.274978	2.613369	0.140730
H	-0.510093	0.218777	1.505825
H	0.450147	3.668860	-0.673655
H	-0.315807	2.547473	-1.800770
H	-0.998318	2.890970	1.167326
H	-2.057083	3.297660	-0.200158
H	0.077737	0.010404	-1.488312
C	1.501013	-0.823125	-0.134679
C	2.712348	-0.497560	0.413310
H	3.417365	-1.286646	0.655212
C	3.062944	0.853671	0.619711
H	4.022309	1.101612	1.061169
C	2.180350	1.878903	0.200394
H	2.488184	2.916893	0.308341
C	-2.212725	-1.798114	-0.804413
H	-1.631322	-1.543407	-1.695744
H	-2.336546	-2.881131	-0.740058
H	-3.196517	-1.326676	-0.902166
C	-1.851363	1.208318	0.168251
C	-1.581101	-1.293154	0.477478
O	-1.673537	-1.906170	1.522316
O	-3.033941	0.999920	-0.028206
Cl	1.172040	-2.497475	-0.537109

1j′

C	-3.965970	0.037470	0.830593
C	1.351981	1.592155	-0.460020
C	0.866858	0.273254	-0.686822
C	-0.576957	0.055484	-1.083777
C	-1.533382	0.157231	0.113989
H	-3.599434	0.227437	1.836982
H	-0.880025	0.789906	-1.839534
H	-0.697628	-0.927196	-1.551833
H	-1.266477	-0.583686	0.881879
H	-1.430877	1.132566	0.612424
H	0.679503	2.434720	-0.608059
C	2.651151	1.817206	-0.070410

C	3.554702	0.736926	0.118482
C	3.082134	-0.595744	-0.110439
C	1.732823	-0.787201	-0.512273
H	1.384753	-1.803671	-0.688506
C	-6.116798	-0.384285	-0.568305
H	-7.190558	-0.457518	-0.384132
H	-5.908613	0.411072	-1.290957
H	-5.745323	-1.308322	-1.022555
C	-2.998039	-0.039339	-0.261689
C	-5.422589	-0.112985	0.747581
O	-6.058692	-0.008469	1.800908
O	-3.344878	-0.252071	-1.426432
H	3.006346	2.832187	0.093006
C	3.981090	-1.681120	0.071158
H	3.622050	-2.692752	-0.103974
C	4.903487	0.930210	0.519324
H	5.258147	1.943977	0.691078
C	5.283033	-1.461384	0.461819
H	5.959624	-2.300878	0.597481
C	5.749445	-0.143018	0.688309
H	6.779258	0.017321	0.995784

TS1j′

C	3.829870	-0.317977	0.685546
C	-1.592844	-2.036183	-0.046514
C	-0.841706	-1.010068	-0.686452
C	0.550993	-1.296677	-1.206705
C	1.633726	-1.314215	-0.100477
H	3.746679	-1.006169	1.523789
H	0.555411	-2.260346	-1.729564
H	0.834418	-0.537932	-1.942069
H	1.195638	-1.085864	0.881145
H	2.081283	-2.311350	0.013377
H	-1.163850	-3.032928	0.033392
C	-2.847096	-1.791083	0.460923
C	-3.435800	-0.501575	0.362252
C	-2.692985	0.539946	-0.282171
C	-1.400134	0.247688	-0.794133
H	-0.843035	1.044115	-1.284074
C	5.319541	1.543167	-0.353493
H	6.260757	2.042258	-0.114246
H	5.381235	1.067378	-1.337348
H	4.508455	2.274914	-0.422973
C	2.775735	-0.321167	-0.327798
C	5.032395	0.520937	0.723340
O	5.801668	0.355550	1.675162
O	2.810551	0.436231	-1.299394
H	-3.409034	-2.588277	0.942492
C	-3.276412	1.831329	-0.388372
H	-2.712193	2.621331	-0.878729
C	-4.728539	-0.210723	0.873563
H	-5.289235	-1.004828	1.361512

C	-4.531757	2.082474	0.118620
H	-4.965707	3.074927	0.030823
C	-5.265505	1.051795	0.756008
H	-6.255458	1.262515	1.151511

2j′

C	-3.397700	0.166171	0.638078
C	1.354753	2.115242	0.624875
C	0.644457	1.554810	-0.473963
C	-0.581014	2.264052	-1.014224
C	-1.850311	2.010346	-0.180578
H	-3.623043	0.826732	1.472073
H	-0.397339	3.345330	-1.033046
H	-0.772449	1.950554	-2.044606
H	-1.661094	2.184522	0.886425
H	-2.634100	2.729486	-0.466053
H	1.017753	3.061244	1.044386
C	2.459891	1.491378	1.154087
C	2.930193	0.262861	0.617223
C	2.225843	-0.311538	-0.489956
C	1.087443	0.361635	-1.009162
H	0.549906	-0.084680	-1.841784
C	-3.930370	-2.164842	-0.385310
H	-4.567751	-3.018207	-0.145066
H	-4.174181	-1.776378	-1.379148
H	-2.881467	-2.474533	-0.431287
C	-2.443512	0.615867	-0.370467
C	-4.132506	-1.103469	0.672569
O	-4.925026	-1.267323	1.605031
O	-2.155056	-0.081916	-1.347289
H	2.994142	1.937300	1.990071
C	2.691350	-1.539185	-1.034174
H	2.155121	-1.974461	-1.874244
C	4.069113	-0.410550	1.133617
H	4.601498	0.029915	1.973639
C	3.798317	-2.168727	-0.510581
H	4.143190	-3.107162	-0.936467
C	4.494597	-1.599116	0.583869
H	5.367202	-2.105365	0.987751

TS2j′

C	1.359148	0.540989	0.677862
C	-0.257636	-2.657297	0.275111
C	0.600180	-1.807513	-0.430241
C	2.039839	-2.167334	-0.673742
C	2.966804	-1.387061	0.310517
H	0.968299	-0.000493	1.535661
H	2.197926	-3.243731	-0.548439
H	2.336018	-1.910099	-1.698956
H	2.774743	-1.735311	1.332391
H	4.016803	-1.565688	0.064220
H	0.098844	-3.643124	0.565029

C	-1.551113	-2.274122	0.605471
C	-2.069418	-1.009579	0.203069
C	-1.223180	-0.112707	-0.516504
C	0.171581	-0.469655	-0.732155
H	0.665141	0.031994	-1.563115
C	1.506757	2.956564	-0.291495
H	0.925093	3.879962	-0.241412
H	2.553287	3.153716	-0.039667
H	1.519532	2.567170	-1.315767
C	2.683654	0.104406	0.183773
C	0.913283	1.958857	0.683955
O	0.032866	2.294306	1.470288
O	3.469014	0.841934	-0.408771
H	-2.199502	-2.956872	1.148515
C	-1.762857	1.095851	-0.994533
H	-1.127114	1.775654	-1.555733
C	-3.415400	-0.641716	0.446009
H	-4.053474	-1.325306	1.000823
C	-3.087766	1.427867	-0.757555
H	-3.485805	2.366088	-1.132992
C	-3.916183	0.557217	-0.024353
H	-4.950994	0.827511	0.166169

3j′

C	-1.180662	-0.416267	0.457631
C	0.370538	2.696957	0.136946
C	-0.539231	1.830800	-0.426496
C	-1.952415	2.228204	-0.733478
C	-2.924318	1.496459	0.227627
H	-0.878354	-0.091288	1.461417
H	-2.085391	3.311500	-0.641973
H	-2.214118	1.957909	-1.767691
H	-2.778708	1.891359	1.242794
H	-3.972425	1.644346	-0.048625
H	0.077331	3.732952	0.298168
C	1.662349	2.293406	0.503691
C	2.125260	0.967703	0.218121
C	1.242777	0.021326	-0.373461
C	-0.229129	0.359040	-0.568108
H	-0.534135	0.026997	-1.573330
C	-1.464158	-2.728411	-0.774162
H	-0.914590	-3.672055	-0.813766
H	-2.533491	-2.940892	-0.673400
H	-1.333102	-2.173364	-1.708873
C	-2.647252	0.002826	0.260005
C	-1.029142	-1.945346	0.451894
O	-0.586849	-2.507221	1.434513
O	-3.544123	-0.813678	0.148756
H	2.349850	3.003939	0.953269
C	1.754135	-1.216420	-0.768008
H	1.104980	-1.933586	-1.262244
C	3.470571	0.601817	0.448765

H	4.136601	1.327768	0.909310
C	3.089921	-1.557870	-0.547126
H	3.456948	-2.531127	-0.859914
C	3.947538	-0.647069	0.078910
H	4.985688	-0.910198	0.262212

TS1j''

C	-3.954342	-0.432707	0.752558
C	0.889512	1.018355	-0.520401
C	0.802581	-0.397353	-0.651385
C	-0.521574	-1.032568	-1.016923
C	-1.561160	-1.003883	0.129548
H	-3.765044	-0.991658	1.666269
H	-0.955993	-0.505076	-1.873603
H	-0.355502	-2.068005	-1.331711
H	-1.784502	-2.015198	0.493946
H	-1.160410	-0.470788	1.004019
H	0.003039	1.618896	-0.713023
H	2.070279	1.629253	-0.171241
H	3.245191	0.866363	0.069920
C	3.172860	-0.557941	-0.062496
C	1.933931	-1.153157	-0.424661
H	1.890096	-2.236391	-0.524643
C	-5.758474	0.938285	-0.524271
H	-6.794411	1.243478	-0.363385
H	-5.121799	1.817380	-0.666053
H	-5.673123	0.352705	-1.445065
C	-2.880457	-0.320992	-0.232946
C	-5.305537	0.132480	0.672571
O	-6.058590	-0.078779	1.628229
O	-3.029263	0.306976	-1.283610
H	2.121266	2.712108	-0.081743
C	4.343657	-1.327159	0.174172
H	4.288364	-2.408749	0.073215
C	4.482234	1.462565	0.431848
H	4.532504	2.544661	0.529866
C	5.528720	-0.719686	0.525088
H	6.416503	-1.320615	0.703223
C	5.599473	0.688969	0.655256
H	6.540468	1.156725	0.931912

2j''

C	3.439292	-0.151776	-0.905281
C	-0.611149	0.011099	1.439191
C	-0.628531	-1.160912	0.631382
C	0.511421	-2.159539	0.688562
C	1.654072	-1.855846	-0.299504
H	3.369014	-0.547435	-1.915827
H	0.928334	-2.199188	1.700173
H	0.125316	-3.159029	0.458411
H	2.294020	-2.745657	-0.409694

H	1.256107	-1.649841	-1.301312
H	0.228893	0.172193	2.107701
C	-1.633381	0.929647	1.376363
C	-2.738704	0.740746	0.504716
C	-2.770289	-0.436265	-0.311203
C	-1.698865	-1.363505	-0.218180
H	-1.737652	-2.259978	-0.835620
C	4.663977	1.605211	0.571005
H	5.441875	2.359923	0.438658
H	3.740187	2.070202	0.929581
H	4.958982	0.886569	1.342141
C	2.573706	-0.714549	0.127297
C	4.438726	0.911973	-0.753847
O	5.090196	1.215466	-1.757771
O	2.610531	-0.310731	1.293554
H	-1.607508	1.817966	2.003628
C	-3.875285	-0.636462	-1.182380
H	-3.898942	-1.531205	-1.800471
C	-3.810874	1.668585	0.415506
H	-3.783479	2.560641	1.037228
C	-4.898425	0.282383	-1.247158
H	-5.736929	0.117463	-1.918506
C	-4.866319	1.446746	-0.440236
H	-5.680265	2.164273	-0.499753

TS2j''

C	1.739158	0.341262	-0.610770
C	0.447474	-0.151278	0.906868
C	0.103780	-1.517669	0.509503
C	1.218590	-2.528483	0.456547
C	2.254698	-2.145333	-0.643275
H	1.032504	0.165892	-1.418352
H	1.742785	-2.575228	1.420690
H	0.823082	-3.529237	0.253132
H	3.113296	-2.821325	-0.609682
H	1.774919	-2.225295	-1.626190
H	1.229671	-0.045257	1.658315
C	-0.594402	0.820115	0.938067
C	-1.881869	0.541891	0.439852
C	-2.183685	-0.790418	-0.003097
C	-1.153497	-1.788232	0.056423
H	-1.398558	-2.794505	-0.279221
C	3.167904	2.261313	0.432232
H	3.113971	3.348163	0.527270
H	3.130636	1.791843	1.422317
H	4.127039	1.961071	0.000272
C	2.748212	-0.724503	-0.398679
C	2.023186	1.796219	-0.444810
O	1.278099	2.603008	-0.990915
O	3.866797	-0.519563	0.065401
H	-0.380430	1.815560	1.315507

C	-3.479562	-1.076903	-0.475739
H	-3.706480	-2.085700	-0.812609
C	-2.905964	1.528792	0.393768
H	-2.674180	2.537015	0.727139
C	-4.455544	-0.093730	-0.513365
H	-5.449956	-0.330986	-0.880949
C	-4.165181	1.216969	-0.074203
H	-4.937250	1.980357	-0.109452

3j''

C	1.672121	0.284441	-0.430214
C	0.512113	-0.047593	0.607708
C	0.041012	-1.470230	0.395837
C	1.114446	-2.522855	0.466811
C	2.256698	-2.220205	-0.535941
H	1.221799	0.235085	-1.429532
H	1.544421	-2.551392	1.479924
H	0.698863	-3.516831	0.270094
H	3.101084	-2.903291	-0.408527
H	1.871098	-2.331632	-1.558626
H	0.990319	0.008107	1.604123
C	-0.592061	0.961712	0.537771
C	-1.920558	0.633213	0.246817
C	-2.284944	-0.742280	0.028966
C	-1.248939	-1.752144	0.119414
H	-1.545080	-2.788006	-0.042321
C	2.918391	2.100767	1.001442
H	3.115044	3.174741	0.992801
H	2.322588	1.841454	1.884902
H	3.861341	1.548381	1.070209
C	2.759407	-0.796312	-0.362989
C	2.219648	1.711839	-0.285425
O	2.073706	2.501735	-1.198768
O	3.934914	-0.543156	-0.173771
H	-0.334011	2.005507	0.699554
C	-3.613728	-1.065450	-0.260810
H	-3.879552	-2.107613	-0.424238
C	-2.943767	1.622533	0.158041
H	-2.676581	2.664121	0.318508
C	-4.594202	-0.076382	-0.342424
H	-5.621456	-0.346660	-0.569034
C	-4.249712	1.271960	-0.130374
H	-5.014386	2.041316	-0.195299

Syn
1a

C	2.729449	0.584814	0.043985
C	-2.920640	0.215354	-1.179773
C	-2.241648	-0.390356	-0.113622
C	-0.840233	-0.933905	-0.293775
C	0.234226	0.130473	-0.025035

H	2.430044	1.626452	0.154243
H	-0.670492	-1.783357	0.376804
H	-0.706582	-1.320131	-1.310324
H	0.095371	0.993868	-0.692727
H	0.127457	0.535500	0.992296
H	-2.444777	0.262035	-2.157439
C	-4.198585	0.748752	-1.007328
C	-4.822063	0.683929	0.240079
H	-5.818927	1.094873	0.375628
C	-4.159060	0.080362	1.310106
H	-4.639196	0.017786	2.283352
C	-2.881133	-0.451270	1.131998
H	-2.374146	-0.927412	1.968998
C	5.096789	1.486226	0.196151
H	6.128808	1.137715	0.266410
H	4.980652	2.102964	-0.703774
H	4.870606	2.126177	1.058497
C	1.659992	-0.394092	-0.196018
C	4.162214	0.283926	0.156751
O	4.590280	-0.863461	0.231358
O	1.886595	-1.554177	-0.524375
H	-4.709661	1.209355	-1.848941

TS1a

C	2.670453	-0.240378	0.563453
C	-2.342640	1.023562	-0.339850
C	-2.184024	-0.370346	-0.351425
C	-0.896281	-0.988101	-0.855959
C	0.261844	-0.928486	0.168845
H	2.492170	-0.599895	1.576215
H	-1.076199	-2.029960	-1.141377
H	-0.565736	-0.463829	-1.758935
H	-0.053005	-0.399886	1.080081
H	0.551919	-1.932702	0.506991
H	-1.542798	1.652478	-0.725516
C	-3.512043	1.607244	0.147070
C	-4.548249	0.804851	0.630501
H	-5.461609	1.258205	1.006418
C	-4.404929	-0.583049	0.620588
H	-5.207691	-1.216838	0.988946
C	-3.231559	-1.162951	0.133688
H	-3.129427	-2.246260	0.125049
C	5.017047	0.253559	1.393708
H	5.989633	0.584189	1.024653
H	4.659126	0.954945	2.157673
H	5.127020	-0.724798	1.878597
C	1.517756	-0.215442	-0.348364
C	4.039546	0.171128	0.228534
O	4.389270	0.420450	-0.920790
O	1.549827	0.360253	-1.429941
H	-3.617701	2.689174	0.142309

2a

C	-2.332144	0.207078	0.616264
C	1.994999	-0.438368	-1.050177
C	2.000415	0.643423	-0.158354
C	1.067354	1.823963	-0.345155
C	-0.265632	1.678754	0.413777
H	-2.231978	0.364827	1.689043
H	1.561114	2.736588	0.008886
H	0.845547	1.966161	-1.407286
H	-0.088873	1.384401	1.456374
H	-0.774624	2.654470	0.458887
H	1.313491	-0.423695	-1.895492
C	2.845480	-1.527858	-0.854221
C	3.715953	-1.555121	0.236724
H	4.380353	-2.402176	0.386232
C	3.731593	-0.483422	1.131377
H	4.410784	-0.490675	1.980224
C	2.881234	0.604611	0.931988
H	2.907332	1.441432	1.627886
C	-4.397649	-1.135007	1.228486
H	-5.255266	-1.623449	0.762208
H	-3.818861	-1.875708	1.794261
H	-4.753332	-0.385231	1.946591
C	-1.248683	0.704535	-0.239685
C	-3.541395	-0.485203	0.149694
O	-3.860216	-0.525807	-1.034097
O	-1.144020	0.373863	-1.417599
H	2.829039	-2.355693	-1.558563

TS2a

C	-0.959604	0.000485	0.485278
C	0.614134	-0.238918	-0.806855
C	1.520258	0.766428	-0.300141
C	1.070392	2.201819	-0.335049
C	-0.152369	2.413482	0.608843
H	-0.449468	-0.202876	1.426298
H	1.883011	2.871496	-0.033361
H	0.772480	2.487469	-1.352289
H	0.170112	2.257292	1.646029
H	-0.539837	3.430935	0.509941
H	-0.051777	0.028561	-1.626410
C	1.033862	-1.612850	-0.758427
C	2.191654	-1.975692	-0.096929
H	2.481662	-3.021601	-0.040737
C	3.018439	-0.990037	0.470503
H	3.934107	-1.274670	0.980787
C	2.675362	0.366854	0.356278
H	3.331771	1.122215	0.783102
C	-2.353531	-2.064624	1.084672
H	-3.055706	-2.773701	0.641109
H	-1.470642	-2.602829	1.454187
H	-2.819407	-1.578696	1.950960

C	-1.266799	1.438903	0.244814
C	-1.943127	-1.033302	0.045849
O	-2.360398	-1.072389	-1.100641
O	-2.300358	1.820279	-0.286663
H	0.407725	-2.362002	-1.234195

3a

C	0.785161	-0.115861	-0.308699
C	-0.540362	-0.346422	0.520203
C	-1.521403	0.773334	0.261734
C	-1.001842	2.160558	0.506523
C	0.263683	2.425732	-0.346896
H	0.541959	-0.250872	-1.373087
H	-1.765494	2.912772	0.280529
H	-0.733716	2.284860	1.567224
H	-0.023756	2.444726	-1.407826
H	0.732679	3.383212	-0.102386
H	-0.205821	-0.306735	1.572695
C	-1.117455	-1.712512	0.261782
C	-2.385284	-1.893442	-0.213606
H	-2.751833	-2.902455	-0.388822
C	-3.242038	-0.792891	-0.469306
H	-4.246991	-0.954388	-0.845934
C	-2.780624	0.521774	-0.216657
H	-3.449573	1.360548	-0.402823
C	2.986255	-1.356241	-0.928881
H	3.691827	-2.092955	-0.539366
H	2.557268	-1.715468	-1.872924
H	3.509142	-0.416171	-1.136359
C	1.298280	1.330364	-0.159475
C	1.895997	-1.103365	0.089121
O	1.885461	-1.643722	1.178115
O	2.462546	1.577530	0.095612
H	-0.481956	-2.563711	0.484205

NMR spectra of starting materials and products

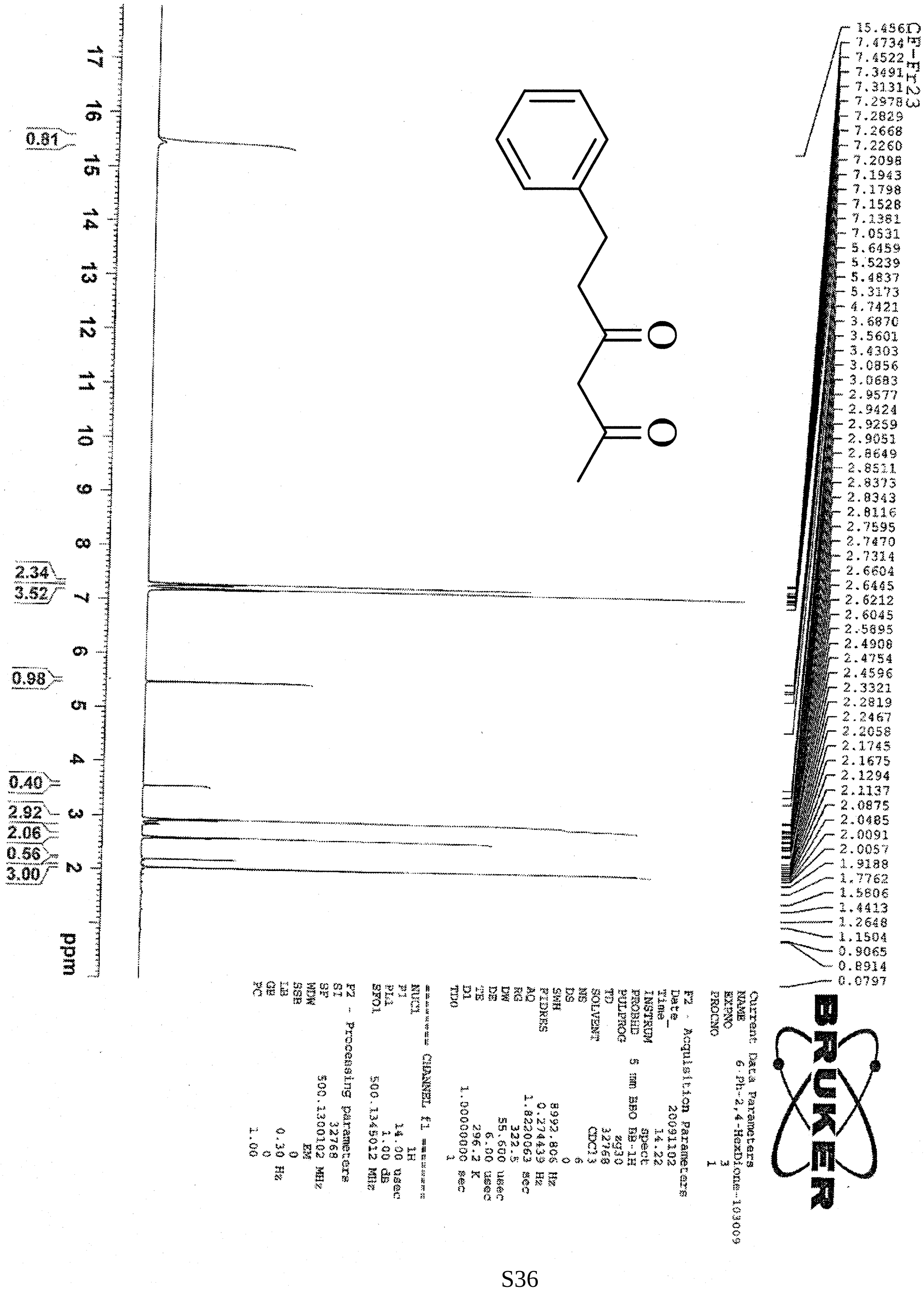

1a
13C

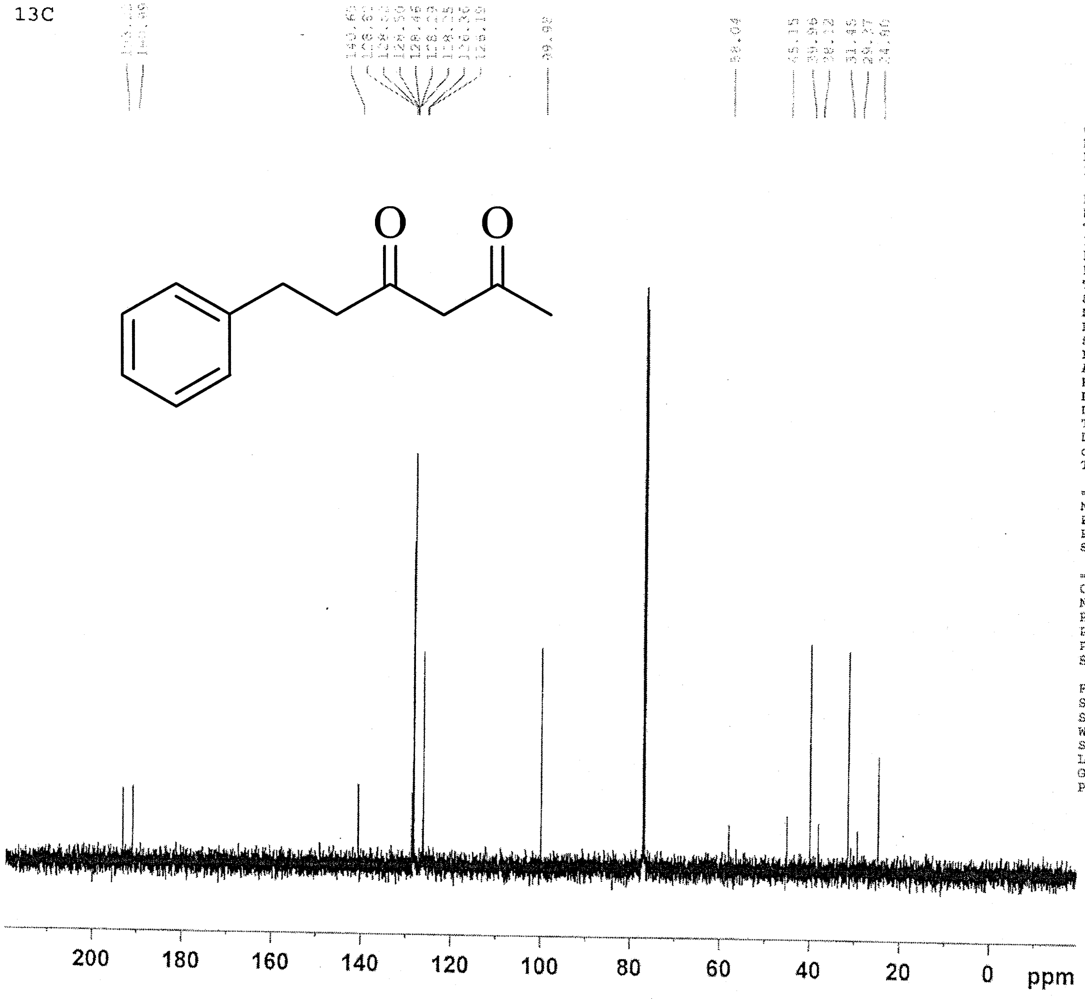

Current Data Parameters
NAME 6-Ph-2,4-PentDione-090209
EXPNO 5
PROCNO 1

F2 - Acquisition Parameters
Date_ 20090921
Time 15.14
INSTRUM spect
PROBHD 5 mm BBO BB-1H
PULPROG zgdc30
TD 65536
SOLVENT CDCl3
NS 29
DS 4
SWH 30030.029 Hz
FIDRES 0.458222 Hz
AQ 1.0912410 sec
RG 2580.3
DW 16.650 usec
DE 7.00 usec
TE 297.2 K
D1 1.00000000 sec
d11 0.03000000 sec
TD0 1

======== CHANNEL f1 ========
NUC1 13C
P1 8.60 usec
PL1 1.00 dB
SFO1 125.7703640 MHz

======== CHANNEL f2 ========
CPDPRG2 waltz16
NUC2 1H
PCPD2 100.00 usec
PL2 1.00 dB
PL12 17.50 dB
SFO2 500.1322150 MHz

F2 - Processing parameters
SI 32768
SF 125.7577962 MHz
WDW EM
SSB 0
LB 1.00 Hz
GB 0
PC 1.40

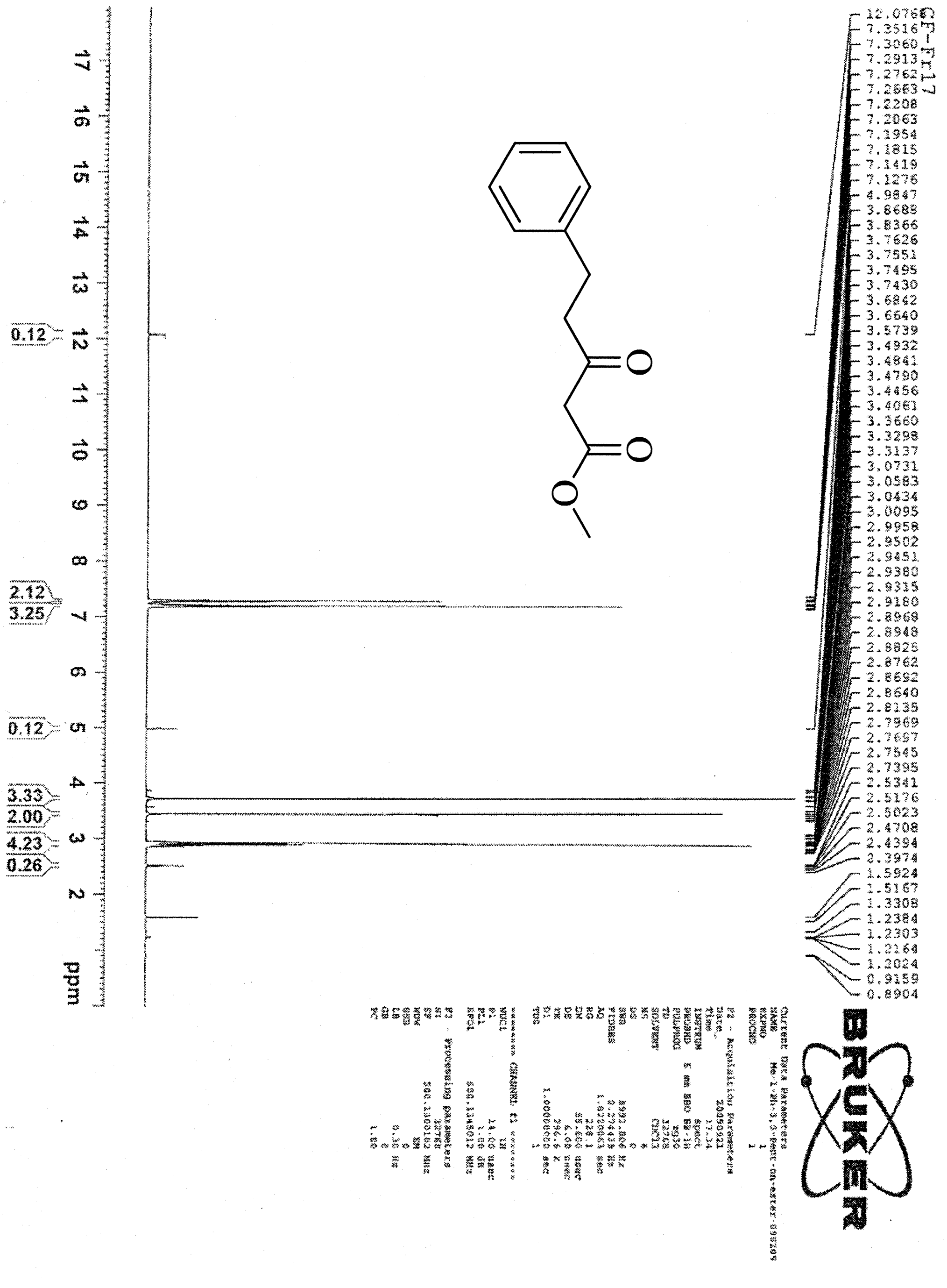
GF-Fr17
ppm
BRUKER

CF-Fr17
13C

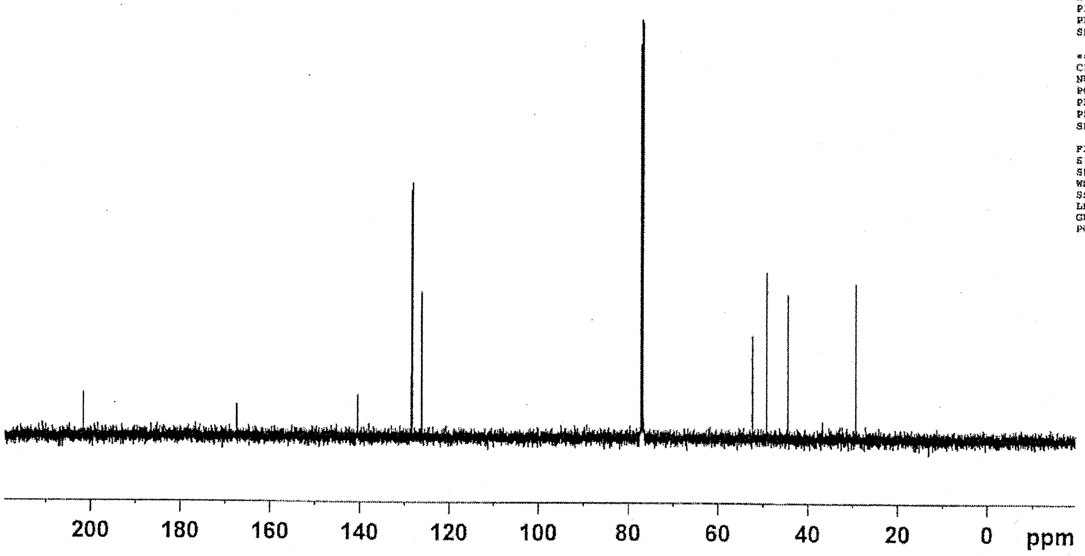

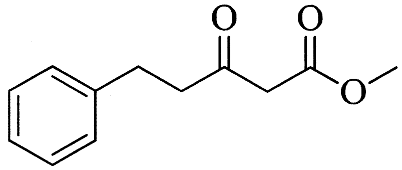

BRUKER

```
Current Data Parameters
NAME      Me-1-Ph-3,5-Pent-on-ester-090209
EXPNO                   2
PROCNO                  1

F2 - Acquisition Parameters
Date_            20090921
Time                17.41
INSTRUM             spect
PROBHD   5 mm BBO BB-1H
PULPROG           zgdc30
TD                 65536
SOLVENT            CDCl3
NS                    65
DS                     4
SWH            30030.029 Hz
FIDRES          0.458222 Hz
AQ             1.0912410 sec
RG                  4096
DW                16.650 usec
DE                  7.00 usec
TE                 296.9 K
D1            1.00000000 sec
d11           0.03000000 sec
TD0                    1

======== CHANNEL f1 ========
NUC1                 13C
P1                  8.60 usec
PL1                 1.00 dB
SFO1         125.7703640 MHz

======== CHANNEL f2 ========
CPDPRG2           waltz16
NUC2                  1H
PCPD2             100.00 usec
PL2                 1.00 dB
PL12               17.50 dB
SFO2         500.1322150 MHz

F2 - Processing parameters
SI                 32768
SF           125.7577942 MHz
WDW                   EM
SSB                    0
LB                  1.00 Hz
GB                     0
PC                  1.40
```

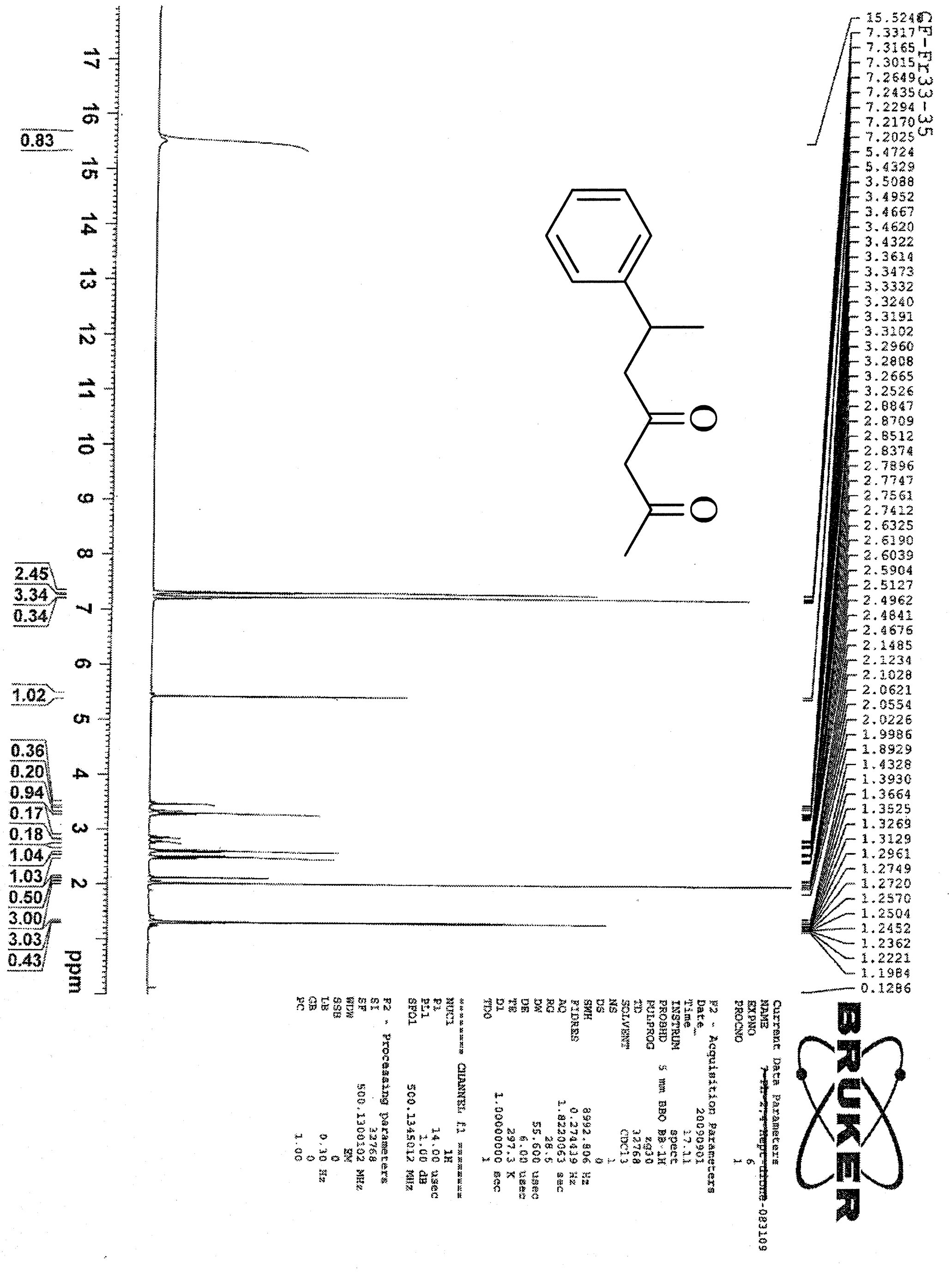

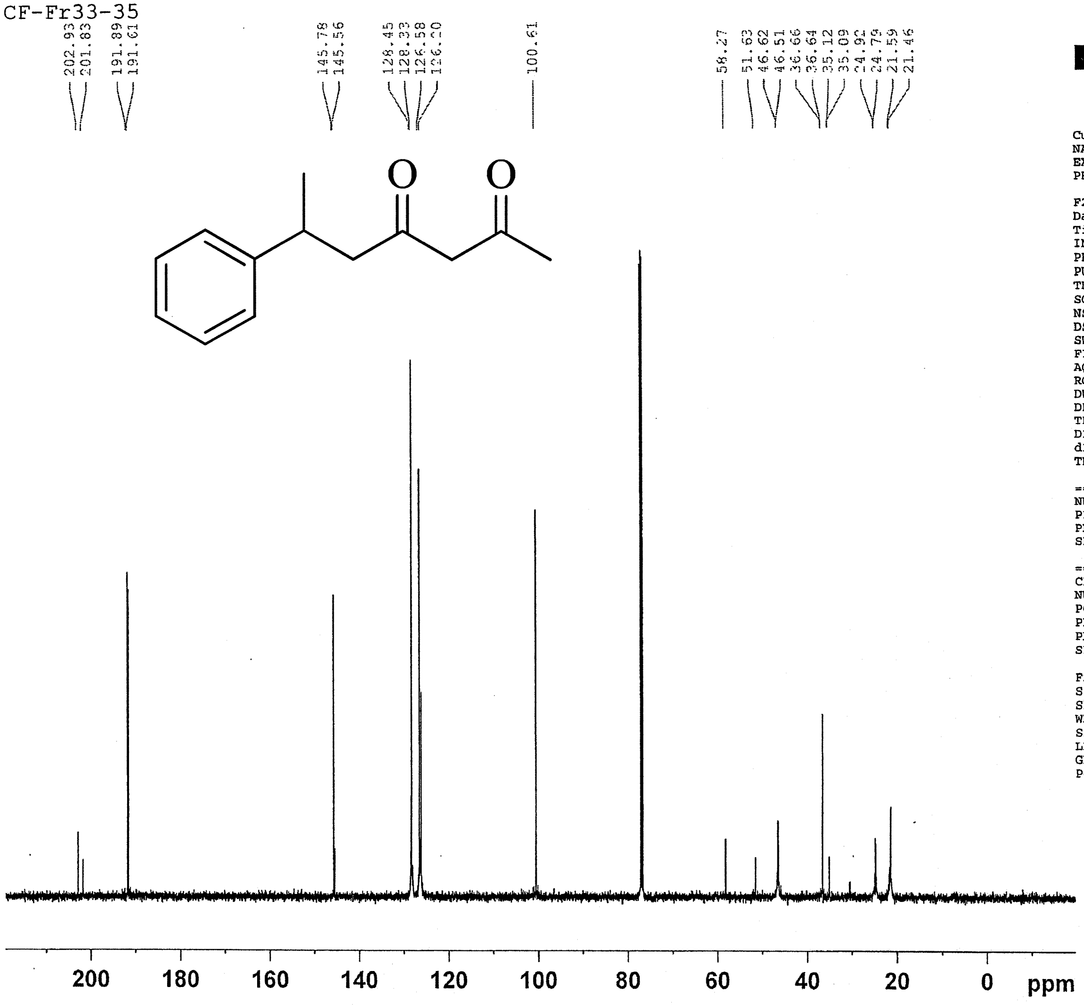


```
Current Data Parameters
NAME      7-Ph-2,4-Hept-dione-083109
EXPNO                  7
PROCNO                 1

F2 - Acquisition Parameters
Date_           20090901
Time               17.03
INSTRUM            spect
PROBHD   5 mm BBO BB-1H
PULPROG          zgdc30
TD                65536
SOLVENT           CDCl3
NS                  214
DS                    4
SWH           30030.029 Hz
FIDRES         0.458222 Hz
AQ            1.0912410 sec
RG               5160.6
DW               16.650 usec
DE                 7.00 usec
TE                297.2 K
D1           1.00000000 sec
d11          0.03000000 sec
TD0                   1

======== CHANNEL f1 ========
NUC1                 13C
P1                  5.00 usec
PL1                 1.00 dB
SFO1         125.7703640 MHz

======== CHANNEL f2 ========
CPDPRG2          waltz16
NUC2                  1H
PCPD2              90.00 usec
PL2                24.00 dB
PL12               24.00 dB
SFO2         500.1322150 MHz

F2 - Processing parameters
SI                 32768
SF           125.7578145 MHz
WDW                   EM
SSB                    0
LB                  1.00 Hz
GB                     0
PC                  1.40
```

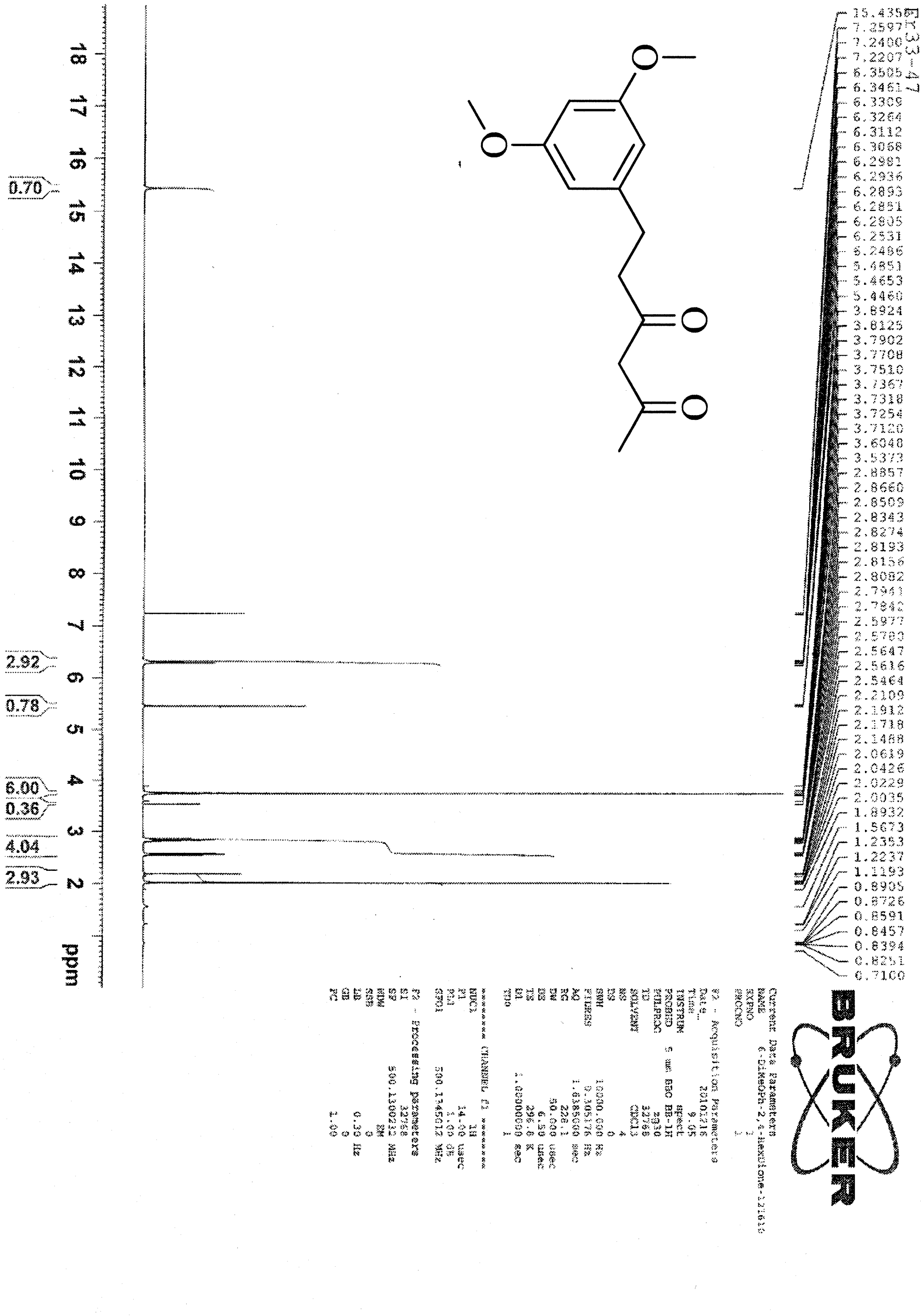

Fr33-47

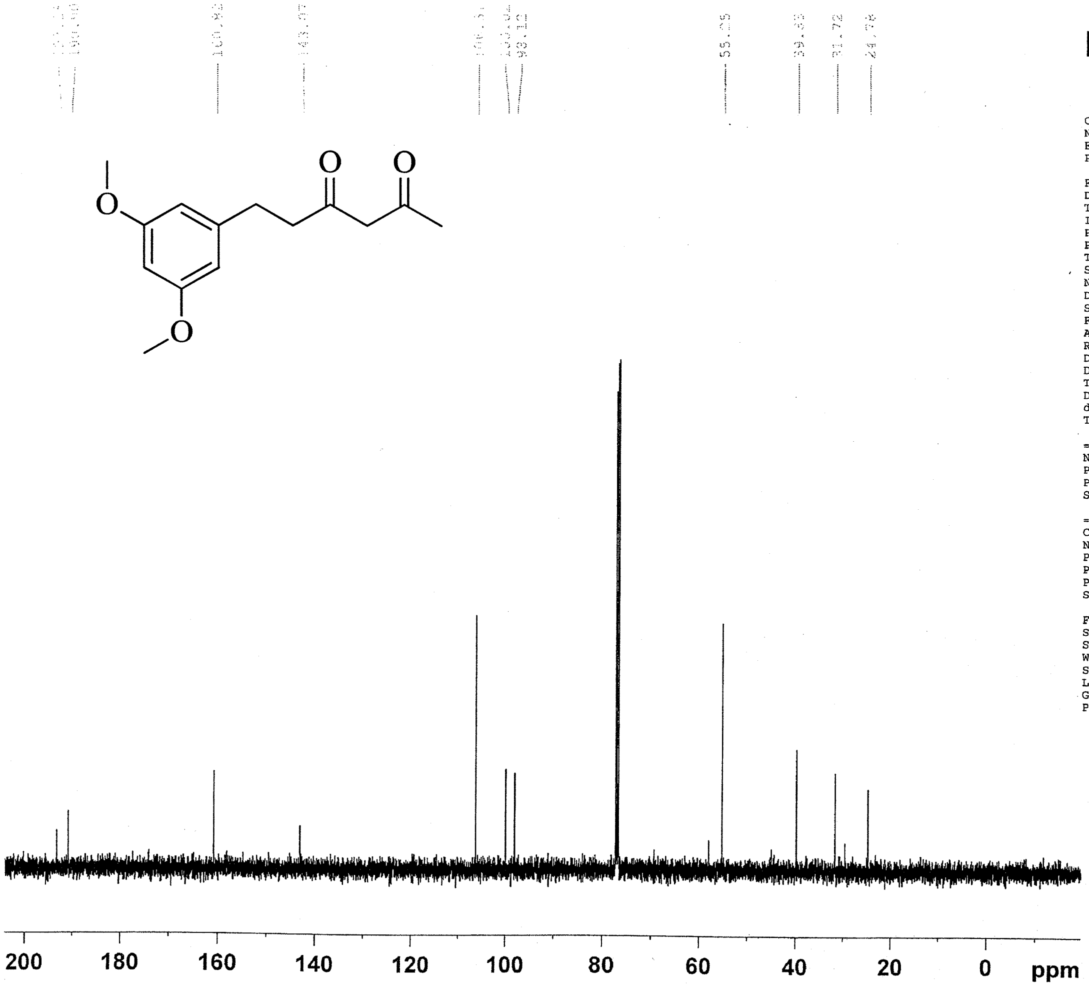

BRUKER

```
Current Data Parameters
NAME      6-DiMeOPh-2,4-HexDione-121610
EXPNO                    2
PROCNO                   1

F2 - Acquisition Parameters
Date_             20101216
Time                  9.12
INSTRUM              spect
PROBHD   5 mm BBO BB-1H
PULPROG            zgdc30
TD                   65536
SOLVENT              CDCl3
NS                      52
DS                       4
SWH             30030.029 Hz
FIDRES           0.458222 Hz
AQ              1.0912410 sec
RG                  7298.2
DW                  16.650 usec
DE                    7.00 usec
TE                   297.2 K
D1             1.00000000 sec
d11            0.03000000 sec
TD0                      1

======== CHANNEL f1 ========
NUC1                   13C
P1                    8.60 usec
PL1                   1.00 dB
SFO1           125.7703640 MHz

======== CHANNEL f2 ========
CPDPRG2            waltz16
NUC2                    1H
PCPD2               100.00 usec
PL2                   1.00 dB
PL12                 17.50 dB
SFO2           500.1322150 MHz

F2 - Processing parameters
SI                   32768
SF             125.7577932 MHz
WDW                     EM
SSB                      0
LB                    1.00 Hz
GB                       0
PC                    1.40
```

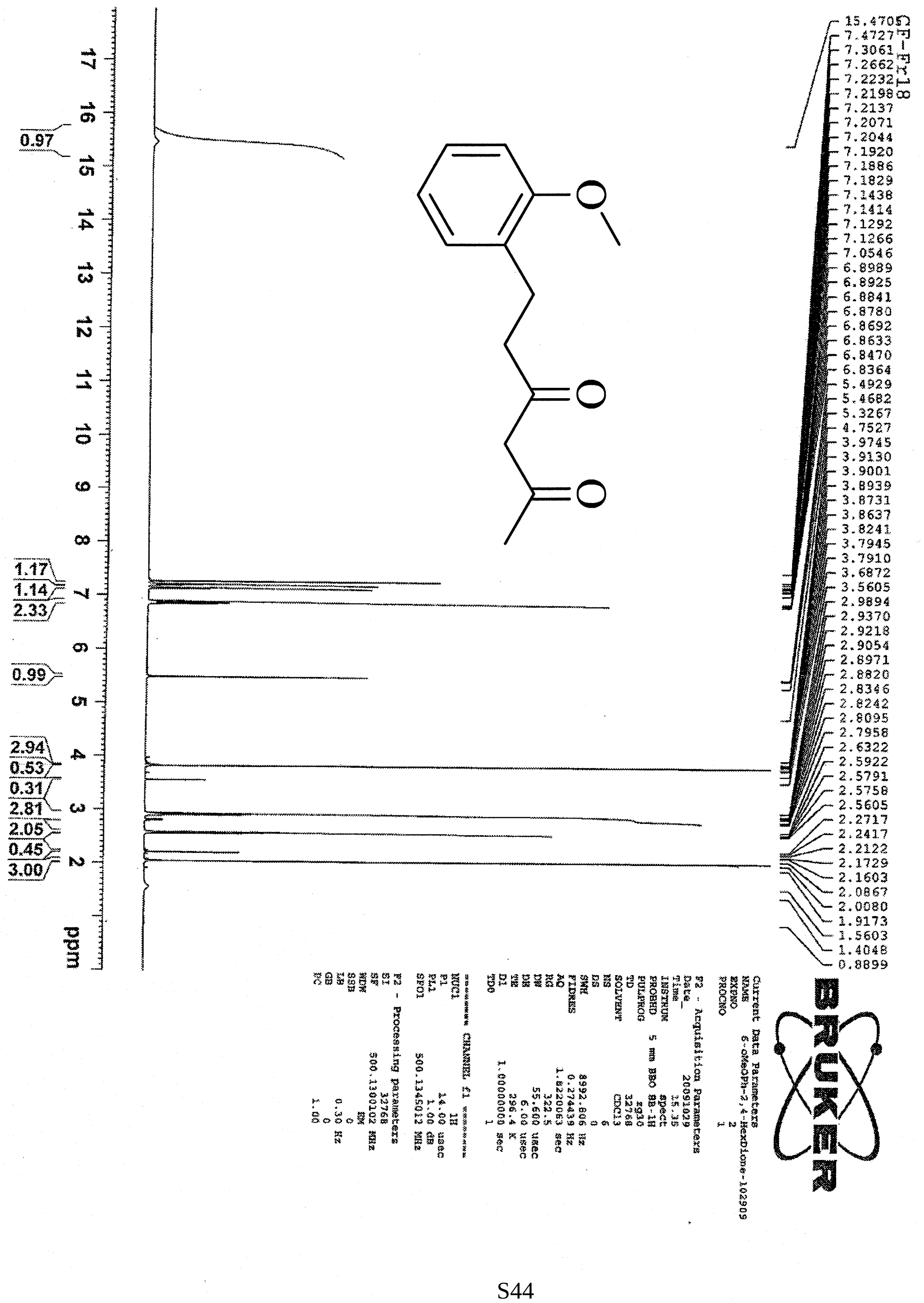
GF-Fr18
15.4705
7.4727
7.3061
7.2662
7.2232
7.2198
7.2137
7.2071
7.2044
7.1920
7.1886
7.1829
7.1438
7.1414
7.1292
7.1266
7.0546
6.8989
6.8925
6.8841
6.8780
6.8692
6.8633
6.8470
6.8364
5.4929
5.4682
5.3267
4.7527
3.9745
3.9130
3.9001
3.8939
3.8731
3.8637
3.8241
3.7945
3.7910
3.6872
3.5605
2.9894
2.9370
2.9218
2.9054
2.8971
2.8820
2.8346
2.8242
2.8095
2.7958
2.6322
2.5922
2.5791
2.5758
2.5605
2.2717
2.2417
2.2122
2.1729
2.1603
2.0867
2.0080
1.9173
1.5603
1.4048
0.8899
0.97
1.17
1.14
2.33
0.99
2.94
0.53
0.31
2.81
2.05
0.45
3.00
ppm

CF-Fr18
13C NMR

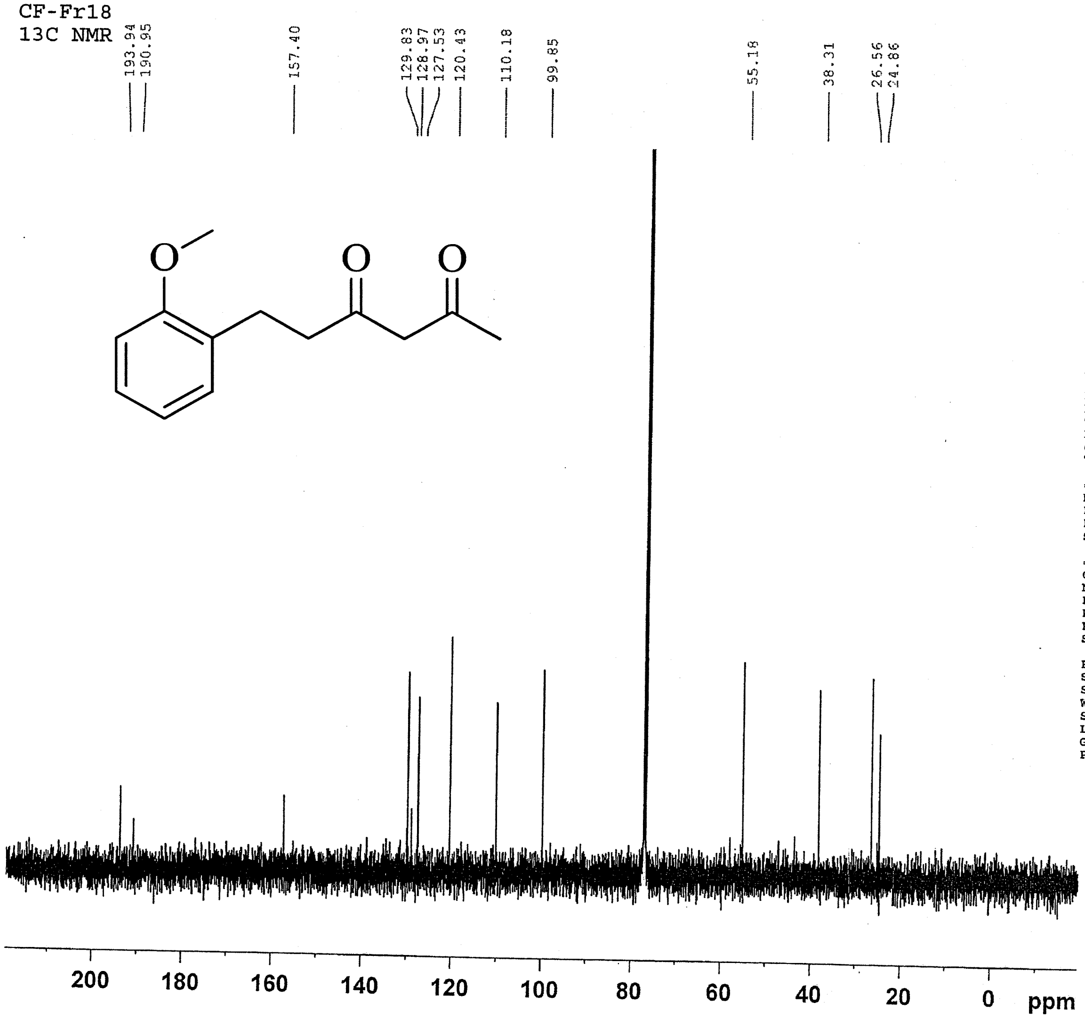

BRUKER

```
Current Data Parameters
NAME          6-oMeOPh-2,4-HexDione-102909
EXPNO                    3
PROCNO                   1

F2 - Acquisition Parameters
Date_             20091029
Time                 15.38
INSTRUM              spect
PROBHD    5 mm BBO BB-1H
PULPROG             zgdc30
TD                   65536
SOLVENT              CDCl3
NS                     291
DS                       4
SWH              30030.029 Hz
FIDRES            0.458222 Hz
AQ               1.0912410 sec
RG                    4096
DW                  16.650 usec
DE                    7.00 usec
TE                   296.8 K
D1              1.00000000 sec
d11             0.03000000 sec
TD0                      1

======== CHANNEL f1 ========
NUC1                   13C
P1                    8.60 usec
PL1                   1.00 dB
SFO1           125.7703640 MHz

======== CHANNEL f2 ========
CPDPRG2            waltz16
NUC2                    1H
PCPD2               100.00 usec
PL2                   1.00 dB
PL12                 17.50 dB
SFO2           500.1322150 MHz

F2 - Processing parameters
SI                   32768
SF             125.7577924 MHz
WDW                     EM
SSB                      0
LB                    1.00 Hz
GB                       0
PC                    1.40
```

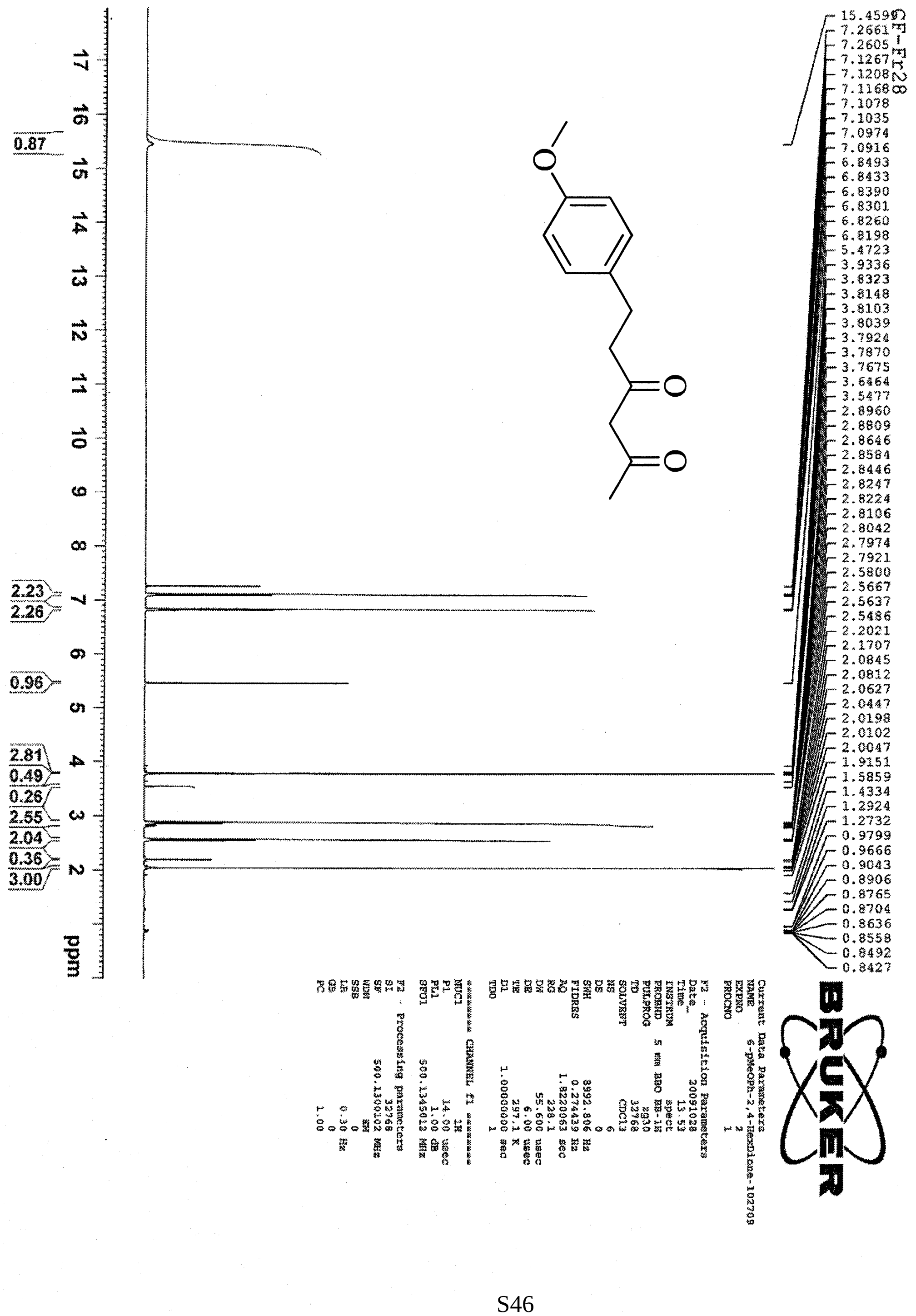

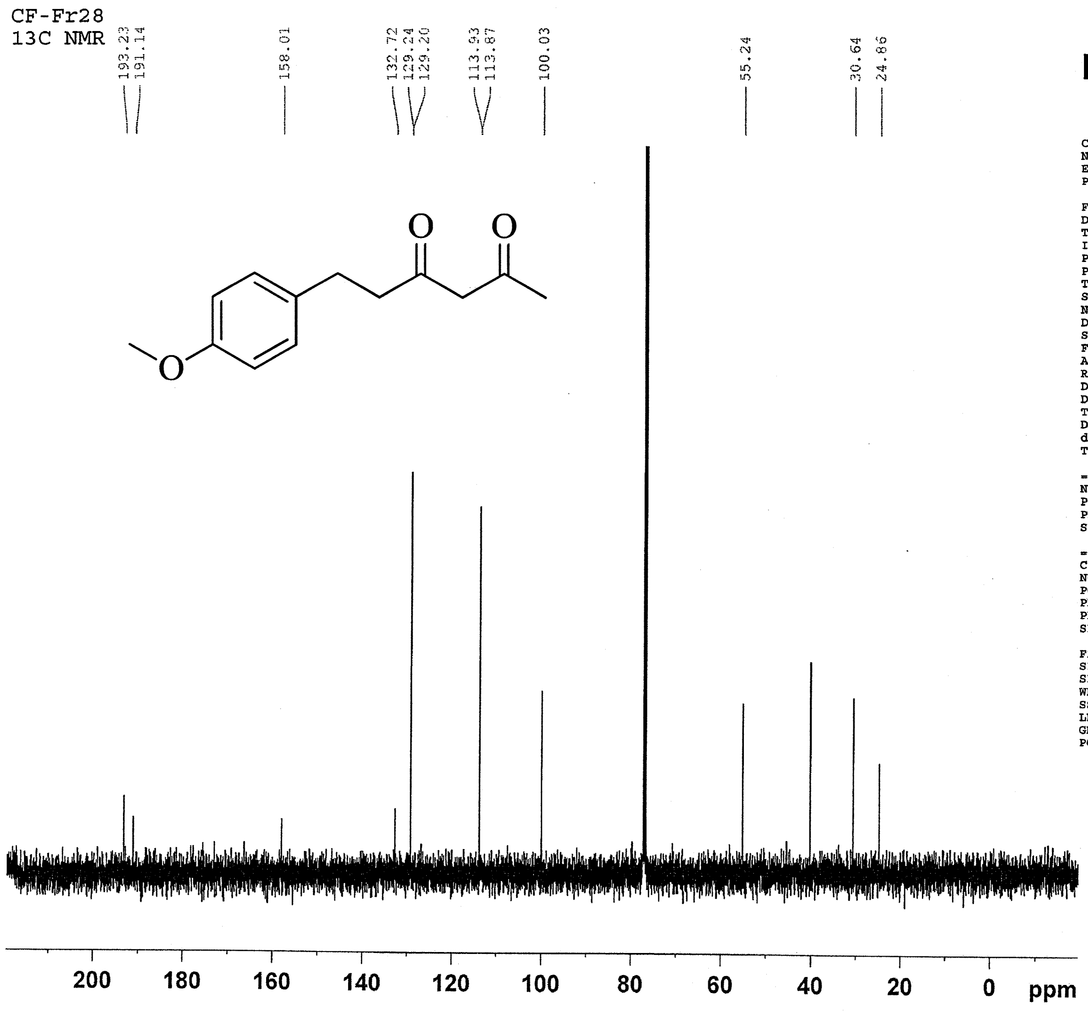

BRUKER

```
Current Data Parameters
NAME      6-pMeOPh-2,4-HexDione-102709
EXPNO                    3
PROCNO                   1

F2 - Acquisition Parameters
Date_             20091028
Time                 13.56
INSTRUM              spect
PROBHD   5 mm BBO BB-1H
PULPROG             zgdc30
TD                   65536
SOLVENT              CDCl3
NS                      48
DS                       4
SWH          30030.029 Hz
FIDRES        0.458222 Hz
AQ           1.0912410 sec
RG                  1448.2
DW             16.650 usec
DE               7.00 usec
TE               297.4 K
D1          1.00000000 sec
d11         0.03000000 sec
TD0                      1

======== CHANNEL f1 ========
NUC1                   13C
P1               8.60 usec
PL1               1.00 dB
SFO1      125.7703640 MHz

======== CHANNEL f2 ========
CPDPRG2            waltz16
NUC2                    1H
PCPD2          100.00 usec
PL2               1.00 dB
PL12             17.50 dB
SFO2      500.1322150 MHz

F2 - Processing parameters
SI                   32768
SF        125.7577929 MHz
WDW                     EM
SSB                      0
LB                1.00 Hz
GB                       0
PC                    1.40
```

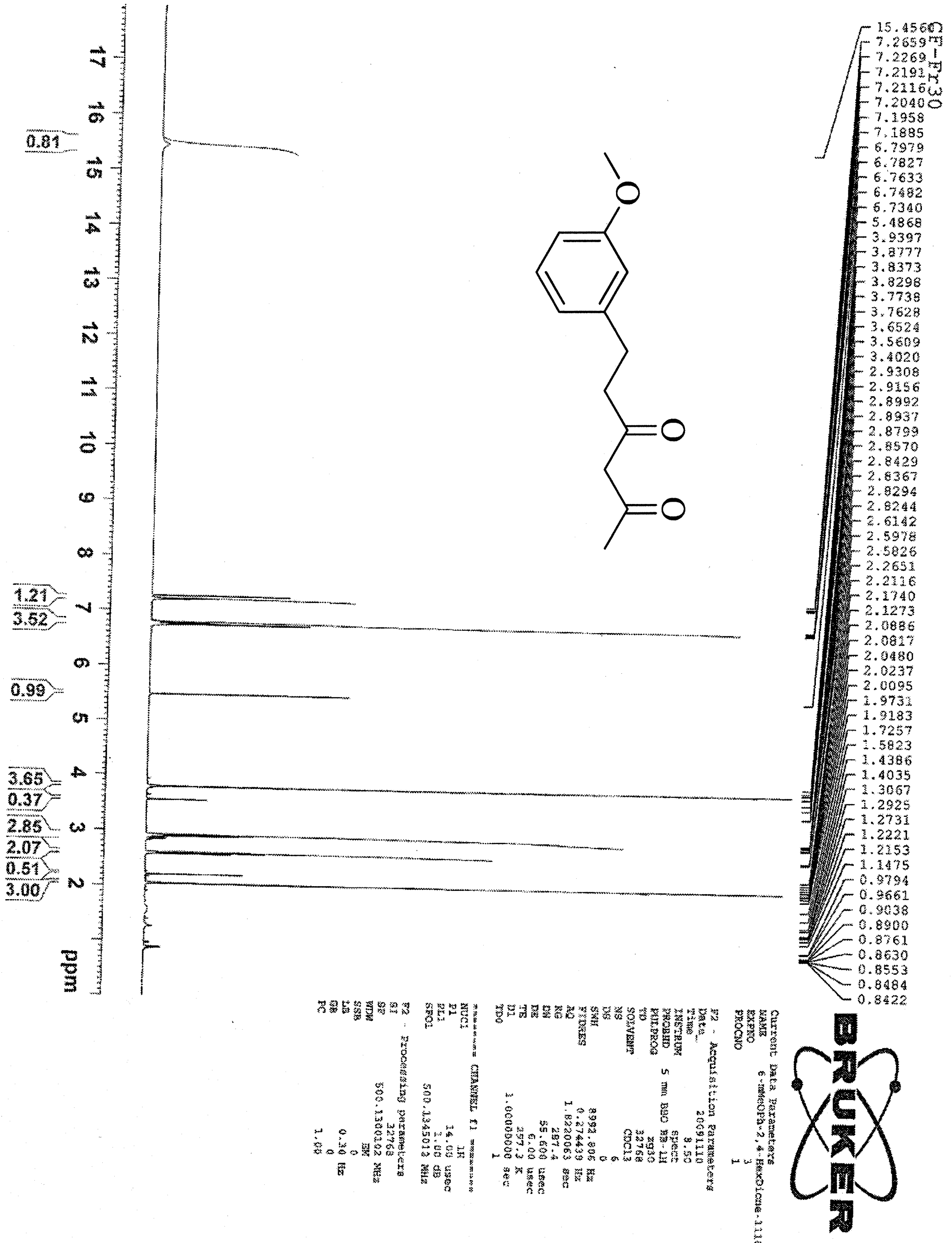
CF-Fr30
15.4560
7.2659
7.2269
7.2191
7.2116
7.2040
7.1958
7.1885
6.7979
6.7827
6.7633
6.7482
6.7340
5.4868
3.9397
3.8777
3.8373
3.8298
3.7738
3.7628
3.6524
3.5609
3.4020
2.9308
2.9156
2.8992
2.8937
2.8799
2.8570
2.8429
2.8367
2.8294
2.8244
2.6142
2.5978
2.5826
2.2651
2.2116
2.1740
2.1273
2.0886
2.0817
2.0480
2.0237
2.0095
1.9731
1.9183
1.7257
1.5823
1.4386
1.4035
1.3067
1.2925
1.2731
1.2221
1.2153
1.1475
0.9794
0.9661
0.9038
0.8900
0.8761
0.8630
0.8553
0.8484
0.8422
0.81
1.21
3.52
0.99
3.65
0.37
2.85
2.07
0.51
3.00
ppm
Current Data Parameters

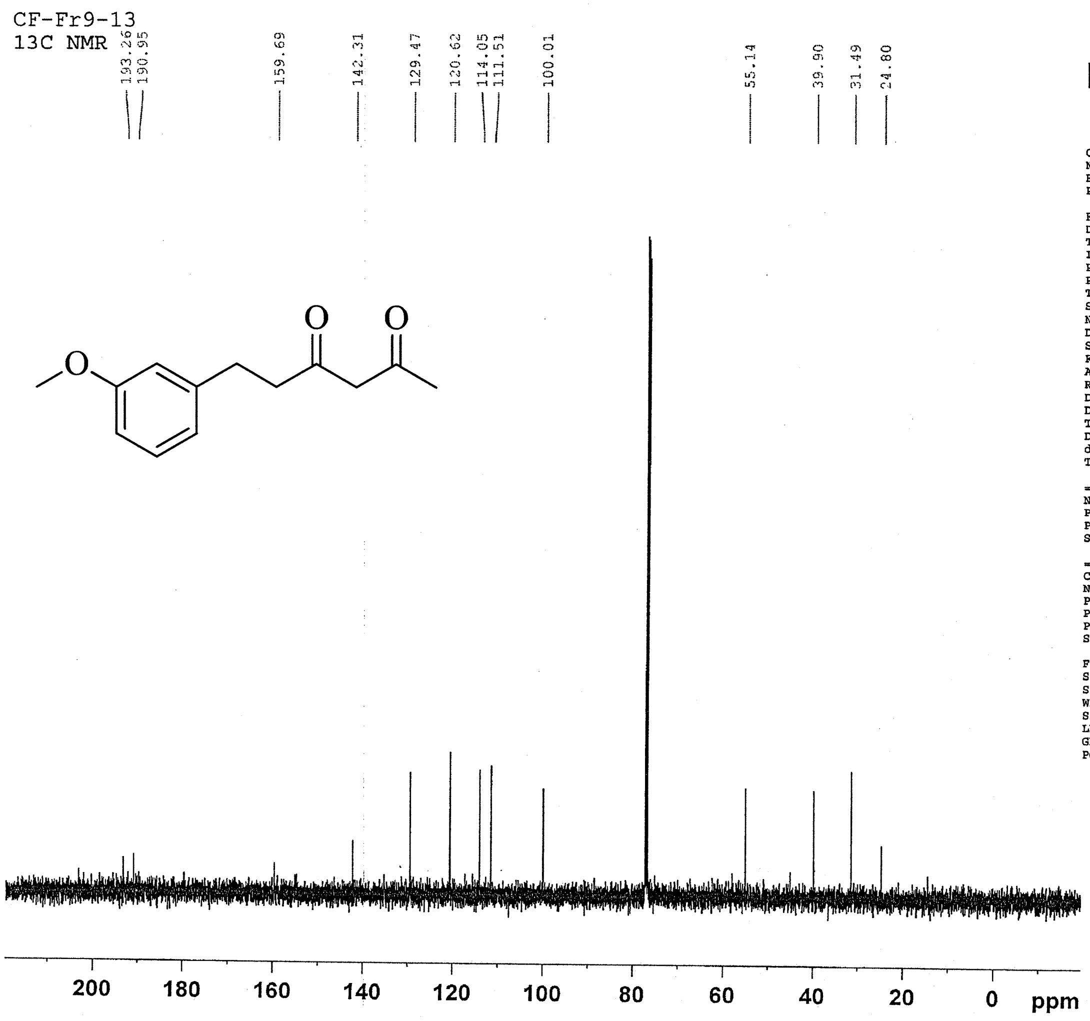


```
Current Data Parameters
NAME       6-mMeOPh-2,4-HexDione-102109
EXPNO                    4
PROCNO                   1

F2 - Acquisition Parameters
Date_             20091021
Time                 14.49
INSTRUM              spect
PROBHD   5 mm BBO BB-1H
PULPROG            zgdc30
TD                   65536
SOLVENT              CDCl3
NS                     105
DS                       4
SWH              30030.029 Hz
FIDRES            0.458222 Hz
AQ               1.0912410 sec
RG                  2896.3
DW                  16.650 usec
DE                    7.00 usec
TE                   297.6 K
D1              1.00000000 sec
d11             0.03000000 sec
TD0                      1

======== CHANNEL f1 ========
NUC1                   13C
P1                    8.60 usec
PL1                   1.00 dB
SFO1           125.7703640 MHz

======== CHANNEL f2 ========
CPDPRG2            waltz16
NUC2                    1H
PCPD2               100.00 usec
PL2                   1.00 dB
PL12                 17.50 dB
SFO2           500.1322150 MHz

F2 - Processing parameters
SI                   32768
SF             125.7577920 MHz
WDW                     EM
SSB                      0
LB                    1.00 Hz
GB                       0
PC                    1.40
```

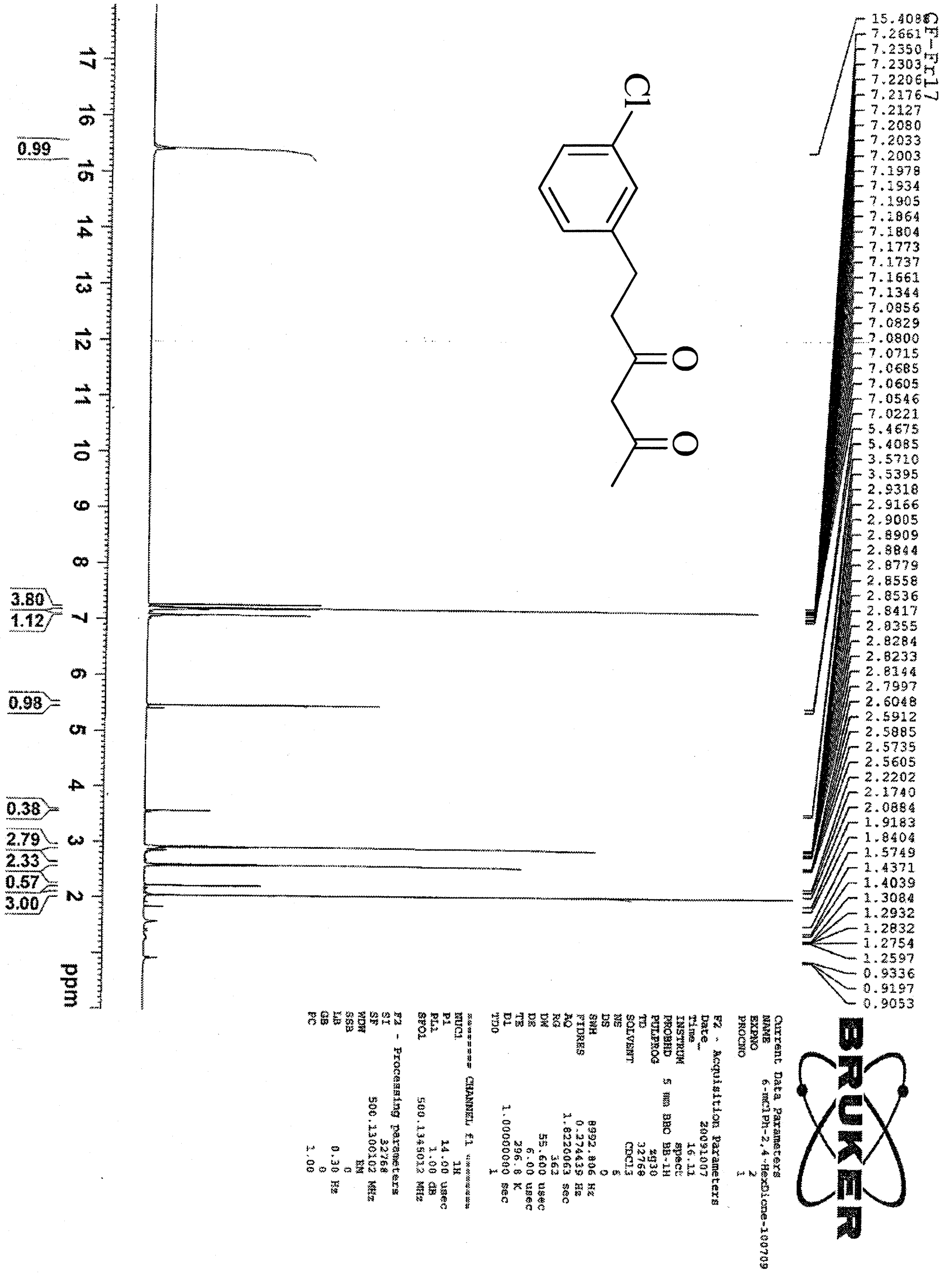
CF-Fr17
Cl
O
O
15.4088
7.2661
7.2350
7.2303
7.2206
7.2176
7.2127
7.2080
7.2033
7.2003
7.1978
7.1934
7.1905
7.1864
7.1804
7.1773
7.1737
7.1661
7.1344
7.0856
7.0829
7.0800
7.0715
7.0685
7.0605
7.0546
7.0221
5.4675
5.4085
3.5710
3.5395
2.9318
2.9166
2.9005
2.8909
2.8844
2.8779
2.8558
2.8536
2.8417
2.8355
2.8284
2.8233
2.8144
2.7997
2.6048
2.5912
2.5885
2.5735
2.5605
2.2202
2.1740
2.0884
1.9183
1.8404
1.5749
1.4371
1.4039
1.3084
1.2932
1.2832
1.2754
1.2597
0.9336
0.9197
0.9053
0.99
3.80
1.12
0.98
0.38
2.79
2.33
0.57
3.00
ppm
BRUKER
Current Data Parameters
NAME 6-mClPh-2,4-HexDione-100709
EXPNO 2
PROCNO 1

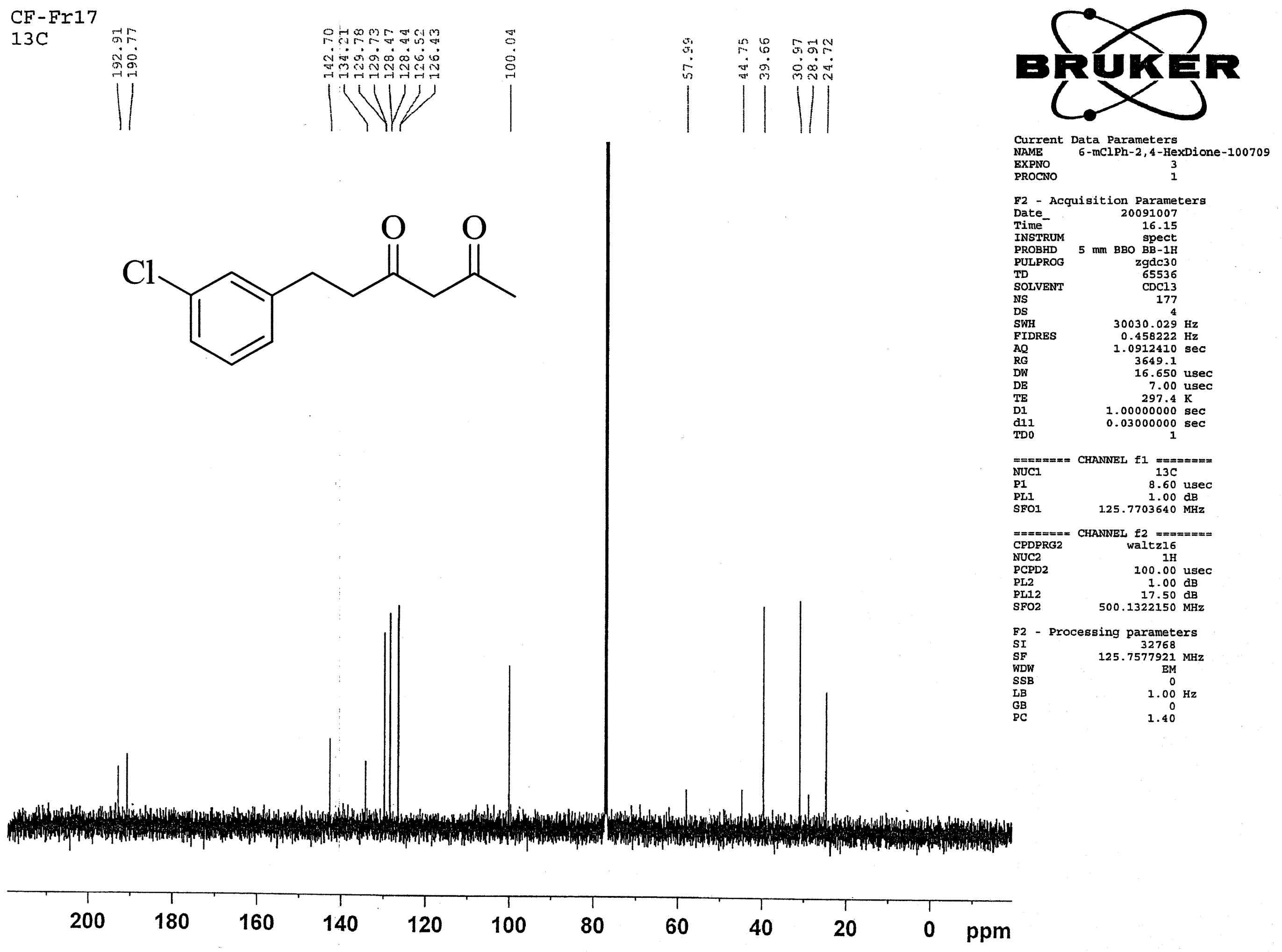

CF-Fr17
13C
192.91
190.77
142.70
134.21
129.78
129.73
128.47
128.44
126.52
126.43
100.04
57.99
44.75
39.66
30.97
28.91
24.72
Cl
O
O
200 180 160 140 120 100 80 60 40 20 0 ppm
BRUKER
Current Data Parameters
NAME 6-mClPh-2,4-HexDione-100709
EXPNO 3
PROCNO 1
F2 - Acquisition Parameters
Date_ 20091007
Time 16.15
INSTRUM spect
PROBHD 5 mm BBO BB-1H
PULPROG zgdc30
TD 65536
SOLVENT CDCl3
NS 177
DS 4
SWH 30030.029 Hz
FIDRES 0.458222 Hz
AQ 1.0912410 sec
RG 3649.1
DW 16.650 usec
DE 7.00 usec
TE 297.4 K
D1 1.00000000 sec
d11 0.03000000 sec
TD0 1
======== CHANNEL f1 ========
NUC1 13C
P1 8.60 usec
PL1 1.00 dB
SFO1 125.7703640 MHz
======== CHANNEL f2 ========
CPDPRG2 waltz16
NUC2 1H
PCPD2 100.00 usec
PL2 1.00 dB
PL12 17.50 dB
SFO2 500.1322150 MHz
F2 - Processing parameters
SI 32768
SF 125.7577921 MHz
WDW EM
SSB 0
LB 1.00 Hz
GB 0
PC 1.40

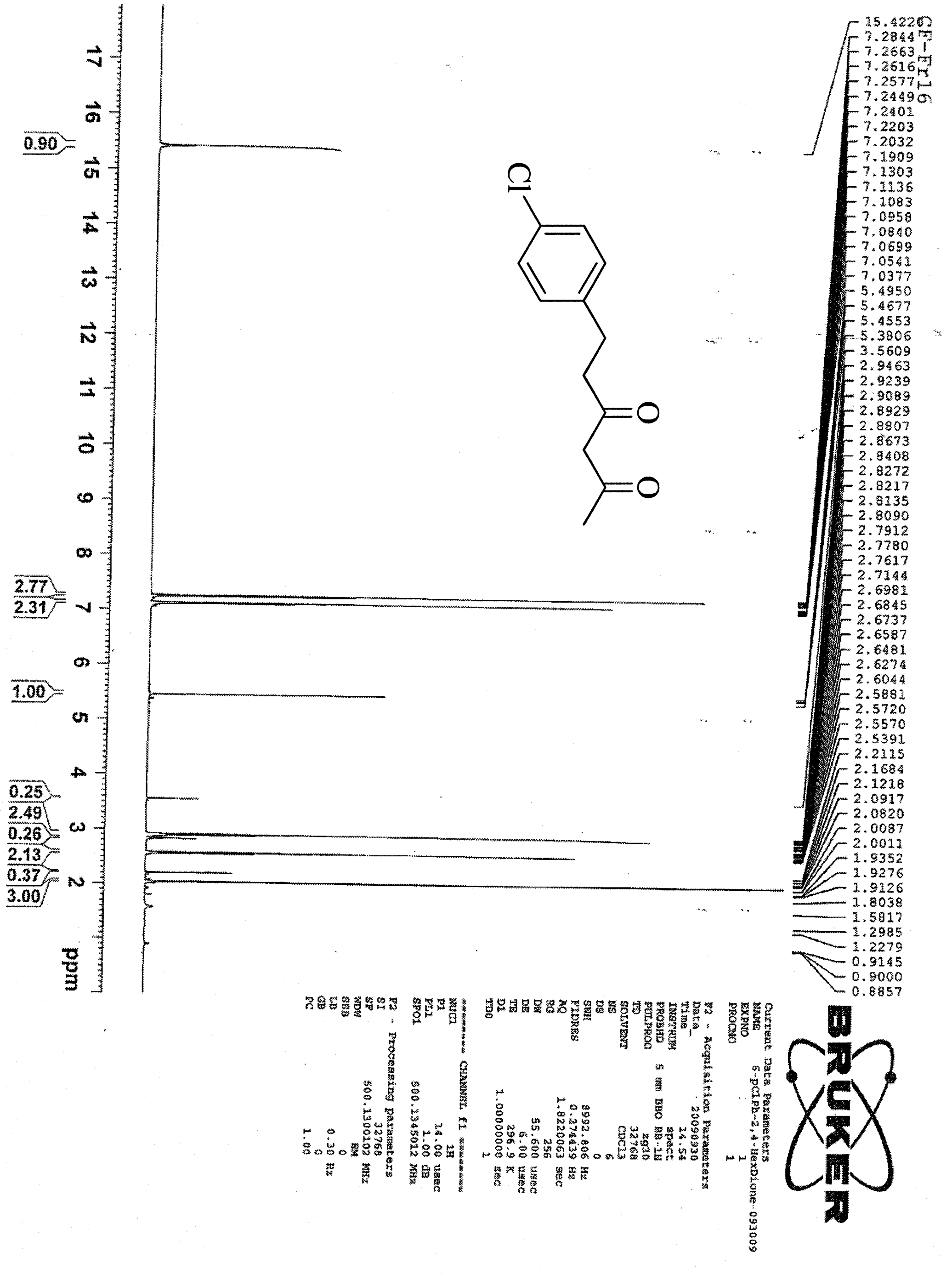

CF-Fr16
Cl
O
O
15.4220
7.2844
7.2663
7.2616
7.2577
7.2449
7.2401
7.2203
7.2032
7.1909
7.1303
7.1136
7.1083
7.0958
7.0840
7.0699
7.0541
7.0377
5.4950
5.4677
5.4553
5.3806
3.5609
2.9463
2.9239
2.9089
2.8929
2.8807
2.8673
2.8408
2.8272
2.8217
2.8135
2.8090
2.7912
2.7780
2.7617
2.7144
2.6981
2.6845
2.6737
2.6587
2.6481
2.6274
2.6044
2.5881
2.5720
2.5570
2.5391
2.2115
2.1684
2.1218
2.0917
2.0820
2.0087
2.0011
1.9352
1.9276
1.9126
1.8038
1.5817
1.2985
1.2279
0.9145
0.9000
0.8857
0.90
2.77
2.31
1.00
0.25
2.49
0.26
2.13
0.37
3.00
ppm
BRUKER
Current Data Parameters
NAME 6-pClPh-2,4-HexDione-093009
EXPNO 1
PROCNO 1
F2 - Acquisition Parameters
Date_ 20090930
Time 14.54
INSTRUM spect
PROBHD 5 mm BBO BB-1H
PULPROG zg30
TD 32768
SOLVENT CDCl3
NS 6
DS 0
SWH 8992.806 Hz
FIDRES 0.274439 Hz
AQ 1.8220063 sec
RG 256
DW 55.600 usec
DE 6.00 usec
TE 296.9 K
D1 1.00000000 sec
TD0 1
======== CHANNEL f1 ========
NUC1 1H
P1 14.00 usec
PL1 1.00 dB
SFO1 500.1345012 MHz
F2 - Processing parameters
SI 32768
SF 500.1300102 MHz
WDW EM
SSB 0
LB 0.30 Hz
GB 0
PC 1.00

CF-Fr16
13C NMR

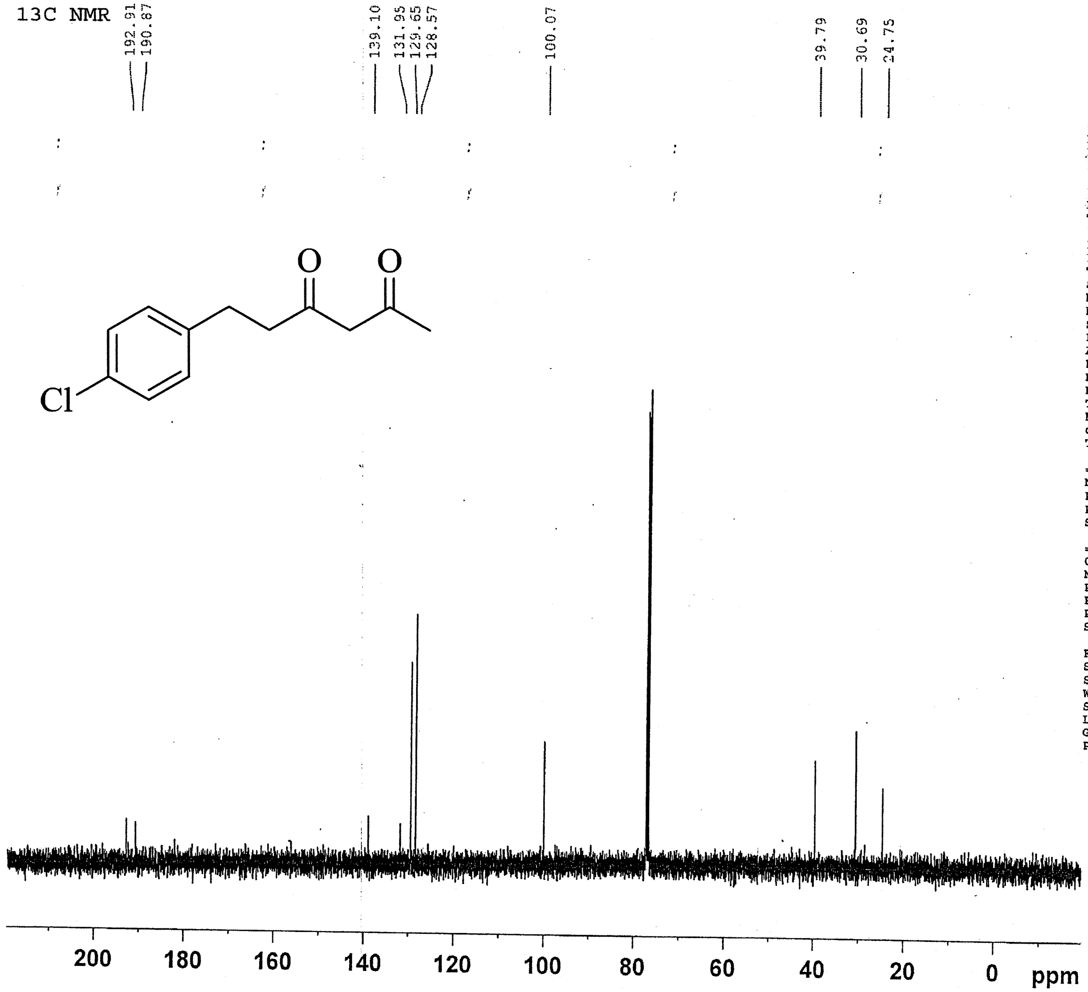

BRUKER

Current Data Parameters		
NAME	6-pClPh-2,4-HexDione-093009	
EXPNO	4	
PROCNO	1	
F2 - Acquisition Parameters		
Date_	20090930	
Time	15.19	
INSTRUM	spect	
PROBHD	5 mm BBO BB-1H	
PULPROG	zgdc30	
TD	65536	
SOLVENT	CDCl3	
NS	35	
DS	4	
SWH	30030.029	Hz
FIDRES	0.458222	Hz
AQ	1.0912410	sec
RG	4096	
DW	16.650	usec
DE	7.00	usec
TE	297.4	K
D1	1.00000000	sec
d11	0.03000000	sec
TD0	1	
======== CHANNEL f1 ========		
NUC1	13C	
P1	8.60	usec
PL1	1.00	dB
SFO1	125.7703640	MHz
======== CHANNEL f2 ========		
CPDPRG2	waltz16	
NUC2	1H	
PCPD2	100.00	usec
PL2	1.00	dB
PL12	17.50	dB
SFO2	500.1322150	MHz
F2 - Processing parameters		
SI	32768	
SF	125.7577935	MHz
WDW	EM	
SSB	0	
LB	1.00	Hz
GB	0	
PC	1.40	

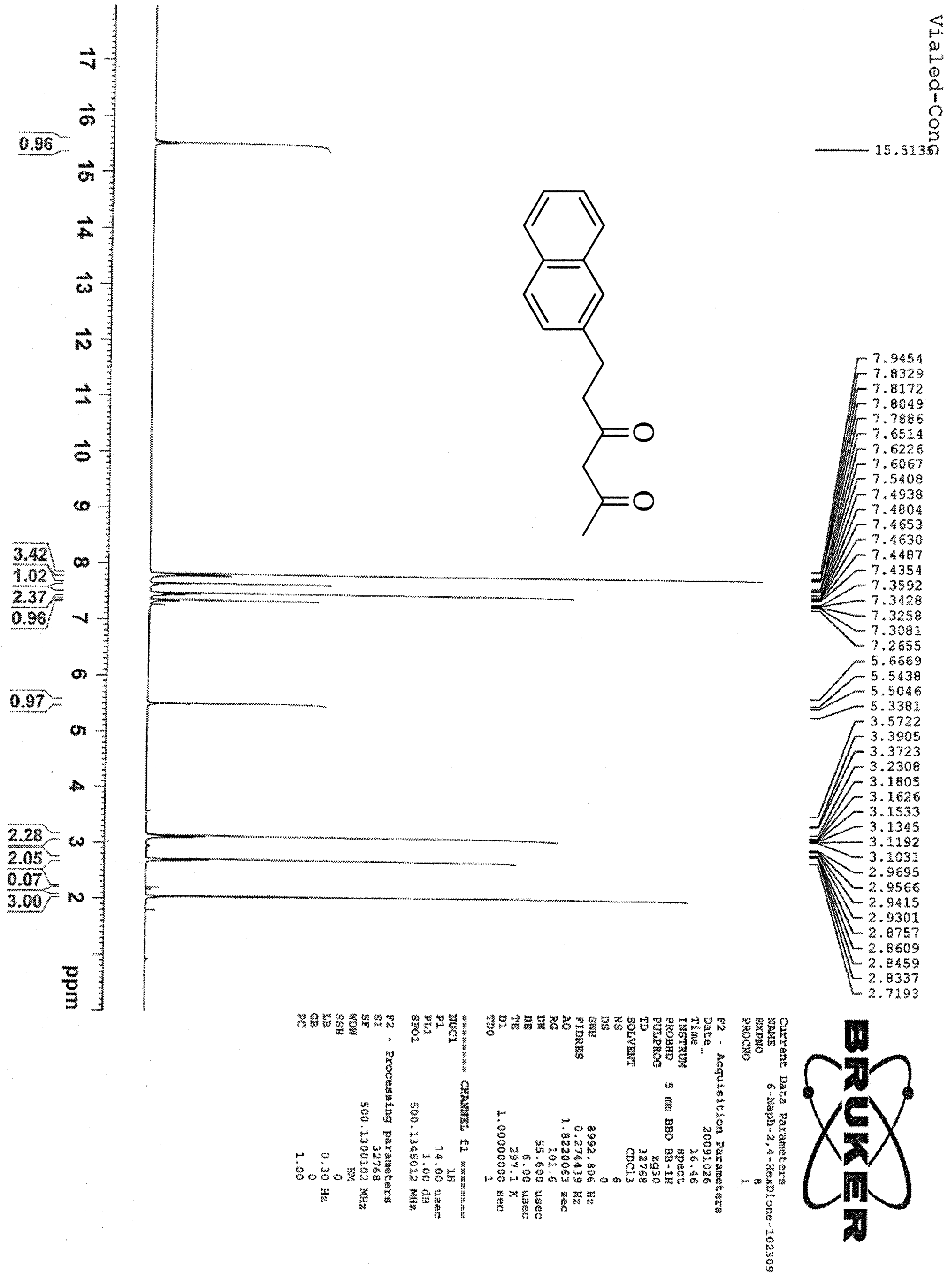

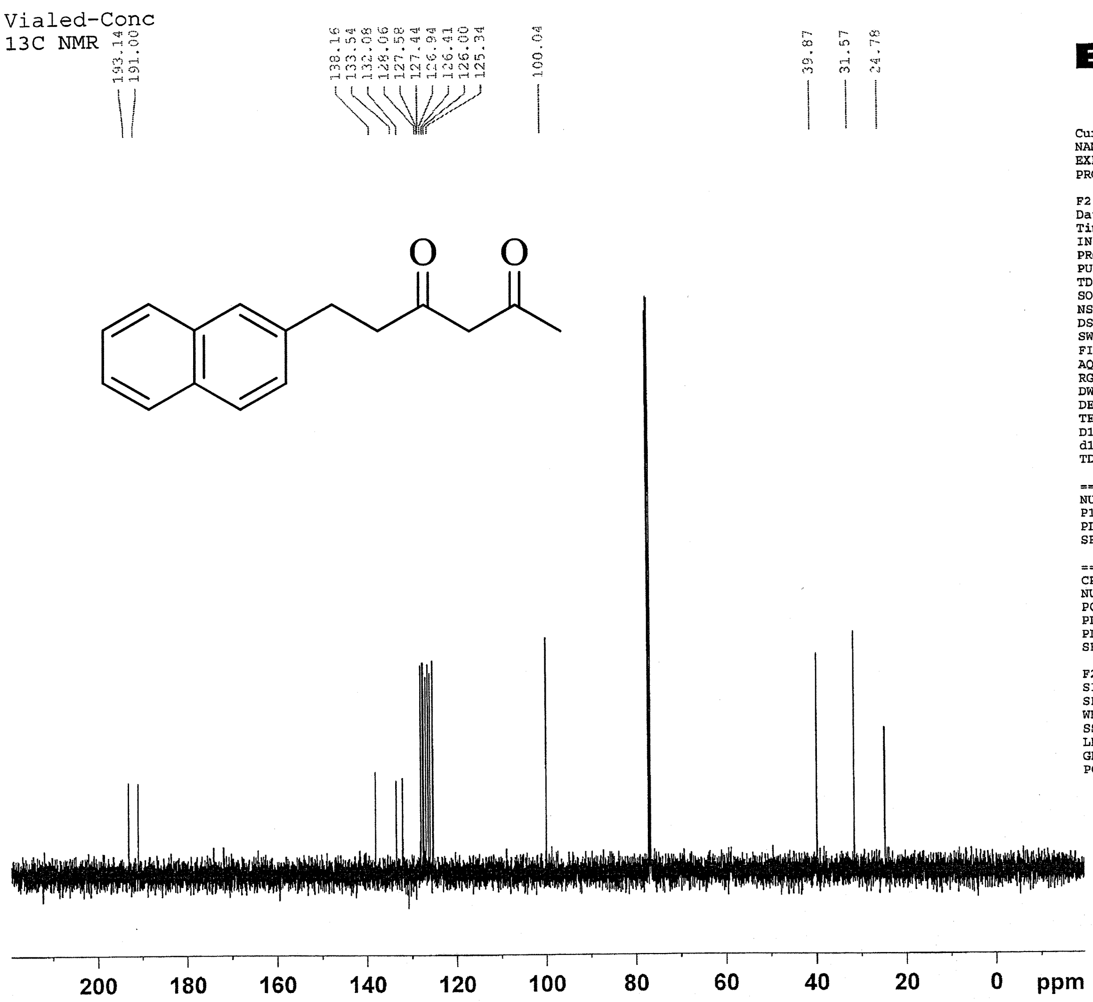


```
Current Data Parameters
NAME      6-Naph-2,4-HexDione-102309
EXPNO                9
PROCNO               1

F2 - Acquisition Parameters
Date_          20091026
Time              16.49
INSTRUM           spect
PROBHD   5 mm BBO BB-1H
PULPROG          zgdc30
TD                65536
SOLVENT           CDCl3
NS                   21
DS                    4
SWH           30030.029 Hz
FIDRES         0.458222 Hz
AQ            1.0912410 sec
RG                 4096
DW               16.650 usec
DE                 7.00 usec
TE                297.5 K
D1           1.00000000 sec
d11          0.03000000 sec
TD0                   1

======== CHANNEL f1 ========
NUC1                13C
P1                 8.60 usec
PL1                1.00 dB
SFO1        125.7703640 MHz

======== CHANNEL f2 ========
CPDPRG2         waltz16
NUC2                 1H
PCPD2            100.00 usec
PL2                1.00 dB
PL12              17.50 dB
SFO2        500.1322150 MHz

F2 - Processing parameters
SI                32768
SF          125.7577984 MHz
WDW                  EM
SSB                   0
LB                 1.00 Hz
GB                    0
PC                 1.40
```

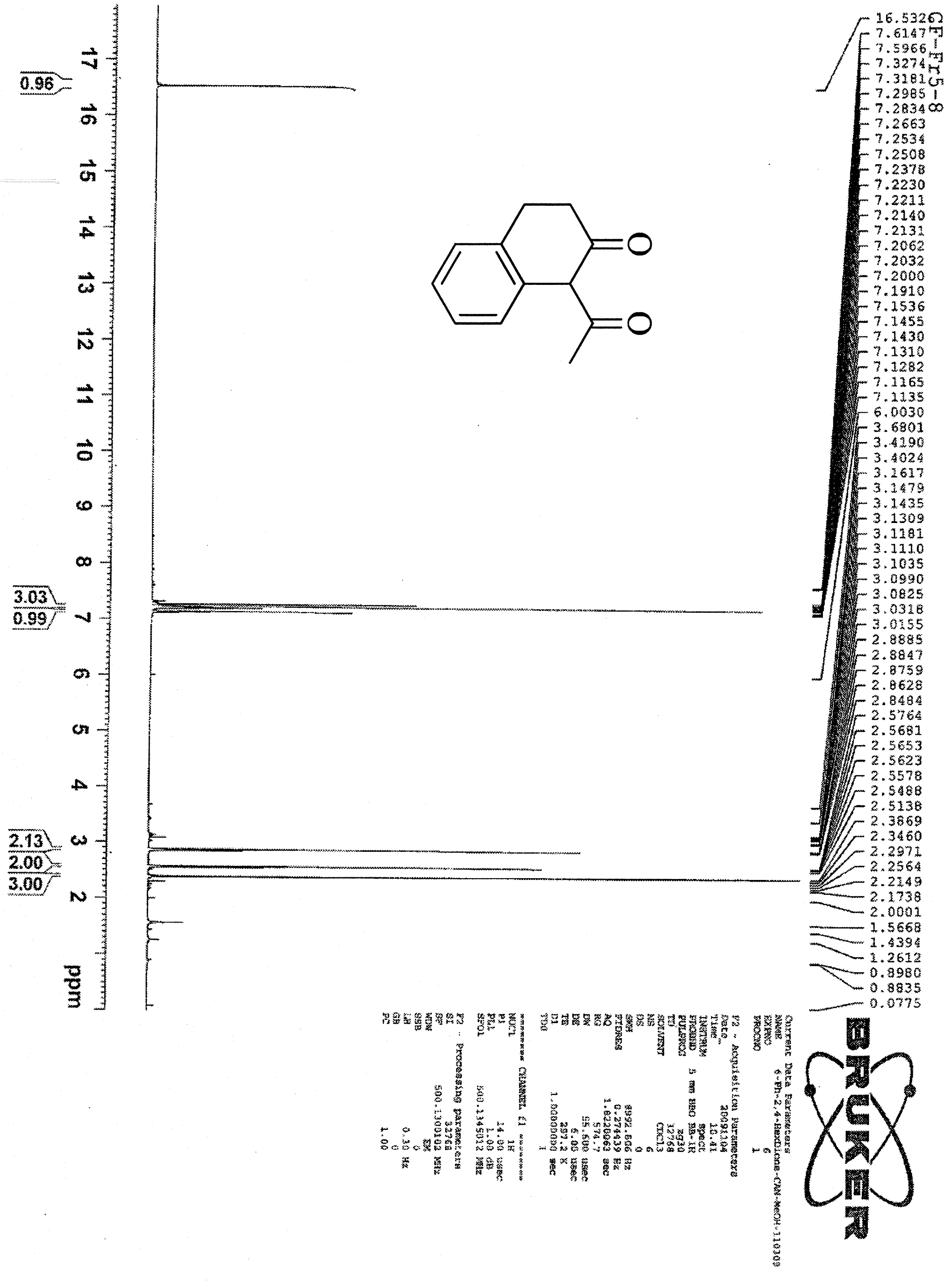
GF-Fr5-8
16.5326
7.6147
7.5966
7.3274
7.3181
7.2985
7.2834
7.2663
7.2534
7.2508
7.2378
7.2230
7.2211
7.2140
7.2131
7.2062
7.2032
7.2000
7.1910
7.1536
7.1455
7.1430
7.1310
7.1282
7.1165
7.1135
6.0030
3.6801
3.4190
3.4024
3.1617
3.1479
3.1435
3.1309
3.1181
3.1110
3.1035
3.0990
3.0825
3.0318
3.0155
2.8885
2.8847
2.8759
2.8628
2.8484
2.5764
2.5681
2.5653
2.5623
2.5578
2.5488
2.5138
2.3869
2.3460
2.2971
2.2564
2.2149
2.1738
2.0001
1.5668
1.4394
1.2612
0.8960
0.8835
0.0775
ppm
0.96
3.03
0.99
2.13
2.00
3.00

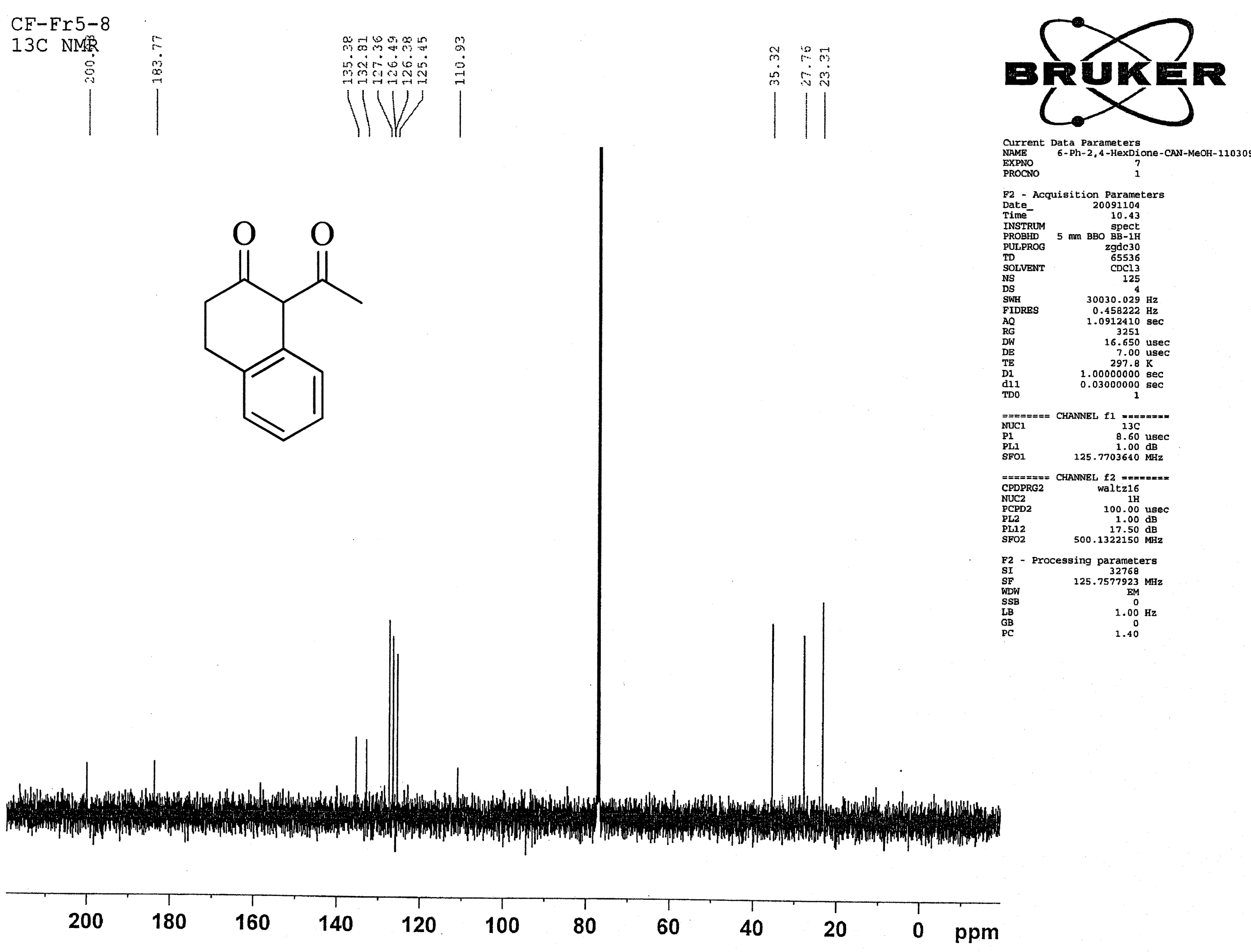
CF-Fr5-8
13C NMR
200.[illegible]
183.77
135.38
132.81
127.36
126.49
126.38
125.45
110.93
35.32
27.76
23.31
200
180
160
140
120
100
80
60
40
20
0
ppm
BRUKER
Current Data Parameters
NAME 6-Ph-2,4-HexDione-CAN-MeOH-110309
EXPNO 7
PROCNO 1
F2 - Acquisition Parameters
Date_ 20091104
Time 10.43
INSTRUM spect
PROBHD 5 mm BBO BB-1H
PULPROG zgdc30
TD 65536
SOLVENT CDCl3
NS 125
DS 4
SWH 30030.029 Hz
FIDRES 0.458222 Hz
AQ 1.0912410 sec
RG 3251
DW 16.650 usec
DE 7.00 usec
TE 297.8 K
D1 1.00000000 sec
d11 0.03000000 sec
TD0 1
======== CHANNEL f1 ========
NUC1 13C
P1 8.60 usec
PL1 1.00 dB
SFO1 125.7703640 MHz
======== CHANNEL f2 ========
CPDPRG2 waltz16
NUC2 1H
PCPD2 100.00 usec
PL2 1.00 dB
PL12 17.50 dB
SFO2 500.1322150 MHz
F2 - Processing parameters
SI 32768
SF 125.7577923 MHz
WDW EM
SSB 0
LB 1.00 Hz
GB 0
PC 1.40

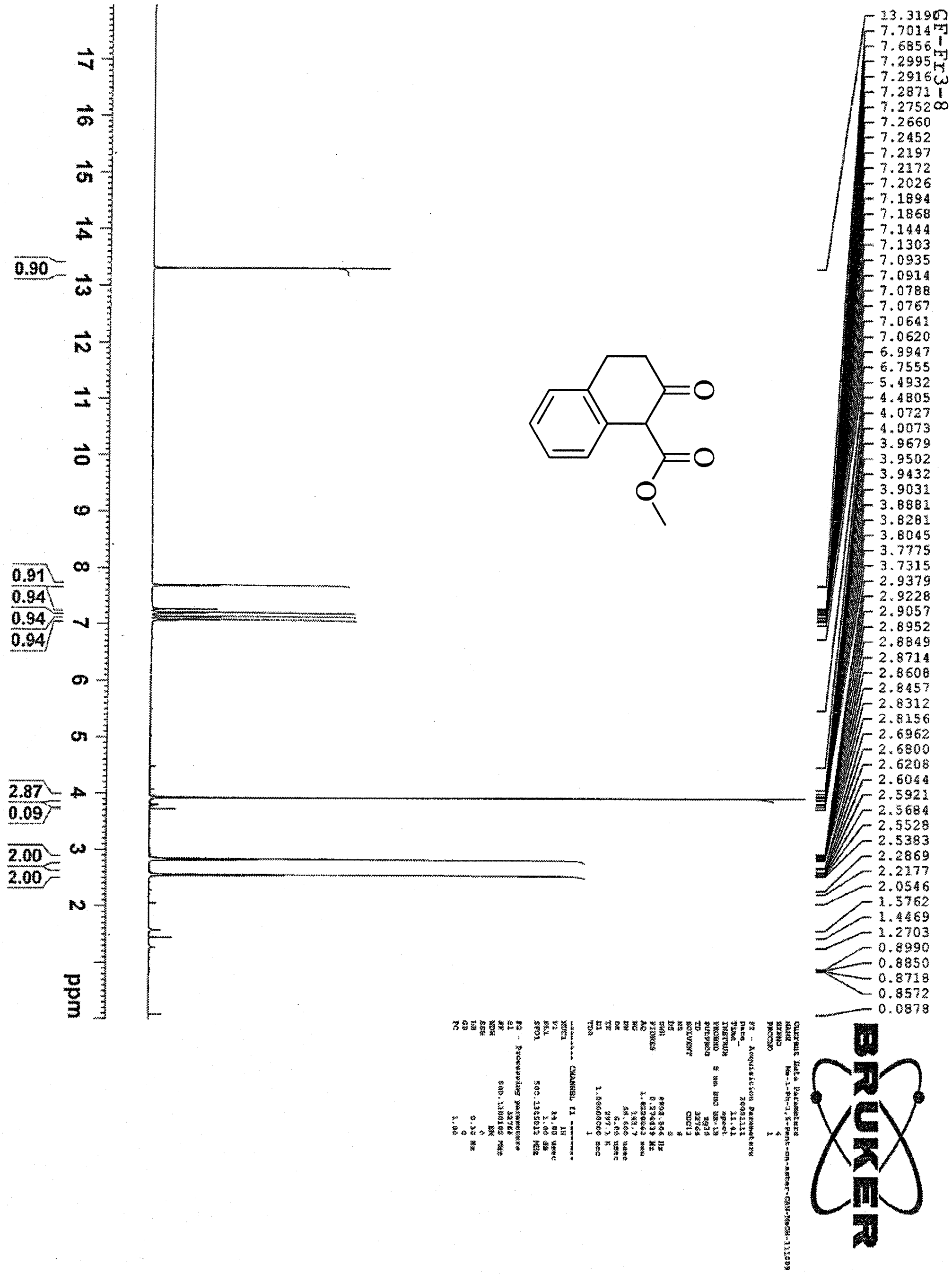
GF-Fr3-8
13.3190
7.7014
7.6856
7.2995
7.2916
7.2871
7.2752
7.2660
7.2452
7.2197
7.2172
7.2026
7.1894
7.1868
7.1444
7.1303
7.0935
7.0914
7.0788
7.0767
7.0641
7.0620
6.9947
6.7555
5.4932
4.4805
4.0727
4.0073
3.9679
3.9502
3.9432
3.9031
3.8881
3.8281
3.8045
3.7775
3.7315
2.9379
2.9228
2.9057
2.8952
2.8849
2.8714
2.8608
2.8457
2.8312
2.8156
2.6962
2.6800
2.6208
2.6044
2.5921
2.5684
2.5528
2.5383
2.2869
2.2177
2.0546
1.5762
1.4469
1.2703
0.8990
0.8850
0.8718
0.8572
0.0878
0.90
0.91
0.94
0.94
0.94
2.87
0.09
2.00
2.00
ppm
BRUKER

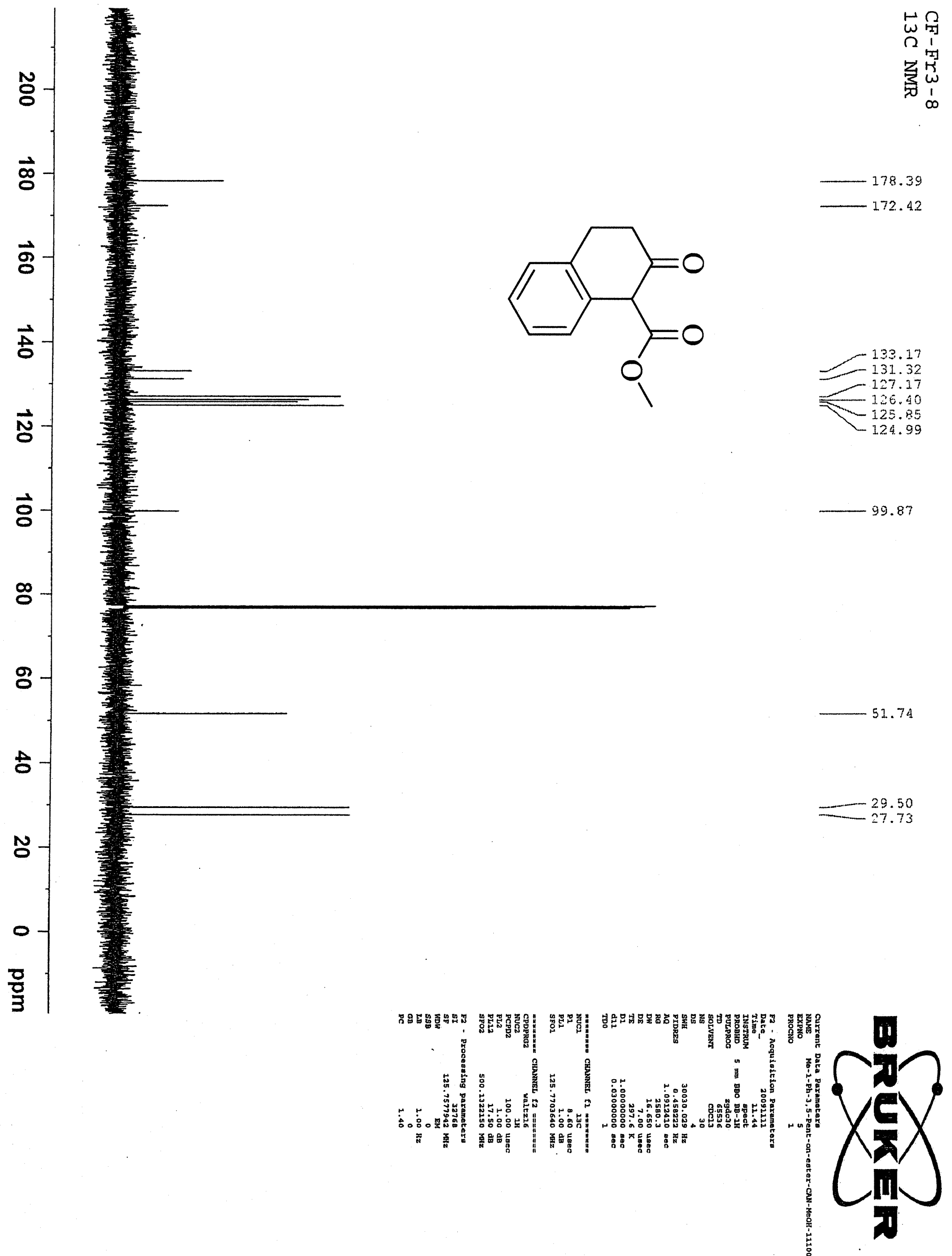
CF-Fr3-8
13C NMR
178.39
172.42
133.17
131.32
127.17
126.40
125.85
124.99
99.87
51.74
29.50
27.73
O
O
O
200
180
160
140
120
100
80
60
40
20
0
ppm
BRUKER
Current Data Parameters
NAME Me-1-Ph-3,5-Pent-on-ester-CAN-MeOH-111009
EXPNO 5
PROCNO 1
F2 - Acquisition Parameters
Date_ 20091111
Time 11.44
INSTRUM spect
PROBHD 5 mm BBO BB-1H
PULPROG zgdc30
TD 65536
SOLVENT CDCl3
NS 30
DS 4
SWH 30030.029 Hz
FIDRES 0.458222 Hz
AQ 1.0912410 sec
RG 2580.3
DW 16.650 usec
DE 7.00 usec
TE 297.6 K
D1 1.00000000 sec
d11 0.03000000 sec
TD0 1
======== CHANNEL f1 ========
NUC1 13C
P1 8.60 usec
PL1 1.00 dB
SFO1 125.7703640 MHz
======== CHANNEL f2 ========
CPDPRG2 waltz16
NUC2 1H
PCPD2 100.00 usec
PL2 1.00 dB
PL12 17.50 dB
SFO2 500.1322150 MHz
F2 - Processing parameters
SI 32768
SF 125.7577942 MHz
WDW EM
SSB 0
LB 1.00 Hz
GB 0
PC 1.40

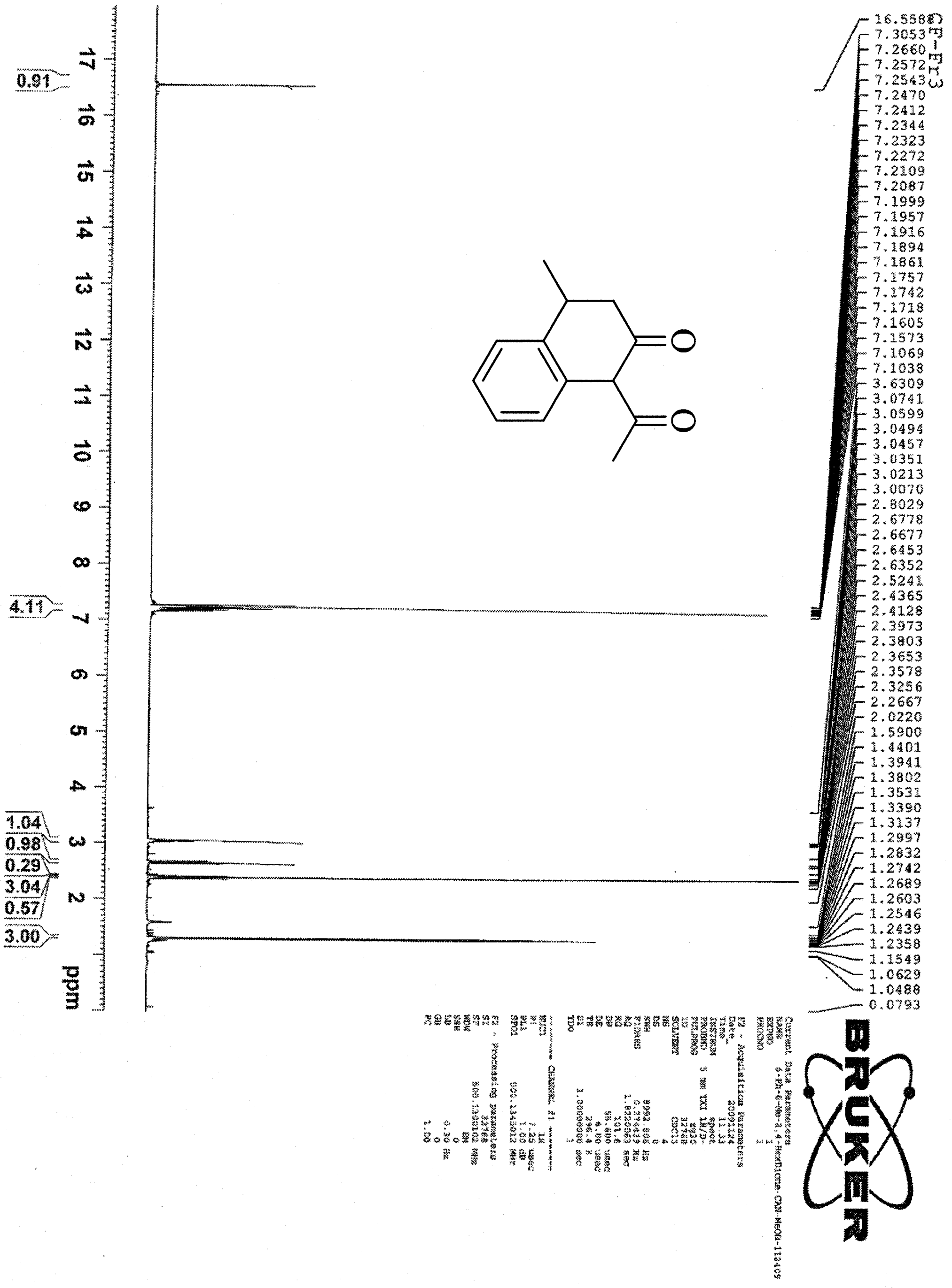

2j
13C NMR

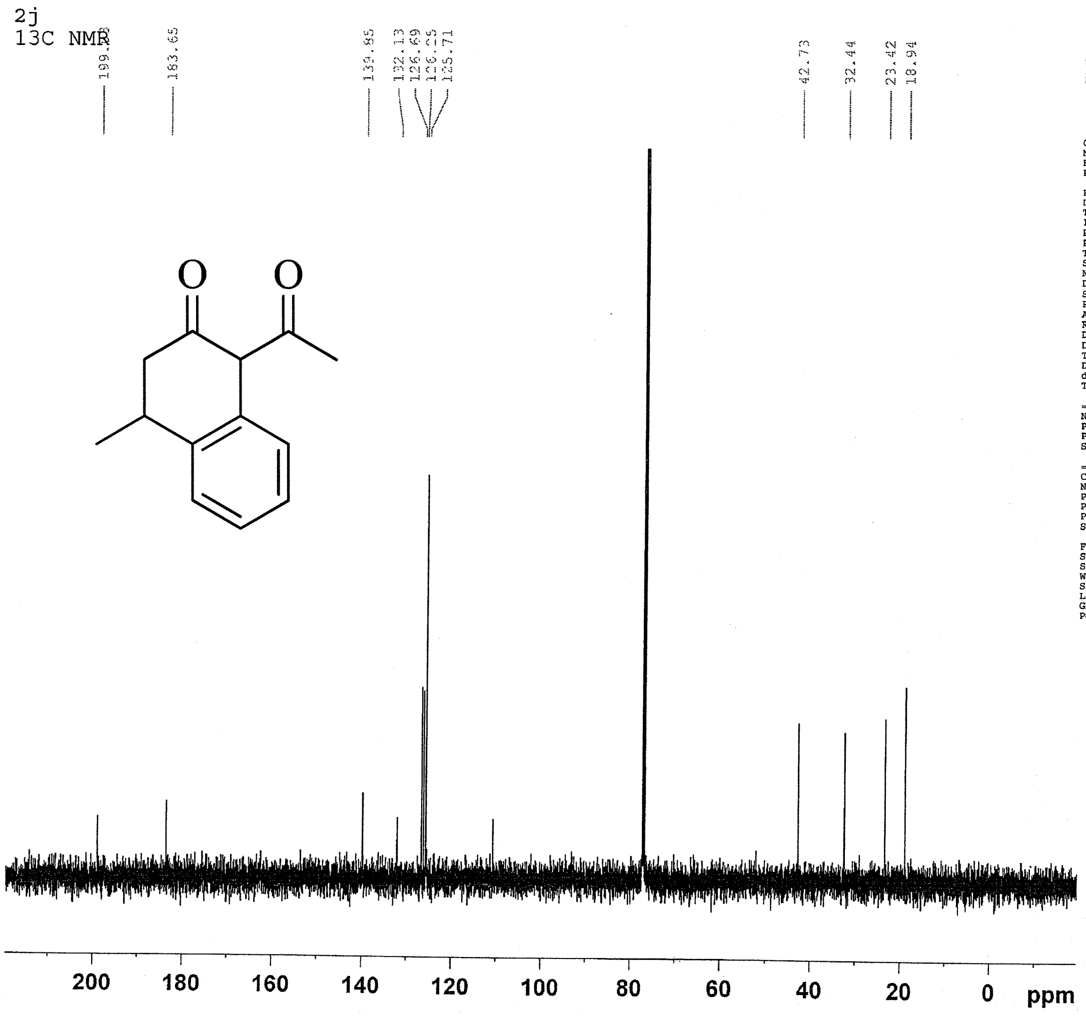

BRUKER

```
Current Data Parameters
NAME      6-Ph-6-Me-2,4-HexDione-CAN-MeOH-112409
EXPNO                 4
PROCNO                1

F2 - Acquisition Parameters
Date_          20091201
Time               9.18
INSTRUM           spect
PROBHD   5 mm BBO BB-1H
PULPROG         zgdc30
TD                65536
SOLVENT           CDCl3
NS                  120
DS                    4
SWH          30030.029 Hz
FIDRES        0.458222 Hz
AQ           1.0912410 sec
RG                 4096
DW              16.650 usec
DE                7.00 usec
TE               297.0 K
D1          1.00000000 sec
d11         0.03000000 sec
TD0                   1

======== CHANNEL f1 ========
NUC1                13C
P1                 8.60 usec
PL1                1.00 dB
SFO1        125.7703640 MHz

======== CHANNEL f2 ========
CPDPRG2         waltz16
NUC2                 1H
PCPD2            100.00 usec
PL2                1.00 dB
PL12              17.50 dB
SFO2        500.1322150 MHz

F2 - Processing parameters
SI                32768
SF          125.7577890 MHz
WDW                  EM
SSB                   0
LB                 1.00 Hz
GB                    0
PC                 1.40
```

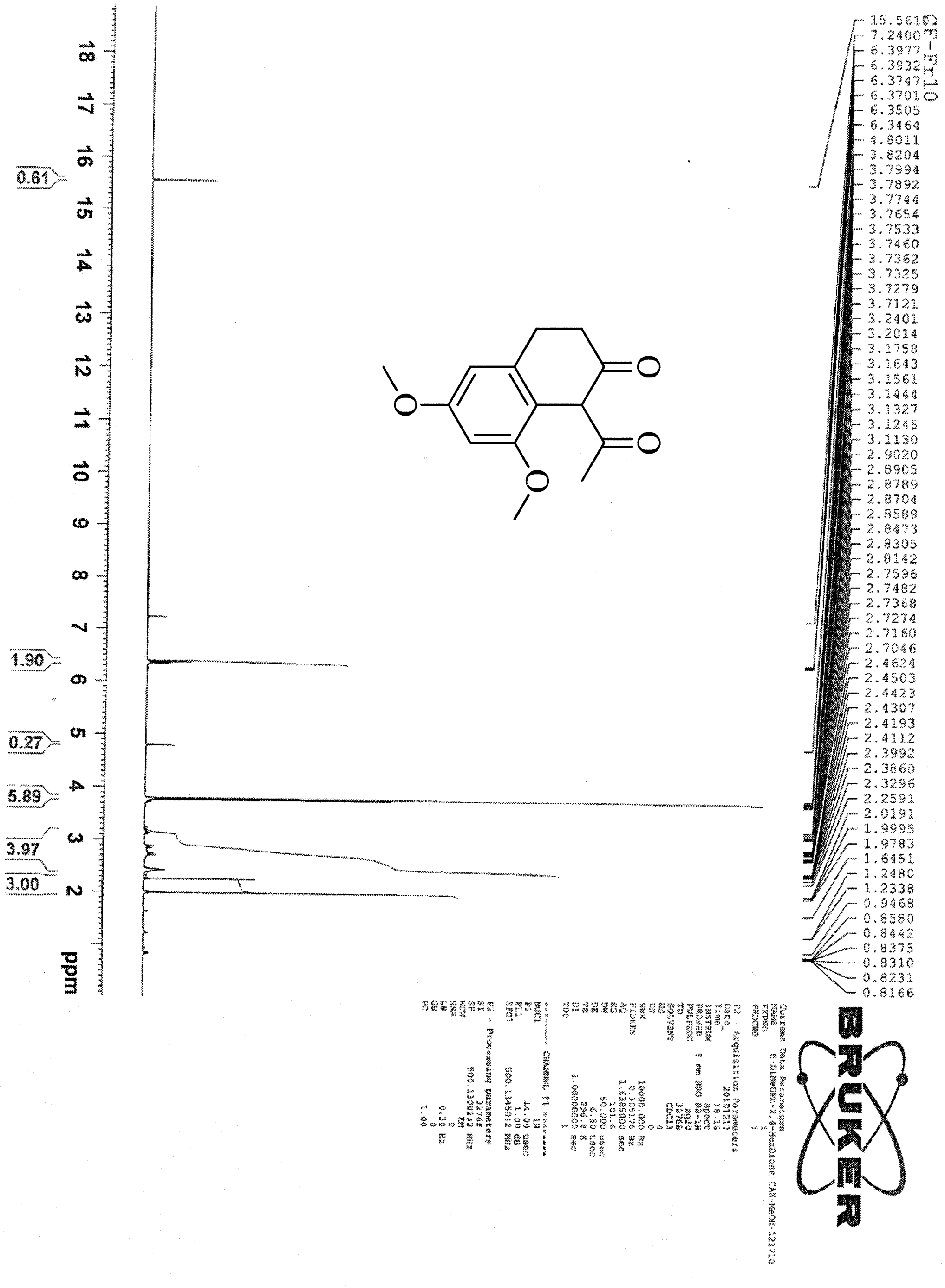

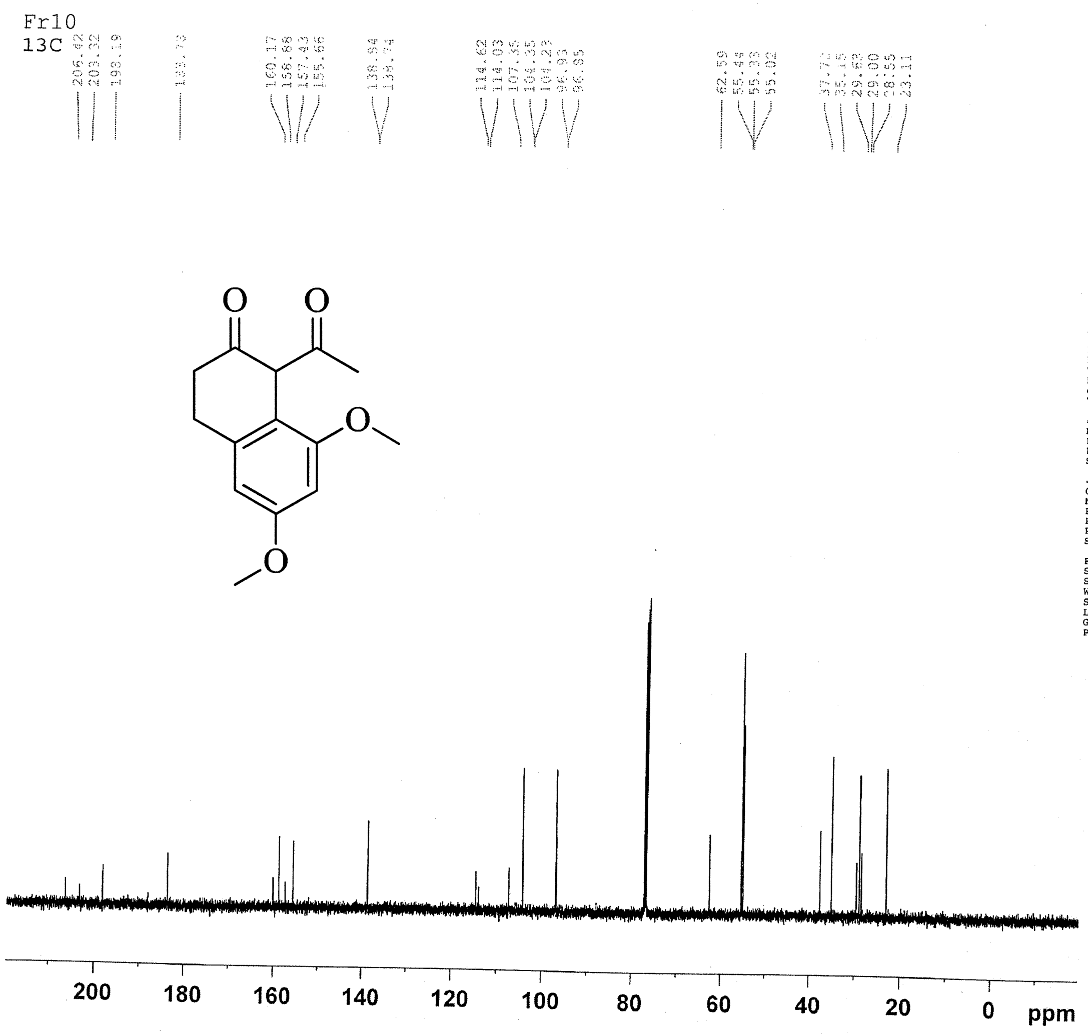


```
Current Data Parameters
NAME     6-DiMeOPh-2,4-HexDione-CAN-MeOH-121710
EXPNO                    2
PROCNO                   1

F2 - Acquisition Parameters
Date_             20101217
Time                 18.20
INSTRUM              spect
PROBHD   5 mm BBO BB-1H
PULPROG            zgdc30
TD                   65536
SOLVENT              CDCl3
NS                      62
DS                       4
SWH          30030.029 Hz
FIDRES        0.458222 Hz
AQ           1.0912410 sec
RG                  7298.2
DW              16.650 usec
DE               7.00 usec
TE               297.2 K
D1          1.00000000 sec
d11         0.03000000 sec
TD0                      1

======== CHANNEL f1 ========
NUC1                   13C
P1                8.60 usec
PL1               1.00 dB
SFO1        125.7703640 MHz

======== CHANNEL f2 ========
CPDPRG2            waltz16
NUC2                    1H
PCPD2           100.00 usec
PL2                1.00 dB
PL12              17.50 dB
SFO2        500.1322150 MHz

F2 - Processing parameters
SI                   32768
SF          125.7577983 MHz
WDW                     EM
SSB                      0
LB                 1.00 Hz
GB                       0
PC                    1.40
```

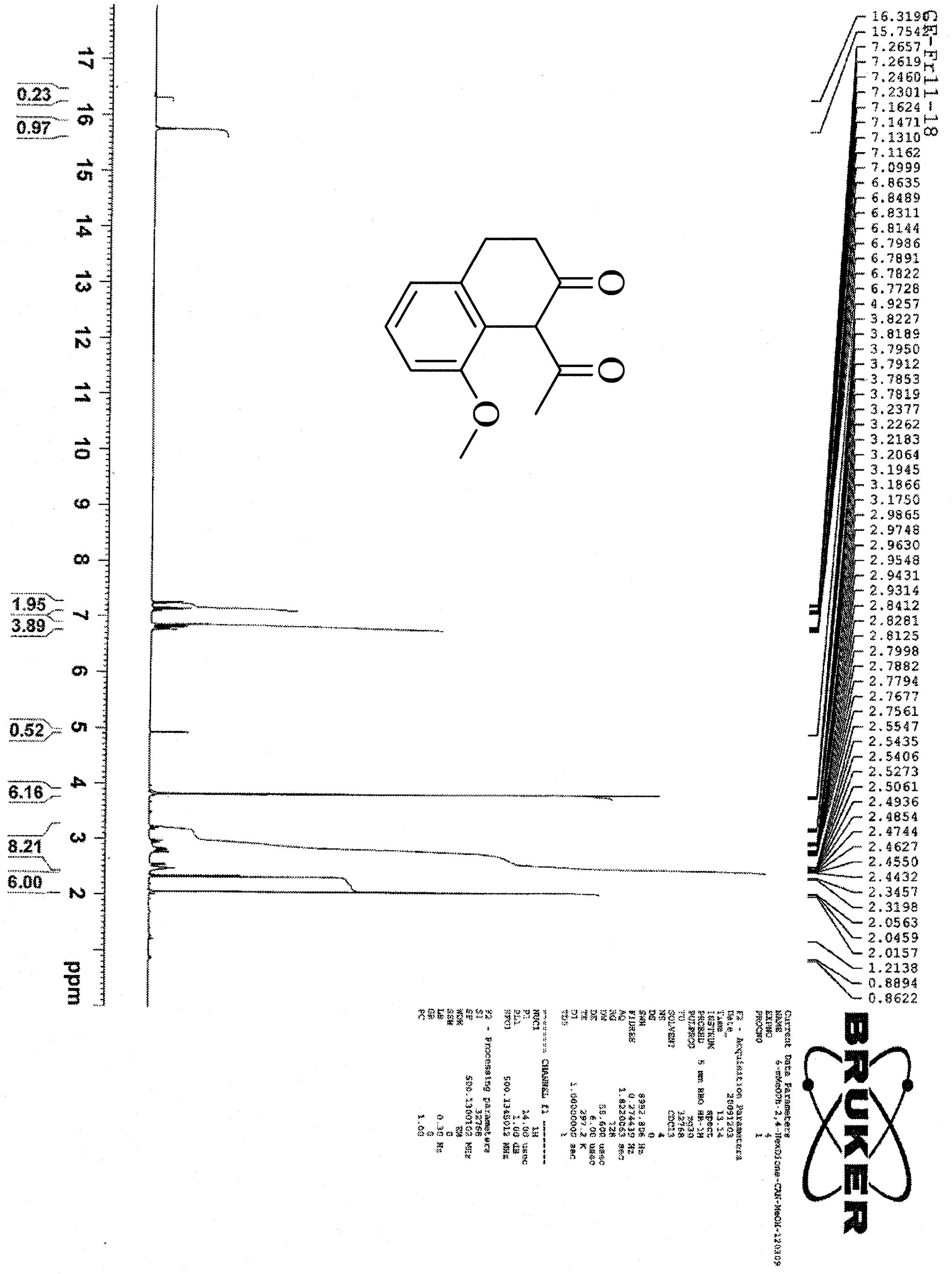
GE-Fr11-18
16.3196
15.7548
7.2657
7.2619
7.2460
7.2301
7.1624
7.1471
7.1310
7.1162
7.0999
6.8635
6.8489
6.8311
6.8144
6.7986
6.7891
6.7822
6.7728
4.9257
3.8227
3.8189
3.7950
3.7912
3.7853
3.7819
3.2377
3.2262
3.2183
3.2064
3.1945
3.1866
3.1750
2.9865
2.9748
2.9630
2.9548
2.9431
2.9314
2.8412
2.8281
2.8125
2.7998
2.7882
2.7794
2.7677
2.7561
2.5547
2.5435
2.5406
2.5273
2.5061
2.4936
2.4854
2.4744
2.4627
2.4550
2.4432
2.3457
2.3198
2.0563
2.0459
2.0157
1.2138
0.8894
0.8622
0.23
0.97
1.95
3.89
0.52
6.16
8.21
6.00
ppm
BRUKER

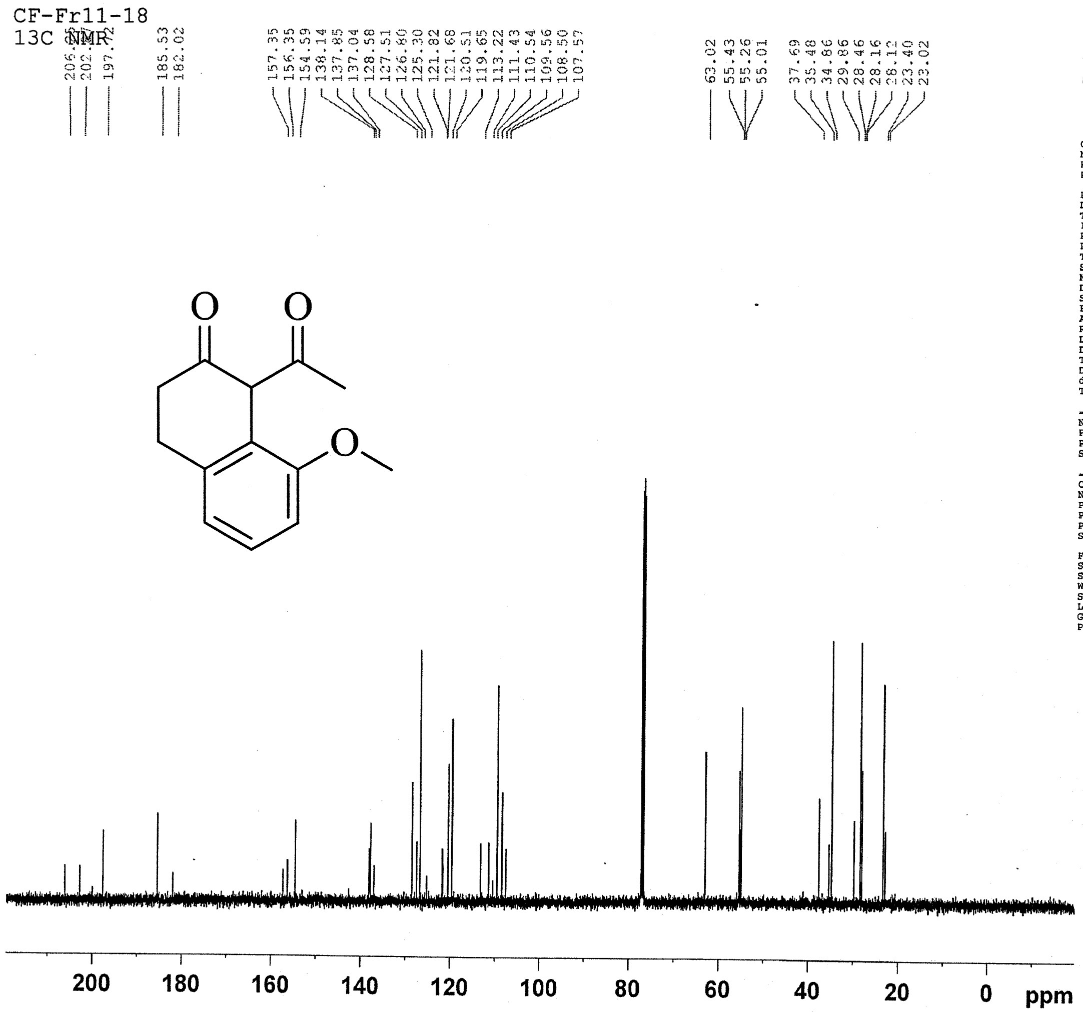

Current Data Parameters	
NAME	6-mMeOPh-2,4-HexDione-CAN-MeOH-120309
EXPNO	5
PROCNO	1

F2 - Acquisition Parameters	
Date_	20091203
Time	13.18
INSTRUM	spect
PROBHD	5 mm BBO BB-1H
PULPROG	zgdc30
TD	65536
SOLVENT	CDCl3
NS	135
DS	4
SWH	30030.029 Hz
FIDRES	0.458222 Hz
AQ	1.0912410 sec
RG	4096
DW	16.650 usec
DE	7.00 usec
TE	297.8 K
D1	1.00000000 sec
d11	0.03000000 sec
TD0	1

======== CHANNEL f1 ========	
NUC1	13C
P1	8.60 usec
PL1	1.00 dB
SFO1	125.7703640 MHz

======== CHANNEL f2 ========	
CPDPRG2	waltz16
NUC2	1H
PCPD2	100.00 usec
PL2	1.00 dB
PL12	17.50 dB
SFO2	500.1322150 MHz

F2 - Processing parameters	
SI	32768
SF	125.7577973 MHz
WDW	EM
SSB	0
LB	1.00 Hz
GB	0
PC	1.40

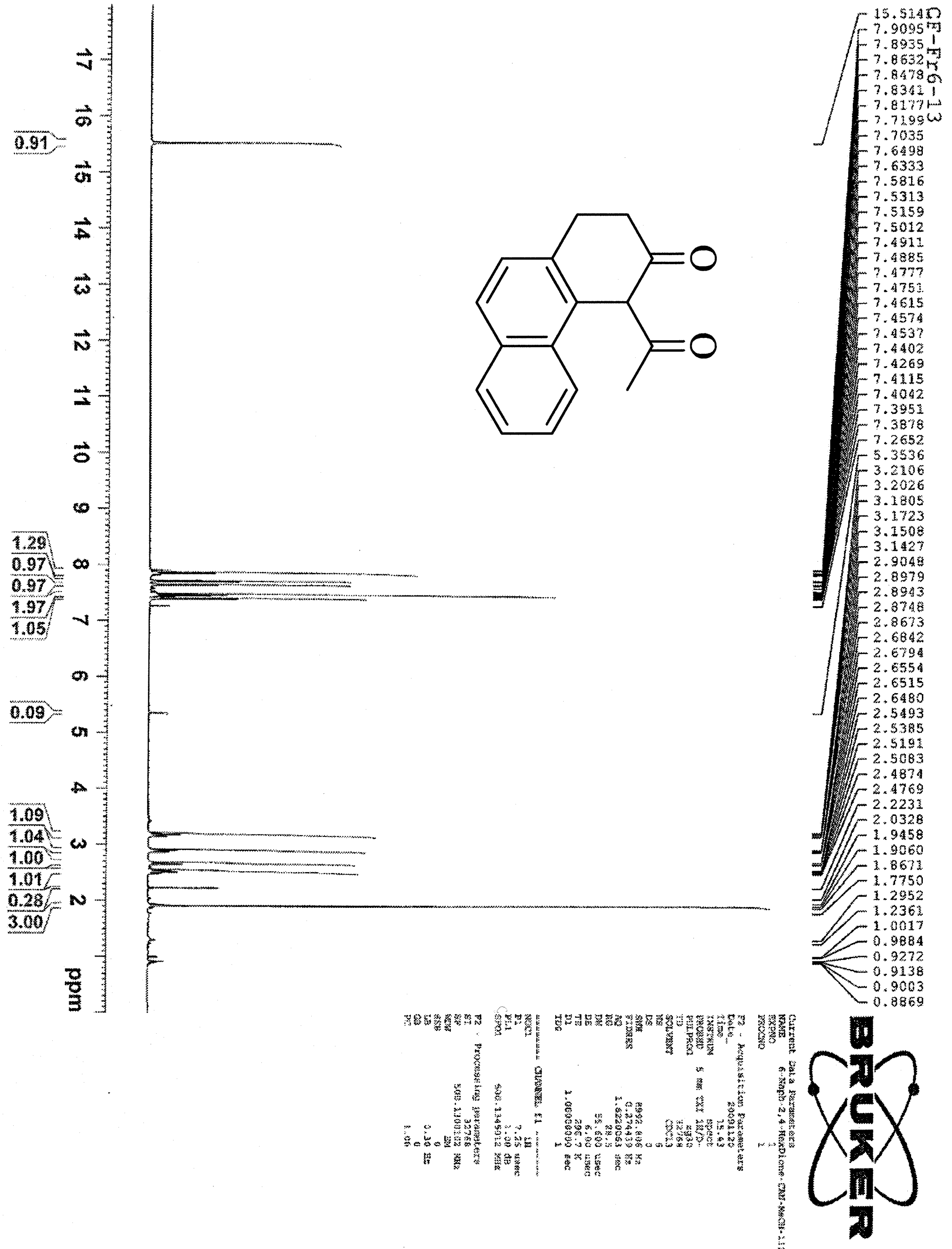

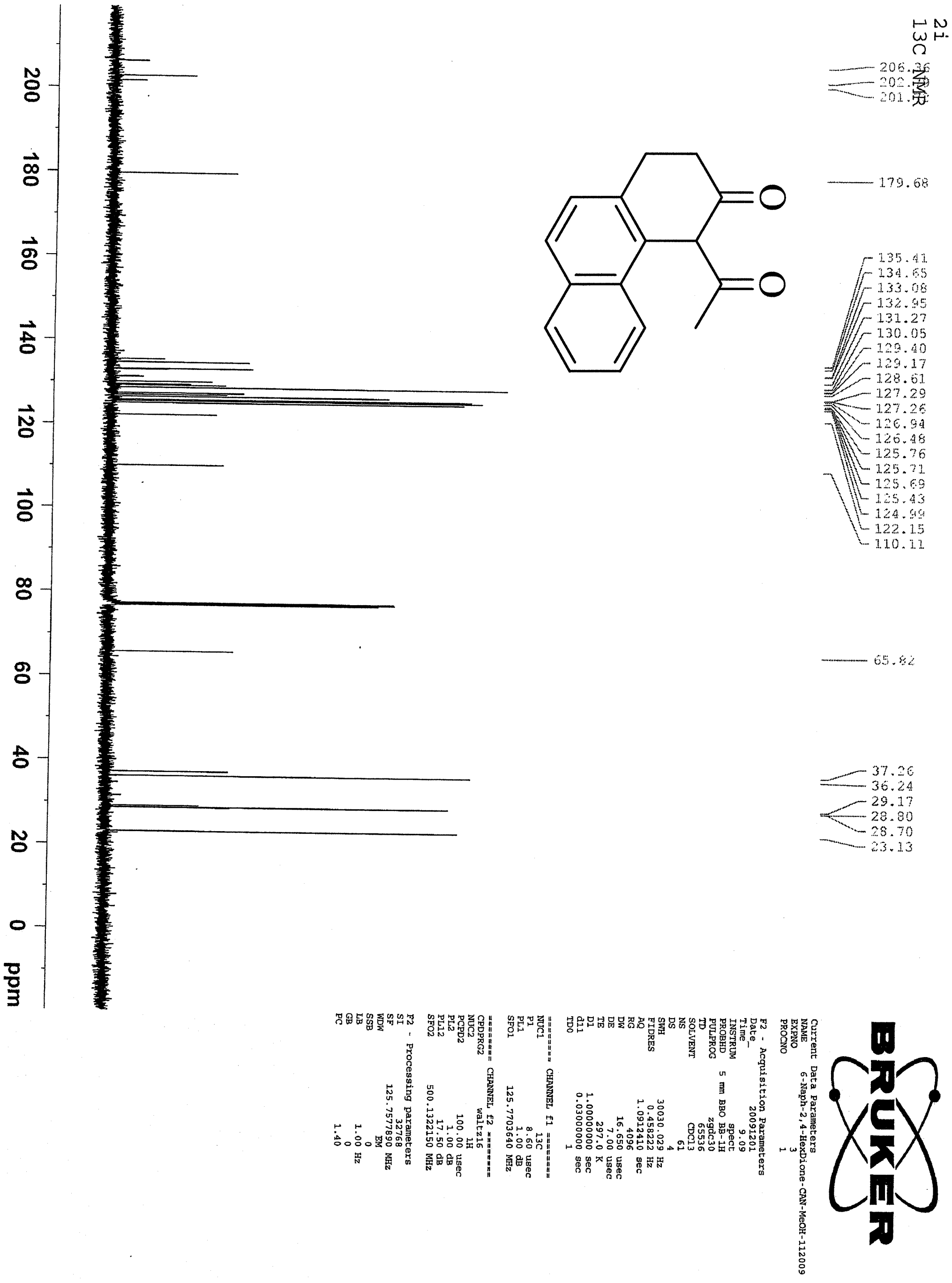
2i
13C NMR
206.36
202.[illegible]
201.[illegible]
179.68
135.41
134.65
133.08
132.95
131.27
130.05
129.40
129.17
128.61
127.29
127.26
126.94
126.48
125.76
125.71
125.69
125.43
124.99
122.15
110.11
65.82
37.26
36.24
29.17
28.80
28.70
23.13
BRUKER
Current Data Parameters
NAME 6-Naph-2,4-HexDione-CAN-MeOH-112009
EXPNO 3
PROCNO 1
F2 - Acquisition Parameters
Date_ 20091201
Time 9.09
INSTRUM spect
PROBHD 5 mm BBO BB-1H
PULPROG zgdc30
TD 65536
SOLVENT CDCl3
NS 61
DS 4
SWH 30030.029 Hz
FIDRES 0.458222 Hz
AQ 1.0912410 sec
RG 4096
DW 16.650 usec
DE 7.00 usec
TE 297.0 K
D1 1.00000000 sec
d11 0.03000000 sec
TD0 1
======== CHANNEL f1 ========
NUC1 13C
P1 8.60 usec
PL1 1.00 dB
SFO1 125.7703640 MHz
======== CHANNEL f2 ========
CPDPRG2 waltz16
NUC2 1H
PCPD2 100.00 usec
PL2 1.00 dB
PL12 17.50 dB
SFO2 500.1322150 MHz
F2 - Processing parameters
SI 32768
SF 125.7577890 MHz
WDW EM
SSB 0
LB 1.00 Hz
GB 0
PC 1.40
200
180
160
140
120
100
80
60
40
20
0
ppm
